# A complex network perspective on brain disease

**DOI:** 10.1111/brv.70086

**Published:** 2025-10-15

**Authors:** David Papo, Javier M. Buldú

**Affiliations:** ^1^ Department of Neuroscience and Rehabilitation, Section of Physiology University of Ferrara Via Fossato di Mortara, 19 Ferrara 44121 Italy; ^2^ Center for Translational Neurophysiology, Fondazione Istituto Italiano di Tecnologia Via Fossato di Mortara, 19 Ferrara 44121 Italy; ^3^ Complex Systems Group & G.I.S.C., Universidad Rey Juan Carlos Calle Tulipan s/n. 28933 Mostoles Spain

**Keywords:** dynamical disease, complex networks, pathoconnectomics, brain topology, degeneracy, resilience and vulnerability, evolvability, neutral networks

## Abstract

If brain anatomy and dynamics have a complex network structure as it has become standard to posit, it is reasonable to assume that such a structure should play a key role not only in brain function but also in brain dysfunction. However, exactly how network structure is implicated in brain damage and whether at least some pathologies can be thought of as ‘network diseases’ is not yet clear. Here we discuss ways in which a complex network representation can help in characterising brain pathology, but also in assessing subjects' vulnerability to and likelihood of recovery from disease. We show how the way disease is defined is related to the way function is defined and this, in turn, determines which network property may be functionally relevant to brain disease. Thus, addressing brain disease ‘networkness’ may shed light not only on brain pathology, with potential clinical implications, but also on functional brain activity, and what is functional in it.

## I. INTRODUCTION

Network neuroscience characterises brain anatomy, dynamics and ultimately function by endowing them with a network representation and describing them in terms of properties of this representation (Bullmore & Sporns, 2009). A network is a particular structure, i.e. a set of objects with some additional feature on the set, whose objects are nodes and whose additional feature is a relation among the nodes which defines links relating them (Boccaletti *et al*., 2006). All the information in a networked system is encoded in the relational pattern, whose properties allow not only describing the system's relational structure at all scales but also, at least in principle, explaining properties such as its efficiency and robustness (Papo *et al*., 2014), and ultimately conceiving of ways it can be steered towards desirable states or away from undesirable ones (Liu & Barabási, 2016; Papo, 2019b).

Whether the brain actually behaves as a complex network or whether such a structure merely constitutes a convenient representation is a fundamental yet still poorly understood question (Papo & Buldú, 2024). Showing that at least some brain diseases, which we shall call network diseases, are somehow related to brain network structure would constitute an important step towards a proof of genuine brain networkness.

Characterising the nature of disease, identifying ways in which it may become observable, even in the absence of behavioural symptoms, quantifying its severity, characterising, and predicting the way it starts and evolves and ultimately finding ways in which it can be acted upon are fundamental theoretical and clinical questions. Herein, we evaluate how a network representation may help address these questions and in defining and understanding brain disease. Importantly, we do not aim at characterising the network structure of any given pathology, but rather at evaluating the way network structure can help address generic issues related to brain pathology, from the phenomenology of disease to subjects' vulnerability to or ability to recover from it.

To delineate the role of network structure in brain disease, we first discuss possible meanings of disease, and then briefly review ways in which the brain can be endowed with a complex network structure. Finally, we analyse how complex networks can be used to make sense of various aspects of brain pathology and how various neurological and psychiatric pathologies can be understood in terms of such a structure.

Throughout, we differentiate between two pairs of terms often confusingly used as synonyms in the network neuroscience literature: structure and anatomy, and dynamics and function. We use the term *structure*, which equally applies to anatomy and dynamics, to designate a set of entities and their relations. In particular, we prefer the expression *anatomical* to *structural network*, as it more clearly designates anatomy equipped with a particular structure encoded in a graph representation. On the other hand, as we further explain below, we consider genuine functional brain activity to be distinct from bare brain dynamics in that it is the particular aspect of brain activity that is used to accomplish a given function.

## II. DEFINING DISEASE

### (1) The semantic field of disease

There are a number of conceptually different ways to understand disease. This variety is illustrated by the semantic field of disease and particularly by the etymology of words thought of as its synonyms (Lalande, 1927; Canguilhem, 1966). The terms *disease* (lack of ease or comfort), *pathology* (from the Greek *πάϑος*, suffering) and *ailment* (which causes to ail, to suffer) refer to effects, particularly to the associated pain. From the same legal semantic field, the terms *damage* (from the Latin *damnum*), *lesion* (from the Latin *laedo*, to injur) and *injury* (from the Latin *iuris*, law, the prefix *in*‐ expressing negation) refer to the consequences of a violation. The term *abnormal*, initially legal in nature, also refers to a norm violation. Originally expressing a judgement of value rather than a description (Canguilhem, 1966), in the context of disease, it implicitly refers to a different (i.e. healthy) state. Finally, while the terms *disorder* and *anomaly* (from the Greek *ομαλός*, smooth, rather than from *νόμος*, law) refer to structural properties, in mathematical terms respectively symmetry and regularity/differentiability, an explicitly functional aspect underlies the term *malady* (from the Latin *malaptus*). Finally, the term *condition* (from the Latin verb *condo*, to put together, to build), often resorted to in order to avoid unwanted associations induced by these various etymologies, designates an identifiable state resulting from the assembly of various parts or the way this comes into being.

It is also interesting to look at the semantic field of the proneness to disease. The term *vulnerability* comes from the Latin word *vulnus*, injury, itself coming from the Latin verb *vello*, to tear or to lacerate, while *fragility* comes from the Latin verb *frango*, to break. The adjective *critical* and the corresponding noun *crisis* (from the Greek noun *κρίσις* and the verb *κρίνειν*) originally mainly designated separation (of wheat grain from straw and chaff), before acquiring by translation the meaning of choosing (Chantraine, 1968). Thus, *crisis* designates a choice made after a separation, hence perhaps the associated negative meaning stemming from the need to choose to separate something from something else.

Correspondingly, there is more than one way of conceiving of brain disease from a system‐level perspective: one consists in characterising its phenomenology in terms of its consequences on healthy brain anatomy and dynamics. In this sense, characterising brain pathology entails defining healthy brain function, which can be represented as a structure or as a field, and pathology as some perturbation of it, respectively with a mass (e.g. a mass‐invading tumour) or as a dynamical system, in the simplest case, an impulse. A dual way involves defining disease as an emergent property of brain dynamics under certain conditions, e.g. within given ranges of control parameter values. Brain disease can also be seen as a process evolving on an underlying structure, with which it may or may not interact. This can for instance take the form of a generic spreading process, e.g. a transport process (of a diffusive kind if what is modelled is activity propagation, or of an advective one if mass is transported).

#### (a) Brain–disease relationships

These approaches implicitly suggest various possible relationships between brain and disease. In an ontological approach, brain and disease may constitute separate entities, each with its own mass and corresponding spatial localisation. If these two entities interact, disease can be seen as a perturbation of an otherwise intact structure. In a different approach, disease is a particular state of the system. Such an approach can be seen to trace back to Hippocrates' dynamic conceptualisation of disease, with exogenous factors only playing the role of context rather than cause (Canguilhem, 1966). In more modern terms, disease may arise when a physiological system operates in a range of control parameters that induces a dynamic regime associated with functional consequences deemed pathological (Mackey & Glass, 1977; Glass & Mackey, 1979, 1988; Glass, 2015). In this sense, pathology is an aspect of the brain's repertoire, which is expressed under certain conditions (McIntosh & Jirsa, 2019).

The dynamic understanding of disease can be approached through two complementary angles, respectively related to the nature of the regime itself and to its pathogenesis, i.e. the mechanisms through which it develops, progresses, and either persists or is resolved. These approaches give rise to some fundamental questions. On the one hand, when and how can disease be described in dynamical terms? Is the pathological regime qualitatively different from the one associated with healthy brain functioning? If disease is associated with qualitative functional changes, then it can be thought of as forcing symmetry breaking (in Hippocrates' approach, it breaks equilibrium between elements). For instance, in Alzheimer's disease (AD), the loss of dynamical connections and the increased randomness in network structure are key indicators of impaired brain function (Tijms *et al*., 2013). On the other hand, several mechanisms can potentially underlie pathogenesis. When thinking of disease as equilibrium breakdown, the most obvious of such mechanisms is homeostasis, i.e. the self‐regulating processes ensuring the maintenance of the best conditions for the system's operation. When the brain deviates from this condition, as in the case of epilepsy (van Diessen *et al*., 2013), dysfunction arises. Furthermore, pathology can be thought of as a change in the system's response function. For instance, the allostasis (stability through change) model defines health as optimal responsiveness to fluctuations in demand, and disease as shrinkage of adaptive variation (Sterling, 2020). More generally, disease may be associated with loss of complexity rather than regularity (West, 2010).

### (2) Disease dynamics

If the brain is thought of as a dynamical system, its evolution to disease can be thought of as a process. The disease process can be temporally segmented, e.g. identifying a healthy state (or a state in which the disease is under control, in an incubation or a chronic period) and a pathological state, separated by a pre‐pathological state (Scheffer *et al*., 2024). The dynamics underlying each of these states can be associated with a complex space, possibly with identifiable subspaces induced by the generic metastable dynamics of the brain (Roberts *et al*., 2019; Recanatesi *et al*., 2022). The disease process is ultimately characterised by the way the dynamics tends to flow towards, around or away from a given state and by the corresponding basin of attraction, i.e. the set of initial conditions that lead to that particular state under the dynamics.

Characterising neurological and psychiatric disorders and their trajectory requires understanding how the dynamics moves the system from one state to the other under the action of external drives, e.g. how smoothly or abruptly the system may change, or how much the system resists such changes at any given state. The following substantial questions arise naturally: how prone or vulnerable is a given system to damage? How easy is it to push it away from a pathological state? How does it revert to its prior configuration after a perturbation? Perhaps more fundamentally, how much damage is needed to compromise function?

### (3) Disease, brain function, and functional space

A common defining factor of disease is that it is associated with functional impairment, often accompanied by behaviourally observable consequences. Impairment may range from decreased efficiency to functional breakdown. This corresponds to Sigerist's (1932) understanding of pathology as physiology with obstacles. In this approach, pathology can be derived from normal function, from which it is separated by an alien element which complexifies but does not qualitatively alter it (Canguilhem, 1966). In terms of structure, disease can then be seen as an obstruction to some part of the functional space.

**Fig. 1:**
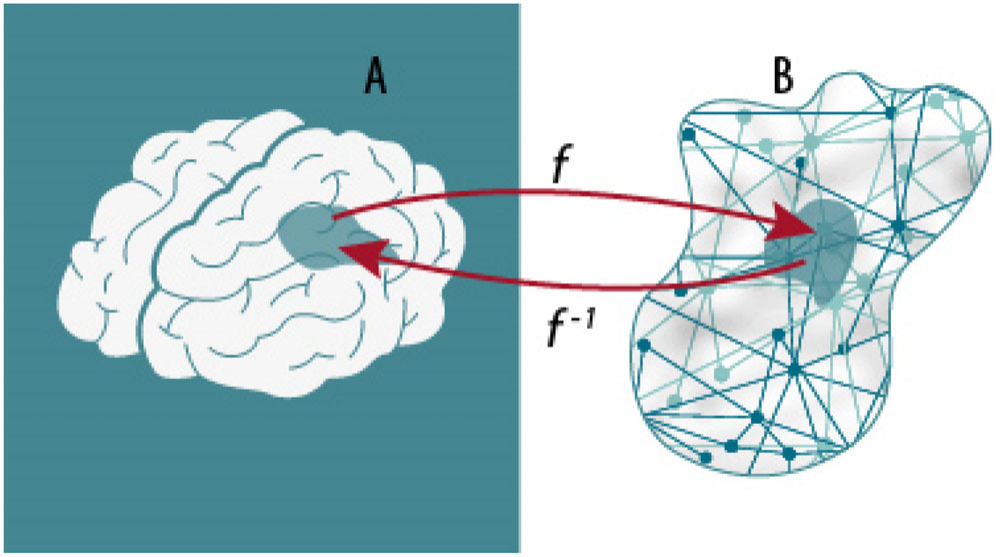
Functional brain activity should not be equated with bare brain dynamics. Functional brain activity results from a complex projection f and its inverse $f^{-1}$ from the structure $S_{A}$ of the neurophysiological space A to the structure $S_{B}$ defined on the abstract space B of cognitive functions made observable by some set of performance measures. Ultimately, parcellation in one space is used to define parcellations in the other (Korhonen *et al*., 2021).

Defining brain function and what is functional in brain activity are two only seemingly easy tasks. While brain dynamics and function are often used interchangeably, functional brain activity can be thought of as a particular aspect of brain dynamics, corresponding to the set of all neurophysiological configurations that generate a given function. Genuinely functional activity results from a complex relation between two structures: the structure of the neurophysiological space together with its equivalence classes (possibly made observable by some recording technique) and the structure of an abstract space of functions (made observable by some performance measure). Subdivisions in one space, corresponding to equivalence classes with identical properties, are used to define subdivisions in the other (Papo, 2019a; Papo & Buldú, 2024) (Fig. 1).

Nearness and neighbourhood relations may qualitatively differ when considering function rather than bare dynamics. The definition of other properties such as path‐dependence and robustness (Lesne, 2008) may also vary in the dynamics‐to‐function transition (Papo, 2019a). All these factors may give rise to a space with non‐trivial structure (Stadler *et al*., 2001). Phenomenology including intermittency, redundancy, degeneracy, whereby structurally dissimilar components can perform similar functions under certain conditions but different ones under others (Edelman & Gally, 2001), and functional robustness can be understood in terms of the combination of accessibility in the neurophysiological space, i.e. which transitions are realisable in the neighbourhood of underlying neuronal configurations, and the presence of large neutral regions in the space of system configurations or parameters that give rise to equivalent phenotypic behaviour, so that changes in such spaces have no consequence on the functional space onto which they are projected (Stadler *et al*., 2001; Stadler & Stadler, 2006; Doyle & Csete, 2011; Papo, 2019a).

### (4) Vulnerability and resilience

The impact of disease on brain anatomy, dynamics and function depends on the system's ability to resist external fields or boundary elements acting on the brain at various scales. Various diseases result from the balancing of interactions between the system and the environment, mutations, and homeostatic mechanisms keeping the system within physiological regimes (Nijhout, Best & Reed, 2019). Brain disorder dynamics is often explained as the result of the interaction between a predisposing vulnerability (or diathesis) (e.g. genetic background, predisposing conditions, age), and a perturbation (or stress) caused by exogenous factors (e.g. psychological or physical trauma), while the risk of neurological and psychiatric disorders is also related the dynamic resilience of the healthy state (Scheffer *et al*., 2024).

Resilience can be understood in a number of ways (Fisher, 2015; Urruty, Tailliez‐Lefebvre & Huyghe, 2016; Liu *et al*., 2022; Krakovská, Kuehn & Longo, 2024). For instance, it may designate the degree to which a system withstands perturbations or errors, resists change and avoids collapsing or being driven towards a qualitatively different stable state (Holling, 1973). It has been suggested that even far from phase transitions, spontaneous activity's neuronal avalanches may be neutral with respect to perturbations (Martinello *et al*., 2017). In the context of brain pathology, robustness may quantify the extent to which the brain can withstand damage arising, e.g. from tumours, trauma, or stroke (Farooq *et al*., 2019). Resilience can also be understood in terms of the system's ability to revert to the original stable state after a perturbation. In this sense, resilience is defined by the relaxation time to the previous stable regime (Holling, 1996), although this notion becomes problematic in an inherently multi‐ or metastable system. Resilience can also be understood in terms of a system's ability to adapt or transform in response to extreme shocks (Holling, Gunderson & Peterson, 2002), and its ability to reorganise around alternative states once pushed away from the original state (Gunderson, 2000). This includes the ability to cope through rapid response and active recovery strategies. To some approximation, the same conceptual framework can be used to make sense of disease resilience, i.e. the tendency of disease to persist.

A system's resilience is defined with respect to a given property, for example some topological network property, or dynamical regime, e.g. balanced state, or some information‐related property, e.g. informational capacity (Koetter & Médard, 2003) or, more generally, the ability to perform some prescribed task, e.g. information encoding, and against a given set of perturbations (Kitano, 2007), e.g. node or link perturbation. The system may respond to perturbations by changing some but not all properties in order to preserve some other ones. Furthermore, in the presence of limited resources, optimisation of one function necessarily entails fragility of some other possibly unexpected one (Carlson & Doyle, 1999, 2002).

Dynamic resilience depends on the size and shape of each state's basin of attraction. These differ among individuals and, for a given individual, may change over time. The basins of attraction interact not only with external drives but also with both positive (autocatalytic) and negative (homeostatic) feedback forces, respectively reinforcing and damping the effects of perturbations. In the presence of weak positive feedback forces (e.g. between insomnia and mood), the system's response to changing conditions tends to be smooth whereas sufficiently strong self‐reinforcing autocatalytic feedback can cause the response curve to become folded so that there may be more than one stable state over a range of external conditions and a shift from a healthy to a pathological state may occur abruptly as a tipping point is reached. The distance from the tipping point defines the state's precariousness (Walker *et al*., 2004). Close to a tipping point, the system becomes less resilient and more likely to shift to a qualitatively different state.

For a healthy system, avoiding such points is therefore important. This involves finding early warning signs anticipating their occurrence. Two such signs are represented by critical slowing down, a generic dynamical phenomenon occurring in the vicinity of a bifurcation point where the system becomes increasingly slow when recovering from small perturbations back to its original state and fluctuations have characteristic properties, i.e. long‐term memory (Dai *et al*., 2012; Dai, Korolev & Gore, 2013; Liu *et al*., 2022). Slowing down of recovery is in general thought of as a generic risk marker (Rikkert *et al*., 2016), although inherently critical phenomena such as epileptic seizures may show no signs of such slowing down (Wilkat, Rings & Lehnertz, 2019).

## III. SYSTEMS WITH COMPLEX NETWORK STRUCTURE

Hitherto, we have thought of the brain as a generic high‐dimensional system with no particular internal structure, i.e. an essentially homogeneous and isotropic system. However, it is reasonable to assume that both brain anatomy and dynamics have some relevant non‐random structure in both time and space and that function may somehow be modulated by such a structure.

It has now become standard to think of brain anatomy, dynamics and function as a complex network (Bullmore & Sporns, 2009). A complex network is a strongly disordered heterogeneous structure (Dorogovtsev, Goltsev & Mendes, 2008) characterised by non‐trivial properties which do not feature in simpler ones, such as lattices or random networks (Albert & Barabási, 2002). Various fundamental questions need to be addressed. What does it mean to be a network? Is such a structure relevant to brain structure, dynamics and function? If so, how does the presence of complex network structure change the picture delineated so far and specifically, what role does network structure play in the way disease affects the brain?

### (1) What being a complex network means

Equipping the brain with a network representation has a number of important implications. First, it involves adding structure to the anatomical, dynamical and functional spaces, which can be taken advantage of to quantify brain anatomy and dynamics and ultimately brain function. At the most basic level, this involves distinguishing structures. Typical representations isomorphic to the anatomical space become difficult to compare when the physiological space is complex, e.g. non‐compact geometry due to noise and inter‐individual differences. A topological structure removes metric details and may in principle simplify the task (Stam *et al*., 2014). Deviations from network structure observed in the healthy brain may themselves have non‐trivial network structure (Zanin *et al*., 2014, 2018). Proximity relations in the neurophysiological space can also be defined using perturbation methods (Peters, 2016, 2018). Often, it is also important to quantify how far conditions are from each other both in the space made observable by signs and symptoms and in the corresponding neurophysiological space. The presence of a structure induces distances through which it is in principle possible to quantify various disease‐related aspects (Rossi, Torsello & Hancock, 2015; De Domenico & Biamonte, 2016). For instance, the connectivity matrix in real or phase space can be used as a metric tensor endowing the space with a Riemannian differential manifold structure (Amari & Nagaoka, 2007). The key point is that of translating all these anatomical or dynamical properties including distinguishability, proximity, neighbourhood, and accessibility into the way disease affects functionally relevant equivalent properties (Papo, 2019a). If disease implies a network structure modification with respect to the healthy brain, it is useful to identify and quantify such a change, either in a categorical or, ideally, in a quantitative way.

A genuine network structure involves a coarse‐graining wherein each portion of some observable space associated with the brain, e.g. the anatomical (real) space or the phase space of the dynamics, is identified with a discrete point, summarising a whole subsystem and the system is described through the relational structure of these subspaces. In analogy with the general way of defining brain function, the networks associated with bare dynamics are better called *dynamical*, while *functional* networks should be reserved for structures inducing partitions of the observable space through measures associated with the ability to perform some well‐specified task (Korhonen, Zanin & Papo, 2021).

If the brain has genuine complex network structure then disorder is relevant: its presence causes properties to deviate from those of homogeneous systems, and this should have an impact on its dynamics and ultimately on its function. For instance, heterogeneous degree distributions are responsible for novel types of phase transitions (Dorogovtsev *et al*., 2008). Likewise, we propose that a *network disease* is a condition where non‐trivial network structure relevant to brain function is damaged.

A complex network structure comes with some fundamental assumptions. First, network features are statistical properties arising from a great number of microscopic interactions, wherein single degrees of freedom (nodes or links) lose, at least *prima facie*, their identity. In this they profoundly differ from merely connected (usually much smaller) systems, where nodes and links are well identified, each having a specific functional role, and are therefore in no way interchangeable. In such a structure, connectivity serves a purely transport role, whereas in large‐scale networks it may have a more complex one. Second, the properties of complex networks emerge from connectivity, not from collectivity or mass action. Such properties may appear at scales below those of nodes but disappear as a result of coarse‐graining. Third, in the appropriate space in which the network is defined, at scales above those of nodes, the system may show genuine non‐locality, i.e. interaction‐induced emergence as opposed to bare anatomical connectivity.

Irrespective of the particular conceptual framework adopted to define disease, these are necessary ingredients not only for the networkness of brain function (Papo & Buldú, 2024) but also for a genuine network disease.

### (2) Brain network structure: space specification

Alongside the way disease and brain function are defined, a third factor determining the role network structure may play in disease is represented by the space in which network structure itself is defined.

**Fig. 2:**
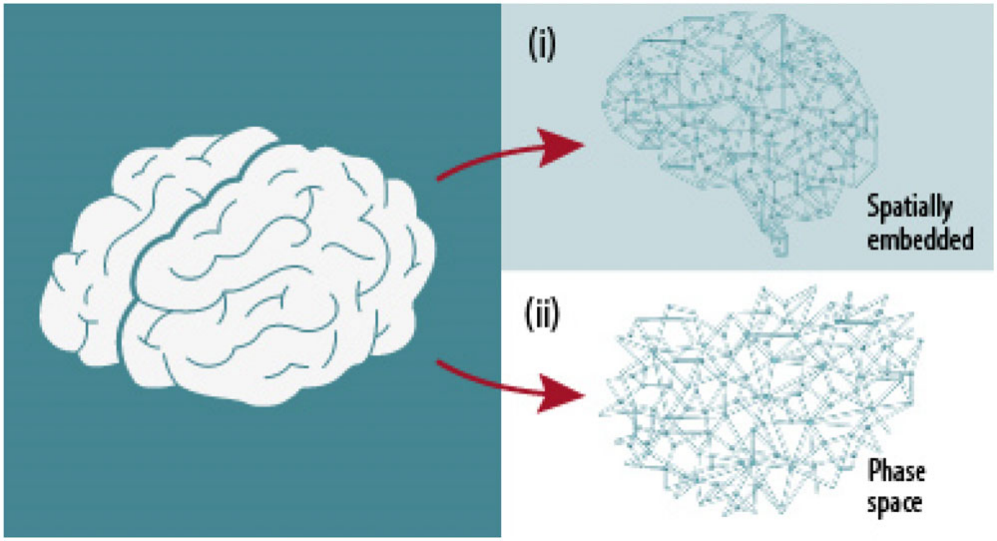
Endowing the brain with a network structure means mapping some aspect of its anatomy or dynamics on a set of nodes and links connecting them (Korhonen *et al*., 2021). This can be done in various ways. For instance, the network may be embedded in the brain's Euclidean space (*i*), in which case nodes represent brain regions and links either anatomical structures uniting them or some dynamical relation between activity at each node, or it may represent the brain's phase space (*ii*), in which case nodes may represent brain states and links transitions between them.

Various brain aspects may meaningfully be equipped with such a structure and also, importantly, some may not (e.g. purely feedforward, and ascending or descending systems). The anatomical structure, particularly for spatial scales at which nodes and links can respectively be mapped onto neurons and fibres uniting them, appears to be a particularly obvious candidate. Similar maps can apply to glial cells, proteins, or expressed genes (Thompson *et al*., 2020). However, other possibilities are also available, from real space (anatomy‐embedded) dynamics to the phase space of brain dynamics and the abstract space associated with pathologies and the relationships among them (Fig. 2). For instance, the relevant network for a given pathology may be defined on a gene or a protein space, where nodes are single expressed genes or proteins, and relationships, e.g. interaction between them or co‐expression, define links. Note that such a space may or may not have an explicit relationship with the underlying anatomical space. While anatomical networks are often modelled as acting upon a dynamic activity field, of which they constitute the spatial structure, the topology and geometry induced by brain activity merely shadowing such anatomical structure, dynamical networks are often considered in relation to brain function, which is typically identified with subspaces of dynamic connectivity's (spatiotemporal) structure.

Another key aspect is represented by the temporal and spatial scales at which a network structure can be defined. At fast timescales, the network structure induced by connectivity is approximately constant. For instance, at macroscopic spatial scales, brain anatomy may be thought of as essentially static at the typical timescales of a neuroimaging experimental session, while when considering local synaptic connectivity, the corresponding timescale may be of the order of milliseconds. However, even the slowest structure, i.e. the one associated with anatomy, can be endowed with its own dynamics at sufficiently long timescales, i.e. at developmental or experimental timescales of the order of seconds and beyond (Papo, 2013b). Considering the brain at developmental or evolutionary as opposed to experimental timescales allows understanding observed anatomy (and dynamical repertoire) as the result of a slow dynamics, the underlying forces of which can be modelled, and used as axes of appropriate morphospaces onto which particular network instances can be mapped (Corominas‐Murtra *et al*., 2013; Avena‐Koenigsberger *et al*., 2015). Conversely, brain dynamics may be endowed with network structure that can be thought of as static under appropriate conditions. A further important aspect relates to the spatial scales at which genuine non‐trivial network structure can be expected.

### (3) Structure–dynamics relations

Both at microscopic and at coarse‐grained, phenomenological scales the brain can be thought of as a networked dynamical system, where each node is associated with its own phase space of given structure (DeVille & Lerman, 2015). The main question will then be how network structure affects brain dynamics in disease. Note that the presence of a network structure incorporates not only spatial extension as already standard in dynamical disease models, but also a relational structure.

The presence of strong disorder associated with a complex network structure affects various dynamical properties of a system (Boccaletti *et al*., 2006; Porter & Gleeson, 2016). The brain can be thought of as a heterogeneously driven non‐linear networked system (Sreenivasan, Menon & Sihna, 2017). One important question is therefore how the presence of such a structure may affect its response to external fields. Network topology modulates the response of networked systems to applied fields and noise (Shinomoto & Kuramoto, 1986; Timme, 2007; DeDeo & Krakauer, 2012; Sonnenschein *et al*., 2013; Shi *et al*., 2023). Perturbation–response patterns of sets of given dynamical units may be consistent with classes of network topologies (Shandilya & Timme, 2011; Barzel, Liu & Barabási, 2015). More generally, insofar as the presence of disorder affects the system's scaling relations, transport coefficients and response function (Dorogovtsev *et al*., 2008), a complex network structure can be understood as a state of matter parametrisation, with symmetries intermediate between those of a solid crystal and those of a liquid (Papo, 2013a; Sun *et al*., 2024).

Network structure may also have profound effects on the system's dynamics. For instance, hierarchical modularity may lead to the emergence of metastability (Caprioglio & Berthouze, 2024). Furthermore, important dynamic phenomenological features require the presence of specific structure. For instance, properties such as multistability and sustained oscillations respectively require positive and negative feedback loops (Thomas, 1981; Zañudo & Albert, 2013). Moreover, in the presence of disorder, the dynamics associated with each node encode information about its topology (Timme, 2006; Burioni *et al*., 2014; Pernice *et al*., 2013; van Meegen, Kühn & Helias, 2021).

Non‐trivial network structure also affects processes unfolding on the network (Dorogovtsev *et al*., 2008) including synchronisation (Boccaletti *et al*., 2006; Arenas *et al*., 2008), information transport (Tadić, Rodgers & Thurner, 2007; Gallos *et al*., 2007; Barzel & Barabási, 2013; Mišić *et al*., 2015; Gollo, Roberts & Cocchi, 2017; Hens *et al*., 2019), and scaling properties (Moretti & Muñoz, 2013; Villegas, Moretti & Muñoz, 2014; Millán, Torres & Bianconi, 2018). For instance, quenched disorder of the anatomical network has been proposed as the structural mechanism through which brain dynamics becomes critical without fine parameter tuning (Moretti & Muñoz, 2013). The interplay between quenched anatomical and intrinsic frequency heterogeneities at various scales may give rise to a regime characterised by large non‐trivial spatio‐temporal fluctuations (Moretti & Muñoz, 2013) and metastability (Villegas *et al*., 2014) wherein the order parameter oscillates in a frequency‐dependent fashion with the coupling strength and is characterised by well‐separated hierarchically organised synchronisation domains of different frequencies (Villegas *et al*., 2014; Millán *et al*., 2018).

#### (a) The role of relative scales

The extent to which such a system's behaviour depends on the network structure on which it unfolds hinges on how dynamics at various spatial scales interact and this, in turn, on the relationship between the timescales at which they evolve. In the simplest case, one may consider the interactions between nodal and network dynamics (Do & Gross, 2012). In this framework, the connectivity matrix may itself be time dependent and may have its own dynamics and associated set of characteristic scales. If node dynamics is much slower than that of the network, what is studied is the evolution of non‐dynamic networks with static nodes. If, on the other hand, the timescale over which the network evolves is much larger than that of dynamical fluctuations, overall the system is treated as a static network, and time‐invariant coupling and network structure heterogeneity acts as quenched disorder (Ódor, 2008; Maslennikov & Nekorkin, 2017). When disorder has its own dynamics, network structure is considered to act as annealed disorder (Dorogovtsev *et al*., 2008). Thus, for instance, the anatomical network structure is often thought of as quenched at experimental timescales, whereas at longer (e.g. developmental or evolutionary) timescales, it can no longer be considered to be static and acts as annealed disorder. In addition to local dynamics, one could also consider the dynamics of its control parameters and more generally that of structure at all scales. In such a context, there may no longer be scale separation wherein a slow variable is only affected by the averaged state of the fast variables and the dynamical interplay between the timescales is relatively weak and, at various scales dynamics may potentially interact, a hallmark of *adaptive systems* (Gross & Blasius, 2008; Do & Gross, 2012; Maslennikov & Nekorkin, 2017; Berner *et al*., 2020).

Furthermore, the timescales of dynamical processes taking place on a network typically differ from both those of the nodes and of the network topology. Thus, in addition to the network structure, the system has two, typically separated scales – the slow one of the processes, and the fast one of nodes – each possibly being multiscale. The network structure on which processes unfold is often the anatomical one in the quasi‐static limit (Cabral *et al*., 2011, 2022), but it could also conceivably be the one induced by brain activity. Local dynamics, network structure, and dynamical process all have characteristic times of their own, so that ultimately macroscopic properties may result in a non‐trivial way from their mixing (Perra *et al*., 2012; Lambiotte, Rosvall & Scholtes, 2019; Papo, Goñi & Buldú, 2017). As we discuss in Section III.6, network neuroscience has been employed as a tool to classify brain diseases based on alterations in specific network parameters, some of which are shared across multiple disorders while others are unique to particular conditions.

### (4) From the network structure of function to the function of network structure

It is natural to think of observed network structure as functionally meaningful (Milo *et al*., 2002; Kashtan & Alon, 2005; Ashwin, Fadera & Postlethwaite, 2024; Bashan *et al*., 2012), and to assume that a particular wiring pattern determines the system's dynamics and function (Mastrogiuseppe & Ostojic, 2018; Ocker *et al*., 2017). For instance, it is intuitive that transport properties should depend on network structure at all scales (Mišić *et al*., 2015; Avena‐Koenigsberger *et al*., 2015; Villegas *et al*., 2022). The brain's computational properties may also crucially depend on network structure. For instance, memory encoding should depend on some property of the network supporting it (Susman, Brenner & Barak, 2019). Moreover, network structure can differ vastly in a function‐dependent way, and be designed to optimise a given function, e.g. information spreading, but not information processing or storage (Zylberberg *et al*., 2017).

If brain network properties are related to function in the healthy brain and altered in disease, dual questions are how network structure may enable the brain to carry out such function and bound it in the absence of disease (Petri *et al*., 2021), and how it may obstruct it in its presence. For instance, the brain can use the topological structure of neural networks in real space to perform computation (Jazayeri & Ostojic, 2021; Ostojic & Fusi, 2024; Ashwin *et al*., 2024), and such a structure may also explain the system's computational properties and its limits (Curto, 2017).

Following this line of reasoning, network‐related equivalence classes would also define functional equivalence classes. However, topological, geometric or combinatorial equivalence does not necessarily entail functional equivalence and whether such equivalence classes underlie brain function is unknown. In other words, these classes do not necessarily coincide with the structural aspects enabling the system to carry out its assigned function. For instance, whether isotopic networks are dynamically and functionally equivalent and, conversely, non‐isotopic networks inequivalent, is still poorly understood.

A further important aspect relates to scale dependence of network structure's functional significance. For instance, motifs (Milo *et al*., 2002) may have an information‐processing role at mesoscopic but not at larger scales: at system level, network structure may instead support transport rather than computation. Correspondingly, the role of network structure should also be scale dependent in pathology.

More generally, that a system has a given structure does not entail that such a structure is functional. Brain structures are organised to carry out different functions and therefore to satisfy multiple constraints. Different networks can give rise to similar function (Ganmor, Segev & Schneidman, 2015) while the same network can function differently depending on the context, both distinguishing features of degenerate systems (Edelman, 1987; Hennig, 2023). For instance, the same network motif can perform many different functions depending on functional requirements. Thus, on the one hand, function cannot always unequivocally be inferred from observed structure and, on the other hand, it is not straightforward to predict structure from function in neural networks (Biswas & Fitzgerald, 2022). Finally, identifying functional classes is not equivalent to identifying the structural aspects enabling the system to carry out its assigned function.

### (5) Network properties, invariants and equivalence classes

A complex network has combinatorial (Bollobás, 1986; Erickson, 2014), topological (Boccaletti *et al*., 2006), and geometric properties (Boguñá *et al*., 2021), as well as symmetries (Garlaschelli, Ruzzenenti & Basosi, 2010; Pecora *et al*., 2014; Dobson, Malnič & Marušič, 2022) which are encoded in the connectivity matrix. Understanding which of these properties turns out to be functionally relevant is central to the network neuroscience effort.

Network neuroscience often focuses on topology, both for real and phase space networks. Topology deals with properties that are invariant under continuous deformation without tearing or cutting, e.g. bending, twisting, and stretching, but does not consider other properties such as size or orientation. In such a framework, nodes and links are dimensionless, node location is disregarded, distances between nodes are not metric but defined as the minimal number of links in the connecting path, and the structural characteristics of brain networks are uniquely determined by the adjacency matrix. However, network layout and metric aspects may both play an important role in the brain (Wang & Kennedy, 2016; Henderson & Robinson, 2011, 2013, 2014; Roberts *et al*., 2016). For instance, the anatomical wiring cannot entirely be accounted for in terms of topological wiring rules and appears to stem at least partly from spatial embedding (Henderson & Robinson, 2011, 2013, 2014; Roberts *et al*., 2016). Physical wires cannot cross, and this imposes limitations on the system's structure (Bernal & Mason, 1960; Song, Wang & Makse, 2008; Cohen & Havlin, 2010; Dehmamy, Milanlouei & Barabási, 2018; Liu, Dehamy & Barabási, 2021), and mechanical properties qualitatively change with node and link density (Dehmamy *et al*., 2018). Moreover, the system's structure is determined not only by the connectivity matrix, but also by the network's spatial layout (Cohen & Havlin, 2010). Spatially embedded networks may have identical wiring but different geometrical layouts. Indeed, for a given adjacency matrix, a network can have an infinite number of layouts, differing in node spatial positions and wiring geometry. Likewise, topologically equivalent systems may be combinatorially different (Babson *et al*., 2006).

Network ensembles with given properties constitute equivalence classes. For instance, the layouts with identical wiring that can be mapped onto one another without link crossing or cutting define isotopy classes (Liu *et al*., 2021). This in turn allows defining equivalence relations between different systems, and rules to relate different points within or between systems, e.g. neighbourhood or proximity relations. On the other hand, the relationship between various aspects of network structure, e.g. the relationship between topology and geometry in brain networks, is still poorly understood.

### (6) A network‐based disease taxonomy

Brain pathologies differ along numerous characteristics, including aetiology, affected part of the system, associated cognitive dysfunction and behavioural correlates. Correspondingly, diseases differ in dynamics, spatial and temporal scales, predictability, and reversibility. But how does this translate into different roles of network structure implication in disease? Can we categorise brain diseases based on their underlying network structure and their role in pathology?

Considering networks in real space, disease may act on nodes, e.g. as the result of tumours or strokes, or by targeting connections, e.g. *via* demyelination and axonal injury. In either case, brain disease can induce both localised or widespread functional damage, either by propagating along neural connections to other areas, or by leading to anomalous connectivity (Raj & Powell, 2018). While various neurological and psychiatric disorders including epilepsy (Spencer, 2002; Lehnertz *et al*., 2009, 2017; Lehnertz, Bröhl & von Wrede, 2023; Kramer & Cash, 2012), stroke (Wang *et al*., 2010; Kuceyeski *et al*., 2014), traumatic brain injury (TBI) (Pandya, Kuceyeski & Raj, 2017), multiple sclerosis (Rocca *et al*., 2016), autism spectrum disorder (ASD) (Oberman, Rotenberg & Pascual‐Leone, 2014), schizophrenia (van den Heuvel *et al*., 2010; Fornito *et al*., 2012), and neurodegenerative diseases including AD (Lo *et al*., 2010; Buldú *et al*., 2011; de Haan *et al*., 2012), frontotemporal dementia (Kuceyeski, Zhang & Raj, 2012; Sedeño *et al*., 2017), and amyotrophic lateral sclerosis (Verstraete *et al*., 2011) are associated with changes in both global and local network organisation, from a system‐level perspective, one may distinguish non‐local disease from local disease with non‐local consequences. For instance, it is straightforward to think of TBI as a non‐local disease. Indeed, TBI typically causes diffuse axonal injury (Adams *et al*., 1980, 1989; Gentry, 1994; Sidaros *et al*., 2008; Hellyer *et al*., 2013), and disruption of long‐range fibres (Hulkower *et al*., 2013). These in turn promote secondary biochemical cascades hours to days after the initial injury (Greer, 2011), perturbing axonal transport processes, and ultimately resulting in the accumulation of products and alterations in neuronal homeostasis (Johnson, Stewart & Smith, 2013). On the other hand, Parkinson's disease (PD) appears as a focal disease with widespread motor and cognitive consequences. In PD, classic motor features are associated with the progressive neurodegeneration of nigrostriatal dopaminergic neurons in the substantia nigra pars compacta, or in some specific cortical region, as indicated by the focal onset of motor symptoms (Foffani & Obeso, 2018). Intermediate cases of various kinds are represented by schizophrenia, epilepsy, and AD. Schizophrenia has classically been characterised as a disconnection syndrome (Friston, 1998; Stephan, Friston & Frith, 2009; Schmidt *et al*., 2013; Hahamy, Behrmann & Malach, 2015; Vasa, Mostofsky & Ewen, 2016; Hilary & Grafman, 2017), but non‐trivial structure beyond connectivity has also been reported (van den Heuvel *et al*., 2010, 2013). Disease onset in AD may be relatively focal in the anatomical space, while its progression associated with characteristic functional impairment may be progressively more network‐like.

On the other hand, both focal and generalised seizures arise from the dynamics of a distributed, large‐scale aberrant network spanning lobes and hemispheres; seizure proneness in any part of the network can be affected by activity far away in the network, and electrical and behavioural phenomenology associated with seizures reflects the network as a whole (Spencer, 2002). Moreover, while a relationship between spatial–temporal seizure dynamics and global topological properties of the associated dynamical networks has been reported (Schindler *et al*., 2008; Kramer, Kolaczyk & Kirsch, 2008; Bialonski & Lehnertz, 2013; Burns *et al*., 2014), local network properties such as node centrality are not predictive of the seizure onset zone (Geier *et al*., 2015). Thus, epileptogenesis and ictogenesis can be thought of as an emergent property of aberrant large‐scale neuronal connectivity, and seizures as a network phenomenon (Lehnertz *et al*., 2009, 2017, 2023; Kramer & Cash, 2012). Interestingly, while epilepsy appears to be the prototype of diseases emerging as a result of global interactions, it also illustrates the possibility of local dynamic abnormality emerging from global abnormality, as accumulating evidence suggests that even focal epilepsy is associated with diffuse structural and dynamical abnormalities (Engel *et al*., 2013; Stam, 2014).

Diseases may also differ according to whether they directly affect the anatomical (e.g. AD, schizophrenia) or the dynamical (e.g. epilepsy) network structure, or a combination of both (e.g. AD and epilepsy). But this distinction may not always be clear‐cut. For instance, while seizure‐prone structures tend to be anatomically connected to damaged regions (Riederer *et al*., 2008; Bernhardt *et al*., 2011; Bernhardt, Bonilha & Gross, 2015; Raj, Kuceyeski & Weiner, 2012; Chiang & Haneef, 2014), they are in essence of a dynamical nature.

A further way to classify brain diseases is to consider the relationship between anatomy, dynamics, and function. For instance, in epilepsy, the functional space is identified with the dynamical one, as the former becomes trivial during seizures. This differs from other psychiatric diseases, in which dynamics and function are qualitatively different spaces, and non‐trivial mapping between them needs to be defined. Finally, the role of network structure may differ across diseases: in some cases, e.g. in tumours or in epilepsy, it may be a structure emerging from disease, while in other cases the role of network structure is that of pushing brain dynamics to pathological regimes.

## IV. DISEASE AS HEALTHY NETWORK STRUCTURE ALTERATION

Whether induced by internal dysfunction or by external factors such as pathogens or trauma, disease may be associated with damage to an entire system or to some of its parts. The notion that stable groups of symptoms are associated with lesions of well‐defined anatomical regions dates back to Morgagni (1682–1771) and underlies the foundations of anatomical pathology.

Equipping the brain with a network structure adds three aspects to this foundational framework. First, if some network property has functional meaning, it is natural to suppose that its modifications should be associated with pathology, and conversely that at least some neurological and psychiatric pathologies should be associated with abnormalities in network structure. Second, the consequences of damage are non‐local, i.e. the structure–function map should be complex, irrespective of whether the lesion is itself local or not. Third, brain dysfunction may be thought of as a distance from a functional structure and quantified in terms of the magnitude of such distance.

Two fundamental questions should be addressed. Does disease affect the network structure and if so, what aspects of brain physiology (e.g. neural population or vascular system) and network structure are affected by disease? How *can* disease affect network structure?

### (1) Connectome alterations in brain pathology

Both in traumatic brain injury and in neurodegenerative diseases, it is natural to think that disease acts on the brain's network structure, possibly altering its geometry and topology. Brain injury is characterised by primary damage which induces immediate degeneration and cell death in regions proximal to the insult, but also secondary damage triggering downstream events that can extend to remote regions spared by primary damage (Park, Velumian & Fehlings, 2004). Secondary damage is typically proportional to the extension and duration of primary damage (Block, Dihne & Loos, 2005) but of much longer duration. Remote damage can result from axonal damage or from transneuronal degeneration, which can involve neurons losing their projection target or their input (Viscomi & Molinari, 2014). Axonal damage gives rise to a number of local and nonlocal phenomena including Wallerian and Wallerian‐like degeneration, ultimately resulting in disconnection and loss of signalling (Seeley *et al*., 2009), which can conceivably translate into changes in brain network geometry and topology.

Charting the brain overall relational structure is the main goal of recent collective connectome projects (Alivisatos *et al*., 2012, 2013; Larson‐Prior *et al*., 2013; Van Essen *et al*., 2012, 2013; Allen *et al*., 2014; Okano, Miyawaki & Kasai, 2015; Glasser *et al*., 2016; Amunts *et al*., 2016; Miller *et al*., 2016; Poo *et al*., 2016; Howell *et al*., 2019), as well as of functional cartography (Mattar *et al*., 2015; Jirsa *et al*., 2010) and discovery science (Biswal *et al*., 2010). A similar representation can be associated with gene (Bota, Dong & Swanson, 2003; Patania *et al*., 2019) or neurotransmitter expression (Hansen *et al*., 2022). While the connectome represents static connectivity between brain areas, the chronnectome involves a temporal dimension, describing brain dynamics as a set of reoccurring, temporal connectivity patterns (Calhoun *et al*., 2014; Iraji *et al*., 2019).

In the same way that a connectome is intended as a complete anatomical, dynamical and even genuinely functional network structure of the healthy brain, pathoconnectomics should constitute a map of abnormal brain networks (Rubinov & Bullmore, 2013), the main underlying idea being that the connectome could constitute a biomarker for disease (Kaiser, 2013), and therefore that pathoconnectomics is in fact genuine functional structure.

Already at a purely dynamical level, various fundamental reasons render the connectome induced by dynamics, usually termed *functional connectome*, inherently more complicated to define. First, defining links in dynamical networks is far less straightforward than in the anatomical case as for instance no connectivity metric is explicitly based on neurophysiology. Each of the available metrics is predicated upon assumptions on the way brain units are related to each other and, even more fundamentally, on the functional meaning of such relation. Second, the possibility of such interactions occurring on multiple temporal scales places an additional challenge on the detection of interactions and the timescales of the associated phenomena can be rather non‐trivial (Papo, 2013b, 2015). Mapping the dynamical space into the functional one adds a further complexity layer, important reasons being the presence of neutral networks in the physiological space and highly degenerate circuits. In a degenerate system, lesions are not necessarily associated with functional deficit, and this makes it difficult to establish whether a brain region is necessary for a given cognitive process (Price & Friston, 2002). To determine whether network structure damage underlies the functional disruption associated with disease it is therefore necessary first to identify what aspect of brain structure is functionally relevant and then to understand the functional meaning of structure from which the structure–function relationship can ultimately be deduced.

### (2) Structural consequences of disease

Connectomes are *per se* bare (anatomical or dynamical) connectivity charts. The role of connectivity in brain function (Kozma & Freeman, 2016) and the impact of its increase or decrease in neurological and psychiatric conditions (Geschwind, 1965; Friston, 1998; Geschwind & Levitt, 2007; Casanova & Trippe, 2009; Stephan *et al*., 2009; Terry, Benjamin & Richardson, 2012; Schmidt *et al*., 2013; Oberman *et al*., 2014; Hahamy *et al*., 2015; Vasa *et al*., 2016; Hilary & Grafman, 2017) has convincingly been documented.

Evidence for network diseases in analogy with dysconnection syndromes (Geschwind, 1965; Weinberger, 1993; Friston & Frith, 1995; Friston, 1998; Stephan *et al*., 2009; Dineen *et al*., 2009; Drzezga *et al*., 2011; Friston *et al*., 2016) would go a long way towards demonstrating the genuine network‐like nature of brain function. Arguably the first step in this direction consists in documenting changes in non‐trivial anatomical and dynamical network structure in neurological and psychiatric conditions [see Stam (2014) and Buldú *et al*. (2024) for reviews].

One important question is whether the functional impairment associated with dysconnection syndromes, e.g. AD or schizophrenia, is also associated with changes in non‐trivial higher‐order network structure induced by variations in connectivity. For instance, alterations of steady‐state structure of brain dynamics in AD have been shown to induce loss of non‐trivial network structure and a shift towards increasingly random structure (Buldú *et al*., 2011), consistent with a vision of disease as loss of complexity rather than regularity (West, 2010). On the other hand, schizophrenia has been associated with a reduction in white matter connectivity selectively affecting the so‐called *rich club*, i.e. the pathways connecting high‐degree nodes (van den Heuvel *et al*., 2010, 2013). Moreover, high‐order non‐linear interactions in multi‐task networks have been proposed as indicators of aberrant brain function in schizophrenia (Plis *et al*., 2014). Change within the network may be non‐local. For instance, reconfiguration of the epileptic brain network potentially affects any network constituent and their dynamical connections (Lehnertz *et al*., 2017, 2023; Rings, von Wrede & Lehnertz, 2019b).

On a related level, a further important issue is the specificity and sensitivity of network structure to given pathologies. While there may be differences in the network properties of anatomical and dynamical networks between a given disease and the healthy brain, this does not *per se* guarantee that the former is a genuine network disease, even when differences between populations, e.g. between fronto‐temporal dementia and healthy controls (Sedeño *et al*., 2017) are only found in terms of network properties.

While the emphasis of connectomics is in general on the bare connectivity and topology of brain networks, brain network combinatorics and geometry can also be affected in disease in both real and phase space. For instance, both physicality (Pósfai *et al*., 2024) and topology–geometry interactions presumably play an important role in pathologies such as tumours. It is particularly important to understand not only what role that such factors play on the network structure and ultimately on brain dynamics and function, but also when this may start being important. Furthermore, pathology can affect anatomical (Simhal *et al*., 2020) and dynamical network curvature.

### (3) From structure of the dynamics to the dynamics of structure

It has long been recognised that at both neurophysiological and behavioural levels at least some diseases have an inherently dynamical nature and undergo bifurcations, i.e. qualitative changes, for some values of parameters controlling the dynamics (Mackey & Glass, 1977; Glass & Mackey, 1979, 1988; Glass, 2015). However, what role is played by network structure is still not totally clear.

In essence, a dynamical disease with a network support can display bifurcations either of the dynamics or of some network property, e.g. topological phase transitions. These correspond to two aspects of the relationship of network structure to dynamics, i.e. the network structure induced by the spatiotemporal organisation of the dynamics and the space within which lives the dynamics induced by the anatomical network. Each of these aspects sheds light onto some role of network structure in brain disease.

#### (a) Dynamical consequences of anatomical structure damage

Above, we reviewed possible disease‐induced emergent network structure. However, disease may also involve network‐induced changes in dynamics. At the most basic level, insofar as complex network structure can be understood as a state of matter (Papo, 2013a; Sun *et al*., 2024), disease can change the system's spontaneous fluctuations and generic response to external fields (Demetrius, 2013). Moreover, disease may act by pushing brain dynamics away from criticality. Indeed, global scaling properties have been shown to be altered in various pathologies (Oberman *et al*., 2014; Bruining *et al*., 2020; Liang, Yang & Zhou, 2024). But what role does the network structure play in this action of disease on dynamics?

Disease may act on dynamics through its action on the anatomical network structure, which may force criticality without fine parameter tuning in the healthy brain (Moretti & Muñoz, 2013). Non‐trivial anatomical network structure and its modifications induced by disease could act as topological defects (Mermin, 1979; Ódor, 2008; Nishimori & Ortiz, 2010; Bowick *et al*., 2022; Shankar *et al*., 2022). Such a structure may be relevant to a number of pathologies. For instance, tumour formation can be associated with collective flocking motion through a medium filled with defects.

Disease may also act directly on the dynamical structure. At the most basic level, this may occur *via* aberrant dynamic connectivity (Oberman *et al*., 2014). Non‐trivial scaling in networked systems has been proposed to arise from the balance between excitation and inhibition (van Vreeswijk & Sompolinsky, 1996). Thus, the main pathophysiological mechanism in diseases such as ASD and schizophrenia may be altered intracortical inhibition within neural microcircuitry (Eichler & Meier, 2008; Yizhar *et al*., 2011). For instance, reduced inhibition in ASD (Rubenstein & Merzenich, 2003; Markram & Markram, 2010; Vattikuti & Chow, 2010) may result in an increased excitation/inhibition ratio (Rubenstein & Merzenich, 2003; Nelson & Valakh, 2015; Dickinson, Jones & Milne, 2016) ultimately modifying the system's macroscopic scaling properties (Liang *et al*., 2024). Brain disease may induce dynamical changes by disrupting non‐trivial structural properties at macroscopic scales. For instance, the loss of hierarchical modularity in pathologies such as AD or schizophrenia may impair brain metastability (Caprioglio & Berthouze, 2024).

#### (b) Structure dynamics in disease

If disease is associated with changes in network structure, it is important to understand the nature of such changes. In this sense, the difference between healthy activity and pathology may be comparable to dynamical and functional switching in the healthy brain (Papo & Buldú, 2024; D. Papo & J.M. Buldú, in preparation). Likewise, disease is itself often inherently dynamic (Torres *et al*., 2016; Fülop *et al*., 2020; Liu *et al*., 2022), with varying symptoms and underlying neurophysiology, and this may be mirrored by structural dynamics. One fundamental question involves determining whether such network structure changes are quantitative or qualitative. Various scenarios are possible in principle.

First, the transition to pathology may not be associated with any observable topological change, even in the presence of qualitative behavioural changes. For instance, generalised or focal seizures may arise as a consequence of subtle changes in network structure (Terry *et al*., 2012). A second possibility is that disease is associated with genuine topological phase transitions associated with singularities in global network structure properties in the anatomical or dynamical network structure. Such transitions can emerge as the level of noise associated with rewiring is varied (Derényi *et al*., 2004; Derényi, Palla & Vicsek, 2005; Palla *et al*., 2004). Metastable‐state transitions with topological changes in the minimal‐spanning‐tree of the network induced by cross‐correlations in electrical brain activity have been reported (Bianco *et al*., 2007). However, whether network topology in the real space acts as a control parameter enforcing brain dynamics bifurcations at some scale is insufficiently understood. A third possibility is that disease is associated with transitions in the phase space of the dynamics. Under some conditions, topological changes undergone by the level subsets of some configuration property of the manifold are related to singularities in microcanonical entropy (Pettini, 2007; Kastner, 2008; Santos *et al*., 2014; Casetti, Pettini & Cohen, 2000). In dynamical brain networks, this can occur for dynamic coupling levels emerging as a connectivity threshold is varied (Santos *et al*., 2019; de Amorim Filho, Moreira & Santos, 2022). This topological change is associated with singularities of the Euler characteristic, an integer‐valued topological invariant describing the system's associated structure (Matsumoto, 2002; Ghrist, 2014). However, while in systems with short‐range interactions topological changes in the configurational space constitute a necessary (although not sufficient) condition for phase transitions to occur (Casetti *et al*., 2000; Franzosi & Pettini, 2004; Pettini, 2007; Kastner, 2008), for systems with long‐range interaction potential, topological properties of the potential energy may not change greatly at a second‐order phase transition (Campa, Dauxois & Ruffo, 2009). Moreover, topological changes may be numerous even away from a phase transition (Kastner & Schnetz, 2008). A necessary criterion for the occurrence of a thermodynamic phase transition relates to the curvature at the saddle points of the potential (Kastner & Schnetz, 2008). Disease could also be associated with anomalous scale‐dependent topological cross‐overs (Rozenfeld, Song & Makse, 2010; DeDeo & Krakauer, 2012; Villegas *et al*., 2022). Indeed, healthy brain activity may be characterised by hierarchical organisation into modules with large‐world self‐similar properties at macroscopic scales, and the addition of only a few weak links can turn the system into a non‐fractal small‐world network (Gallos, Makse & Sigman, 2012), but how this may possibly change as a result of brain pathology has not been directly addressed.

A further question requires understanding which of the various aspects of network structure (topology, geometry, combinatorics) may be affected in disease. For instance, disease may affect brain network geometry without affecting topology, as for instance in tumour invasion. Likewise, in damage to memory‐related function, e.g. in the hippocampus, combinatorial transitions may induce functional impairment without changing topology.

### (4) Role of network structure in the functional consequences of disease

Pathology typically affects the brain's ability to carry out its functions, by reducing its efficiency and reliability (Schwarze *et al*., 2024), sometimes leading to complete functional breakdown. Disease‐related structural changes may lead not only to dynamical and functional inefficiency but also to thermodynamical dysfunction (Zhang & Raichle, 2010), although the relationship between network structure and thermodynamics is still poorly understood. But does disease affect brain function by altering its network structure? For instance, does impairment in information spreading, encoding, and processing proceed from structure damage? Can the loss of efficiency in the way the brain carries out the function it is supposed to perform and in the cost‐efficiency trade‐offs that it makes be quantified in terms of network properties? Conversely, can functional changes force network structural changes?

#### (a) Network scales of disease action

When considering a neural population in the real anatomically embedded space, the first question is to identify the network level at which disease may act. Functional damage may occur at scales at which there is no network‐like activity; conversely, at scales for which such activity is present, damage may be renormalised within single nodes. Thus, at the scales at which network structure may be functionally relevant, disease‐related perturbations may act not directly upon the network topology, but upon nodal dynamics. For instance, in epileptic seizures macroscale dynamics may be driven by microscale neuronal activity (Burrows *et al*., 2023). This may change the system's dynamics without modifying anatomical or even dynamical network structure (or function).

While the meaning of nodes and links is in general scale dependent, it has been proposed that at the system level, connectivity may carry information about disease severity, while local node parameters may be related to aetiology and prognosis (Zonca *et al*., 2024).

#### (b) Network structure and activity propagation in disease

One fundamental way in which disease may affect brain function efficiency is by changing the way activity propagates across the space. Indeed, network efficiency is in general framed in terms of propagation rather than of the ability of a given networked system to perform some task. In turn, how activity spreads through a network is often characterised not directly in terms of the propagation process, whose nature is in general unknown, but indirectly in terms of the substrate topology visited by that process (Latora & Marchiori, 2001).

How perturbations propagate through networks, impact and disrupt their functions may depend not only on the type and location of the perturbation, but also on the interplay between topological properties, interaction dynamics, and self‐dynamics (Avena‐Koenigsberger, Misic & Sporns, 2018; Hens *et al*., 2019; Bao *et al*., 2022).

While the most obvious way in which disease can affect activity propagation is through outright disconnection, network properties ranging from local ones such as node degree, to mesoscale structure such as motifs and hubs can shape global communication and facilitate integrative function (Mišić *et al*., 2015), determining the scaling regimes and propagation properties (Bao *et al*., 2022). Importantly, damage may also hinder activity propagation across scales, even in the absence of anatomical damage (Ghavasieh, Bertagnolli & De Domenico, 2023).

#### (c) Network structure and information transport and processing in disease

Brain pathology can induce a range of functional deficits with characteristic behavioural correlates. While these deficits may often be thought of as the result of changes in brain transport properties, they may also emerge in alternative ways.

At the most basic level, it is important to understand the role of network structure in the way brain responses to external stimuli may vary as a result of disease. When considering the relationship between network structure and response function of a given system, two dual aspects should be considered. So far, we have considered network structure changes associated with given pathologies. However, a second factor is the way network topology affects the response to external fields. The brain responds to external stimuli by computing an implicit model of the environmental variables through transient patterns of dynamics (Tsuda, 2001). As it performs computation the brain faces a trade‐off between sensitivity and reliability. On the one hand transient dynamics must be input specific. On the other hand, computations must be reproducible, robust against noise and variations in initial conditions, and easily decoded. Input‐specific yet structurally reliable transient dynamics can be achieved with heteroclinic dynamics (Rabinovich, Huerta & Laurent, 2008; Ashwin *et al*., 2024). In a high‐dimensional networked system, this can be achieved with asymmetric inhibitory connections (Nowotny & Rabinovich, 2007). This type of connectivity can coordinate the sequential activity of neuronal populations and stabilise heteroclinic channels (Rabinovich *et al*., 2008). While higher‐network structure also plays a role in the response function (Sreenivasan *et al*., 2017), the role of non‐trivial network structure in the response function alterations in disease, including the scale at which this may happen and the properties through which disease can act, is still poorly understood.

One intuitive way to understand the functional role of network structure in disease consists of interpreting structure in energetic and evolutionary terms. For instance, the energetic cost associated with the rich club structure points to a functionally advantageous structure, and rich club hypoconnectivity in schizophrenia likely affects the system's communication capacity. However, it is not straightforward to explain altered brain dynamics and behavioural phenomenology in schizophrenia in these terms. A functionally more interpretable aspect may be represented by the breakdown of modularity (David, 1994; Alexander‐Bloch *et al*., 2010; Godwin, Barry & Marois, 2015). For both anatomical and dynamical networks, a modular structure is characterised in real space by the presence of groups of nodes with dense or strong intrinsic but sparse or weak extrinsic connectivity. Modularity is associated with structure at various, possibly hierarchically organised (Pathak, Menon & Sinha, 2024) spatial (Meunier, Lambiotte & Bullmore, 2010) and corresponding timescales, naturally introducing separation between intra‐ and intermodular scales. Correspondingly, a modular structure may constitute a functionally efficient architecture, provided it realises an optimal balance between local segregated and global integrated activity modes (Tononi, Sporns & Edelman, 1994; Tononi & Edelman, 2000). Functional impairment associated with dysmodularity may stem from either intra‐ or inter‐module dysconnectivity. In both cases, this can emerge from hypo‐ and hyperconnectivity. From the developmental perspective relevant to schizophrenia or ASD, this may respectively correspond to excessive and insufficient cortical pruning. For instance, ASD has been characterised both as a developmental hypo‐ (Geschwind & Levitt, 2007), and hyperconnection syndrome (Casanova & Trippe, 2009). However, while emerging deficits in schizophrenia may result from excessive cortical pruning in a way similar to what may happen in a neurodegenerative disease, decreased global pruning seems a more plausible model. In the presence of increased anatomical and dynamical connectivity induced by reduced pruning, modules can become more interconnected, with shortcuts among modules allowing changes to propagate. This may explain the impairment of supramodular functions characteristic of schizophrenia, including deficits in perceptual filtering (McGhie & Chapman, 1961), vulnerability to overload, proneness to perceptual interference (Liddle & Morris, 1991), overflow of linguistic analysis into perceptual systems, together resulting in thought insertion and auditory hallucinations and a tendency to spurious associations (Frith, 1992). The possible presence of hierarchical modularity adds further accessible ways through which pathology may arise from network structure perturbations. For instance, schizophrenia has been shown to be associated with enhanced global hierarchical organisation at rest (Acero‐Pousa *et al*., 2024). However, while a structural change is associated with negative symptoms including apathy and formal thought, it is unclear how specifically the former can be used to explain the functional aspect of the latter.

Conversely, disease‐related network structure can emerge as the result of functional changes associated with disease. The role of network structure in disease‐related information‐processing impairment can be understood by considering topological network features such as heterogeneity and modularity as emergent properties of functional trade‐offs (Zylberberg *et al*., 2017; Ghavasieh & De Domenico, 2024). For instance, it has recently been proposed that mesoscale structure can emerge from a trade‐off between information exchange and response diversity over a wide range of timescales (Ghavasieh & De Domenico, 2024). Thus, disease‐related changes in network structure may point to changes in the balance between information transport and processing (Zylberberg *et al*., 2017; Ghavasieh & De Domenico, 2024).

## V. NETWORK STRUCTURE OF DISEASE AND BRAIN–DISEASE INTERACTIONS

In some instances, it may be natural to think of disease as a separate system. As a consequence, disease itself may have its own structure. Such a structure can be understood as a complex network at three different levels: in the space of diseases, in the behavioural space of observable behavioural symptoms, and in the underlying space of neurophysiological processes from which these symptoms arise.

The idea that the space of pathologies has a certain order, upon which the discipline of nosography is predicated, dates back to Thomas Sydenham (1624–1689). It is then natural to chart brain disorders and the relations among them (Cristino *et al*., 2014; Fornito, Zalesky & Breakspear, 2015; Halu *et al*., 2019; Guloksuz, Pries & Van Os, 2017). The space of human diseases and disorders may be endowed with a network structure induced by genetic or protein interactions (Goh *et al*., 2007). Moreover, diseases, particularly within a geographical area, may induce a space where single diseases may interact in various ways (e.g. antagonistic or synergistic), a notion known as *pathocenosis* (Nicolle, 1933; Grmek, 1969; Gonzalez *et al*., 2010). In graph theoretical terms, such a space is naturally thought of as a network‐of‐networks (Gao *et al*., 2011; Kenett, Perc & Boccaletti, 2015; Kiani, Gomez‐Cabrero & Bianconi, 2021). Within the functional space made observable by clinical phenotypic manifestations, brain disorders can be thought of as systems of subject‐specific causally connected symptoms rather than as effects of a latent disorder (Borsboom & Cramer, 2013; Zhou *et al*., 2014; Guloksuz *et al*., 2017; Epskamp, Borsboom & Fried, 2018; Tosi *et al*., 2024). Diseases share not only signs and symptoms, but also underlying physiology, including genes and proteins, as well as therapies to treat them. Finally, in some cases, for instance involving invading masses and tumours, it is also natural to ascribe to disease a structure of its own that is distinguishable from that of the healthy brain (Mandal *et al*., 2024).

If disease is thought of as a separate system, it is important to understand how it interacts with the brain. There are various ways in which the interaction between networked systems can be understood. Disease can for instance be thought of as a process unfolding on network structure, which can be static or have its own timescales. Moreover, it is straightforward to conceive of the brain–disease interaction as competitive, although other forms of interaction are in principle possible. Insofar as both brain and disease may possess a network structure, the interaction can ultimately be understood as network competition. However, the interaction need not always be of a competitive nature and the two systems may sometimes better be thought of as coevolving.

### (1) Disease as a process on a network

Above, we have considered how disease may be associated with changes in network structure. However, various pathologies, e.g. neurodegenerative diseases, may induce not only dynamics *of* networks but also dynamics *on* them, with some diseases potentially presenting both kinds of effects (Raj & Powell, 2018). Disease can then be thought of as a process (or modification of a process) unfolding on the structure, the timescales of which are typically different from those of the dynamics of its constituent parts. The interplay between dynamical processes and the structure on which they unfold induces novel scales. For instance, dynamical processes induce specific latent geometries, which influence how activity or information may spread across the system (Brockmann & Helbing, 2013; De Domenico, 2017; Barzon *et al*., 2024).

#### (a) The role of network structure in disease spreading

Various brain pathologies present a distinctive disease progression pattern, with stereotypical neuronal events and corresponding characteristic temporal and spatial scales. For instance, neurodegenerative disorders display highly stereotyped patterns of disease progression, from the entorhinal cortex and hippocampus to the temporal, parietal, and eventually frontal regions (Buckner *et al*., 2005), whose progression follows fibre pathways rather than proximity (Seeley *et al*., 2009), characterised by atrophy, tau and beta amyloid pathology, and metabolic load.

Despite fibre tract damage, and evidence that amyloid progression stages are associated with network structure changes between default‐mode network brain areas (Pereira *et al*., 2018), at least some macroscopic aspects of the anatomical network topology in AD is preserved (Powell *et al*., 2018). Neuropathological evidence suggests that dementias, whose onset is associated with brain atrophy as well as misfolded beta amyloid and tau protein accumulation in the grey matter, may be characterised by spreading along spared white matter tracts through prion‐like trans‐synaptic transmission of misfolded tau and beta amyloid (Brundin, Melki & Kopito, 2010; Raj *et al*., 2012; Clavaguera *et al*., 2015). To understand how this process interacts with the network structure it unfolds on, it is necessary to characterise the process itself, at both neuronal and dynamical level, e.g. *via* passive diffusion, advection, active axonal transport, or other distance‐independent processes (Braak & Braak, 1991; Braak *et al*., 2003; Iturria‐Medina *et al*., 2014, 2017; Raj & Powell, 2018; Peraza *et al*., 2019; Del Tredici & Braak, 2020; Pandya & Patani 2021; Millán *et al*., 2022; Rapisardi, Kryven & Arenas, 2022). In the former case, misfolded and aggregated proteins may spread through distance‐dependent spatial gradient‐driven processes (Warren *et al*., 2013), and the underlying protein transmission process can be modelled as a geometry‐driven heat equation (Raj *et al*., 2012). In the latter case, overall fibre density, corresponding to connection strength at coarse‐grained scales, should be more important than distance‐dependent spatial diffusion. The atrophy patterns of protein transmission in various dementias were predicted by the Laplacian eigenmodes associated with the dynamics predicted by connectivity‐driven spreading models (Stumpf & Krakauer, 2000; Matthäus, 2006), consistent with the notion that regional vulnerability to AD is predicted more by network connectivity than by the expression of AD‐related genes and the molecular factors they promote.

A similar use of the network has been proposed to apply to disease progression in schizophrenia. While schizophrenia is characterised by progressive waves of tissue loss (van Haren *et al*., 2008), with atrophy and cortical thinning (DeLisi *et al*., 1997; Kubota *et al*., 2013), dynamic interregional connectivity changes affect the way activity spreads on the anatomical network (Abdelnour, Vos & Raj, 2014) eventually disrupting temporal communication between higher‐order brain regions (Hunt *et al*., 2017). On the other hand, while atrophy dynamics in temporal lobe epilepsy (Bonilha *et al*., 2003; Bernhardt *et al*., 2009; Mueller *et al*., 2009) could in principle result from epileptogenic hyperactivity propagation (Sutula, Hagen & Pitkänen, 2003; Riederer *et al*., 2008) resulting from excitotoxicity (Mehta *et al*., 2013), it is likelier the result of network dynamics associated with progressive deafferentation, followed by progressive neuronal loss in connected remote regions (Abdelnour *et al*., 2014; Abdelnour, Mueller & Raj, 2015).

#### (b) The role of network structure in pathological processes at fast scales

In some pathologies, e.g. epilepsy or multiple sclerosis, in addition to the disease progression timescale, processes may also unfold at the shorter timescales of disease episodes. One important question is whether seizure‐related activity follows the same path as physiological activity (Zaveri *et al*., 2020). While seizure initiation and propagation have been shown in association with novel local pathways (Bragin, Wilson & Engel, 2000; Scharfman *et al*., 2003), and large‐scale anatomical network reorganisation in pharmacoresistant epilepsy patients is common although not ubiquitous (Tellez‐Zenteno *et al*., 2010), seizure propagation has also been shown to use the same pathways as healthy activity in rodents (Rossi *et al*., 2017). Thus, seizure spreading may depend on the existing network topology and may not be related to atrophy (Lehnertz *et al*., 2023).

At the single‐episode scale, spreading is not the only way in which the network can be used by a neural process. For instance, insofar as epilepsy can be understood not only at the spread of hyperexcitability but also as a synchronisation process on the anatomic network, the network structure may play a role in the way the system synchronises, ultimately giving rise to seizure phenomenology (Dyhrfjeld‐Johnsen *et al*., 2007; Chavez *et al*., 2010; Lehnertz *et al*., 2023).

#### (c) Universal mechanisms in brain disease dynamics

Brain diseases vastly differ from each other not only in aetiology and dynamical and functional phenomenology but also in their spatial characteristics. In spite of such profound differences, there may also exist some universal dynamical traits or mechanisms shared by at least groups of pathologies. For instance, brain injury may be characterised by the spreading along the topological structure of its anatomical connectivity of sleep‐like cortical dynamics, resulting from disease‐induced local changes in ascending input and lateral excitation and the consequent excitation/inhibition imbalance (Massimini *et al*., 2024). Likewise, while neurodegenerative diseases feature distinct patterns of selective vulnerability of some classes of neurons, characterised by neuronal loss and protein accumulation, a phenomenon known as pathoklisis (Eser *et al*., 2018), dementias may share a common progression mechanism irrespective of aetiology, region‐specific neuropathy (Braak *et al*., 2000; Pandya & Patani, 2021), or selective vulnerability within dissociated functional networks (Seeley *et al*., 2009).

### (2) Brain–disease interactions

Once brain and disease are thought of as separate networked systems it is natural to assume that they may interact. The interaction between two networked subsystems can have various profound effects. For instance, the interaction of a given subgraph with other nodes in the network affects whether that subgraph corresponds to a fixed‐point support (Morrison & Curto, 2019).

What the effects of interactions between networks may be depends on the way they interact. Such a relationship may be understood in terms of interdependence, competition, or even cooperation between network structures (Aguirre, Papo & Buldú, 2013; Mišić *et al*., 2015; Danziger *et al*., 2019) or coevolution. For instance, interdependence and competition may respectively correspond to positive and negative correlations in the coupling of a system's local connectivity with the other system's local order (Danziger *et al*., 2019). Whether this simply has dynamical significance or a functional one as well depends on how local order reflects the instantaneous local functionality.

If disease can effectively be considered as a separate networked system interacting with the brain network, one potentially important factor is the way these two networks make contact with each other. This may have two potentially interesting aspects. First, connector nodes may play an important role in the brain–disease interaction (Aguirre *et al*., 2013, 2014; Buldú *et al*., 2016). More generally, the interface may itself possess non‐trivial network structure, so that disease stages may be quantified in terms of the interface structure and its dynamics.

Another important but still poorly understood question is represented by the quantity these systems may compete for and how this can be expressed in network terms, some form of centrality representing a possible candidate (Aguirre *et al*., 2013). For example, the activity of the two brain hemispheres has been described as a competition process for gaining centrality in the functional network (Martínez *et al*., 2018a). An analysis of the eigenvector centrality across different frequency bands and conditions showed that the hemispheres generally maintain a functional balance, with no significant centrality dominance of one hemisphere over the other. However, when networks were made sparser, this balance shifted, leading to scenarios where one hemisphere could accumulate disproportionately higher centrality.

## VI. CONFRONTING DISEASE: THE ROLE OF NETWORK STRUCTURE

One important question is whether subjects' proneness and response to and recovery from disease is related to the system's network properties prior to its inception. Likewise, the structure of, or induced by, disease may create dynamical traps, which the system cannot easily escape from (Scheffer *et al*., 2024). The underlying idea is that network structure may not only act as a functional substrate but also as a mechanism of resistance to disease (Fornito *et al*., 2015). In this sense, the resilience of the anatomical network, in many cases sustained by a high degree of degeneracy, is crucial to maintain the brain within a healthy behaviour (Tononi, Sporns & Edelman, 1999; Zamora‐López, Zhou & Kurths, 2010) Multiple levels of degeneracy have been reported in the human brain, from microcircuits to large‐scale connectomics (Marder & Goaillard, 2006) and it has been suggested as one of the key elements behind cognitive reserve and, consequently, healthy ageing (Barulli & Stern, 2013).

The possible role of network structure in resilience can be understood by considering a networked systems described by a global order parameter, e.g. its dynamics Γ or information capacity I, depends in a complex way on a network structure C, e.g. on the adjacency matrix $A=A_{\mathrm{ij}}$, or on some topological property T defined on such a matrix, and that lives in a space S, e.g. a manifold M with some metric g. Perhaps the most intuitive aspect one may investigate is the dynamic manifold's smoothness with respect to the connectivity matrix $\left(\partial M/\partial C\right).$ A further step may consist in finding how the manifold varies as some particular topological property changes $\left(\partial M/\partial T\right)$. When appraising resilience, though, what is usually addressed is how topology in the appropriate space itself varies with changes in connectivity $\left(\partial T/\partial C\right).$ The ultimate and more complex goal is understanding variations induced in the corresponding functional manifold ($\partial F\left(M\right)/\partial C$), and how these may occur as a result of changes in the system's structure.

Addressing resilience from a network perspective involves various important questions. Is there any structural characteristic that renders the system more vulnerable prior to or as a consequence of disease? How much perturbation can the system withstand to maintain structure? Can function be sustained without qualitatively changing structure? How can the system change in order to maintain function without changing structure? Can a system maintain function by changing structure? How much should a system change to maintain function? To what extent does the system's ability to revert to its prior configuration depend on the network structure?

### (1) Structural, dynamical and functional resilience

Resilience may in principle be referred to structural, dynamical, and functional aspects of a system (Lesne, 2008; Majhi *et al*., 2024). Brain structural robustness is defined as the degree to which network properties are resilient to sequences of such perturbations, typically of the anatomical network (Alstott *et al*., 2009; Warren *et al*., 2014; Aerts *et al*., 2016). Dynamical robustness is the ability of a system to conserve the same asymptotic dynamics (Lesne, 2008; Demongeot *et al*., 2010). In particular, it is important to understand dynamical robustness of networks of coupled oscillators (Daido & Nakanishi, 2004, 2007) in biological systems (Tanaka, Morino & Aihara, 2015). Insofar as dynamic oscillatory activity is crucial to healthy brain functioning (Başar, 1999; Fries, 2005) and compromised in various brain pathologies (Schnitzler & Gross, 2005), a meaningful way to define dynamical robustness is as the ability of a network to sustain collective macroscopic oscillations when some of its nodes fail to produce rhythmic dynamics as the system is perturbed locally (Tanaka, Morino & Aihara, 2012). Note that structural robustness may refer to both the anatomical and the dynamical structure and more generally to any aspect that can meaningfully be endowed with a network structure. Likewise, dynamical robustness could in principle refer to the anatomical structure at sufficiently long timescales. Given brain activity's complex phase space and the non‐trivial structure–dynamics–function map, structural and dynamical robustness may or may not imply functional robustness (Kitano, 2007; Schwarze *et al*., 2024). In particular, function may not be robust with respect to given perturbations that leave structure and/or dynamics unchanged.

### (2) Gauging the effects of disease: the solid material metaphor

Disease may lead to various degrees of impairment, ranging from decreased efficiency to complete breakdown of a given function. To understand disease and the associated damage in terms of some intrinsic properties of the affected system it is useful to think of the structure, whether defined in real or in phase space, as a solid material. In material science, resilience is related to the amount of energy a solid material can absorb and still recover its original state. This corresponds to its ability to remain in the elastic region of the stress–strain curve, obtained by gradually applying load and measuring the deformation on the tested material. This curve reveals various key properties of a material. Two important landmarks in the curve are represented by the yield point and the ultimate tensile strength. The former identifies the limit between elastic and plastic behaviour, below which the material deforms elastically returning to its original shape when the applied stress is removed, while it deforms irreversibly and plastically above it. The latter identifies the maximum stress that a material can withstand before breaking as it is being stretched or pulled, which lies close to the yield point for brittle materials and further away for ductile ones.

Altogether, the way the system withstands the impact of damage can be thought of in terms of toughness. Toughness quantifies the amount of energy that a material can absorb plastically before fracturing, and comprises both strength, i.e. the ability to withstand external forces without breaking or yielding and ductility, i.e. the ability to withstand plastic deformation before fracture.

Importantly, some properties (e.g. stiffness, a measure of a material's ability to return to its original form after being acted upon by an external force), depend on the system's shape and boundaries, while other properties (e.g. the elastic modulus, a measure of resistance to elastic deformation) are intrinsic (intensive) properties of a given material.

### (3) Resilience in the elastic regime: disease as brain perturbation

One general way to approach resilience and related concepts is to think of disease as a perturbation to the system and to evaluate how the system modulates the response to such a perturbation. Disease can then be thought of as a perturbation acting upon the network structure of a multi‐body dynamical system and pushing it towards dysfunctional regimes (Sanz Perl *et al*., 2023), and vulnerability and resilience are expressed in terms of action upon a network.

Key questions a perturbative model of resilience and pathology addresses are: (*i*) what aspect is showing robustness and against what kind of perturbation? (*ii*) What aspect of the network structure does disease act upon? (*iii*) Through what mechanism does disease act on the network? (*iv*) How does the network respond to perturbations? (*v*) How is damage evaluated?

#### (a) Perturbing brain network structure

Diseases are often represented as factors, e.g. errors or attacks, acting on network nodes or links, e.g. by deleting, weakening or perturbing them. These factors may come in two main modalities: random or targeted (Albert, Jeong & Barabási, 2000). In the simplest case, one considers node or link failure, corresponding to their respective deletion. While the effect of network structure can be separated from that of strength (Allesina & Tang, 2012), the impact of link failure on global network function is not related in a simple way to the latter. For instance, the breakdown of strong links may not be critical to network operation, whereas moderately weighted links may induce large‐scale performance impairment (Witthaut *et al*., 2016). Moreover, weak links may have a stabilising effect (Csermely, 2004). Overall, which links are critical is determined by the interplay between redundant capacity, a topological variable, and load distribution (Witthaut *et al*., 2016).

The consequences of structure perturbation depend not only on the perturbation but also on the structure itself. For instance, they show strong topology dependence. Topology‐dependent sensitivity to random and targeted attacks in isolated networks has long been highlighted (Albert *et al*., 2000). For instance, it has recently been shown that various dynamical processes unfolding on hierarchical directed networks can be disrupted by targeted attacks to a small fraction of links flowing from a higher to a lower hierarchical level (Rodgers, Tiño & Johnson, 2023). Moreover, different types of interdependent networks appear to be significantly more vulnerable than isolated networks, featuring first‐order (Buldyrev *et al*., 2010; Parshani, Buldryev & Havlin, 2010; Gao *et al*., 2011, 2012; Baxter *et al*., 2012; Radicchi & Arenas, 2013; Liu & Barabási, 2016; Gross, Bonamassa & Havlin, 2023a) or hybrid (Liu & Barabási, 2016) phase transitions at link lengths where the mutual giant component still emerges continuously (Gross, Bonamassa & Havlin, 2021) in contrast with the second‐order continuous phase transition found in isolated networks. Moreover, given the interconnected nature of brain anatomy and dynamics, these results would *prima facie* predict high brain instability. However, the stability of a system of networks results from the interplay between the internal network structure and its connectivity to other networks. In particular, the system of networks is stable and robust to failure if it is interconnected through network hubs, and the connections between networks are moderately assortative (Reis *et al*., 2014). Insofar as the effects of perturbations are network structure specific, robustness assessment requires a good characterisation of connectivity at the appropriate scale. In addition to topology at various scales, network geometry can also determine the rate function at which a network returns to its original state after a perturbation. In particular, the static anatomical network structure's Ollivier‐Ricci curvature can in principle be thought of as a proxy of structural network robustness, as a large curvature is associated with high relaxation rate to the equilibrium distribution (Sandhu *et al*., 2015).

The standard resilience framework is predicated upon the principle that disease acts through disconnection. Damage is thought of as network dismantling, i.e. how structure fragments into disconnected subsystems as a result of node or connection damage (Braunstein *et al*., 2016; Ren *et al*., 2019; Liu *et al*., 2022), and the functional effects of disease would be achieved by acting on some property of the network to achieve disconnection. For instance, in a directed graph, disease may remove the feedback vertex set, i.e. a subset of nodes that make the graph acyclic (Zhang, Garas & Scholtes, 2021). More generally, disease may act on the network's determining nodes, i.e. the subset of nodes whose dynamics is sufficient to determine the entire system's dynamics (Mochizuki *et al*., 2013).

Node or link loss is often modelled as a percolation process. For unweighted networks, such a process may involve units failing uniformly at random with some given probability. Alternative failure mechanisms, corresponding to different percolation processes may also exist. For instance, *k*‐core percolation involves the iterative removal of nodes with a degree smaller than *k* (Lahav *et al*., 2016; Xue *et al*., 2024). Node and link failure mechanisms have important dynamical consequences. For instance, in *k*‐core percolation on spatially embedded networks, link length can induce a metastable phase not present in standard percolation, wherein localised failure spontaneously propagates until the system collapses (Xue *et al*., 2024). Percolation has been used to probe neuronal resilience in the early stages of brain development. But, while percolation is an elegant way to investigate network properties can it also be understood as a disease's *modus operandi*?

An important issue concerns the deletion process and the extent to which connectedness (particularly anatomical) entails functionality. While intuitive in contexts such as transport or communication, this is not necessarily the case for all brain functions. Indeed, anatomical fragmentation is not a necessary condition for brain function impairment and functional breakdown may precede structural fragmentation. For instance, generalised or focal seizures may arise in the absence of localised brain damage (Terry *et al*., 2012). Moreover, removal of nodes or links central to network anatomical connectivity might have insignificant effects. More generally, pathology is indeed more generally defined by dysconnection rather than disconnection, as link strength and density may also be enhanced (Friston *et al*., 2016). Functional impairment may also result from failure to activate an otherwise intact anatomical structure. This may stem from noise on nodes but also from noise on links (Cavagna *et al*., 2024). Brain damage may also act by impairing cross‐scale activity flow, which can be studied by examining the response to damage of dynamical processes (Ghavasieh *et al*., 2023).

A further important issue is the representation of functional damage in terms of some aspect of network structure. The impact of a structural perturbation is typically quantified in terms of the largest connected component, i.e. the subnetwork that remains connected following the random removal of a fraction of its nodes, as a function of the pruning probability (Rapisardi *et al*., 2022). Network robustness may for instance be characterised in terms of the size the giant component or of the integrated size of the largest connected cluster during the entire attack process (Gao *et al*., 2015) or of the critical threshold value at which the giant component vanishes and the network becomes disconnected (Liu *et al*., 2022). Crucially, these criteria can be expressed in terms of structural properties of the network. For instance, for a random network, the critical threshold can be expressed as a function of the degree distribution (Cohen *et al*., 2000). The size of the largest connected component may undergo phase transitions at different thresholds or even of different types. But what physiological meaning should be ascribed to the largest connected component? It is reasonable to suppose that its meaning should be scale dependent (i.e. it should only be meaningful at some scales)? Moreover, connectedness provides no information on the resilience of network connectivity before or after attacks have taken place (Mohseni‐Kabir *et al*., 2021). Alternative criteria may be more sensible depending on the context. For instance, in transport, a more appropriate criterion is for a node to belong to the network's backbone, where information flow, or blood supply occurs. However, it is in general not straightforward to evaluate a given networked system's resilience in terms of the giant component *per se*, and other network properties, e.g. modularity, feedback loops (Csete & Doyle, 2002) or some non‐trivial network property possibly non‐local in the real space may be more appropriate indicators of functional resilience.

Alternative methods to evaluate stability are sometimes used. For instance, the relation between anatomical and dynamical network structure is often used to predict the amount of damage the brain can withstand due to lesions at a given location. While this may provide some indirect indication it is nonetheless not a clear indication of functional robustness. Moreover, structural perturbations can also be used to probe dynamical rather than structural robustness (Faci‐Lázaro *et al*., 2022).

Rather than in structural terms, resilience against structural perturbations should perhaps instead be evaluated in functional terms, e.g. in terms of information transport or information coding and capacity. For instance, error‐correcting codes, which encode information so as to guarantee its faithful transmission even in the presence of various sources of noise, and codes maximising the capacity of a network as a whole should differ in underlying network structure they induce, but also in terms of robustness, including the type of perturbation they are designed to cope with, and possibly in terms of the network adaptation following node or link failure that they require (Koetter & Médard, 2003; Koetter, Effros & Médard, 2011). Thus, it is expected that coding strategy and network structure interact to modify the functional consequences of perturbations. More generally, network structure may mediate trade‐offs between robustness resource demands and performance. For instance, redundancy enhances robustness against component failure, but for a constant resource level, it may degrade performance. It may also increase vulnerability against other types of perturbation (Kitano, 2007).

#### (b) Perturbing brain network dynamics

Instead of acting upon the system through node or link deletion, disease may challenge the system *via* a genuine matrix perturbation through infinitesimally small changes. While the former effectively changes the connectivity matrix's dimensionality and therefore constitutes a matrix change, the latter constitutes a genuine matrix perturbation.

Perhaps the simplest scenario is one in which oscillatory activity at node level gradually transitions into fixed points (Daido & Nakanishi, 2004). If enough nodes undergo such a transition, the system may transition towards a globally non‐oscillatory regime, a phenomenon termed ageing transition (Daido & Nakanishi, 2004). In a global network of diffusively coupled oscillators, ageing transition can be characterised by a universal scaling law of an order parameter controlled by the fraction of transitioning nodes and the coupling strength (Mahji *et al*., 2024). Ageing transition has been studied in various models with different coupling functions and network structures (Tanaka *et al*., 2014; Pazó & Montbrió, 2006; Thakur, Sharma & Sen, 2014). For instance, it has been shown that scale‐free networks are dynamically robust with respect to random inactivation but vulnerable to targeted inactivation of low‐degree oscillators. Remarkably, this stands in contrast to the role hubs play in the structural robustness of scale‐free networks. Furthermore, the presence of time delays in the coupling decreases dynamical robustness (Thakur *et al*., 2014), whereas dense network motifs enhance dynamical stability (Gross, Havlin & Barzel, 2023b). A further important question is what makes synchronous states more or less fragile against external perturbations (Tyloo, Coletta & Jacquod, 2018).

An important point is to do with the evaluation of resilience. Often, for instance, when intended as a measure of stability, resilience is evaluated *via* local linear measures and against infinitesimal perturbations. Brain dynamical resilience can for instance be defined as the minimum distance between all accessible dynamical regimes (Rings *et al*., 2019a). The larger this distance, the higher the brain's ability to withstand perturbation and reorganise, undergoing dynamical changes while retaining the same functionality. Dynamical resilience was found to increase in the hours preceding seizures (Rings *et al*., 2019a). However, to evaluate stability against generic perturbations, non‐local and non‐linear measures such as volume of basins of attraction, a measure of the likelihood of return to a given state after random not necessarily infinitesimal perturbation, may be preferable (Menck *et al*., 2013).

### (4) Network structure and resilience mechanisms in the elastic range

To understand the role played by brain network structure in disease prevention or persistence it is essential to characterise the mechanisms through which the system acts to enforce robustness. Robustness may for instance result from redundancy or degeneracy (Rule, O'Leary & Harvey, 2019; Jensen *et al*., 2022) and sloppiness, i.e. the dynamics may be sensitive to only a few combinations of parameters and insensitive to many other combinations (Daniels *et al*., 2008; Transtrum *et al*., 2015; Panas *et al*., 2015). Functional redundancy and degeneracy arise at many levels (Edelmann & Gally, 2001), although how they are organised and interact across scales is still poorly understood (Machta *et al*., 2013). Robustness has also been related to feedback and control mechanisms (Barkai & Leibler, 1997; Doyle & Csete, 2005). The main question here is what role network structure plays in the implementation of such mechanisms in the healthy brain and in disease: does structure ensure robustness of certain properties against a range of perturbations? Conversely, is network structure an emergent property of trade‐offs of systems favouring robustness?

Network structure may contribute to robustness in various function‐ and perturbation‐specific ways (Schwarze *et al*., 2024), in both real and phase space. For example, higher‐order interactions in real space may stabilise dynamics (Grilli *et al*., 2017). Network structure may also somehow constitute a sloppy parameter, although exactly how, at what scale and what properties should be determined. A further possibility is that network structure could provide topological protection (Murugan & Vaikuntanathan, 2017), characterised by insensitivity to fundamental properties of brain structure, e.g. modularity, dynamics or function, thus almost smoothing changes in parameters, with change then requiring a phase transition (Hasan & Kane, 2010), due to integer‐valued quantities called topological indices (Thouless *et al*., 1982; Wen, 1995). A classical example is the genus of a surface, which counts the number of holes in the surface (Ghrist, 2014). In phase space, the relational structure could constitute an obstruction to some functionally important property, e.g. convexity in neural codes (Ghrist, 2014; Giusti & Itskov, 2014; Curto *et al*., 2017). For instance, physiological properties of neuronal populations may be associated with code convexity, while codes with local obstructions are necessarily non‐convex (Giusti & Itskov, 2014; Curto *et al*., 2017; Lienkaemper, Shiu & Woodstock, 2017).

#### (a) Neutral structure

In many biological systems, e.g. in gene regulatory networks (Bergman & Siegal, 2003; Ciliberti, Martin & Wagner, 2007), resilience results from the presence of neutral networks (van Nimwegen, Crutchfield & Huynen, 1999; Schuster & Fontana, 1999; Wagner, 2008a) and the associated neutral space, i.e. the corresponding subspace in the phase space. In a genetic context, a neutral network is a set of equifunctional genes related by a point mutation, whose nodes represent gene sequences and whose links are mutations connecting them (van Nimwegen *et al*., 1999). Thus, the structure in the functional space is induced by the genotype‐to‐phenotype map and by the mechanisms controlling mutations. Similarly, in the context of RNA secondary structure, RNA sequences are mapped onto the genotype and the secondary structures onto which they fold play the role of phenotypes and what is considered is the phenotypic structure induced by the sequence‐to‐structure map (Schuster & Fontana, 1999; Wagner, 2008a).

In a neural context, a neutral network can be defined at various levels. For instance, it is natural to think of the physiological space as the genotype space, while the phenotype space may be identified with the space of network structures, e.g. of topologies, induced by the renormalisation of the underlying physiology. This space is both scale and renormalisation dependent. Finally, the functional space is identified as the functional or fitness space. Overall, one considers genotype‐to‐fitness maps (Stadler *et al*., 2001; Manrubia *et al*., 2021), which can be decomposed into a physiology‐to‐structure and structure‐to‐function map, highlighting the role played by the intermediate phenotype space. A neutral structure can in principle be defined at both genotype and phenotype levels. In the latter case, the neutral network is a structure whose nodes are network ensembles resulting in functionally equivalent phenotypes, and whose links are induced by the associated accessibility structure. The corresponding neutral space comprises regions in the space of network structures (or of parameters) that give rise to equivalent functional behaviour.

**Fig. 3:**
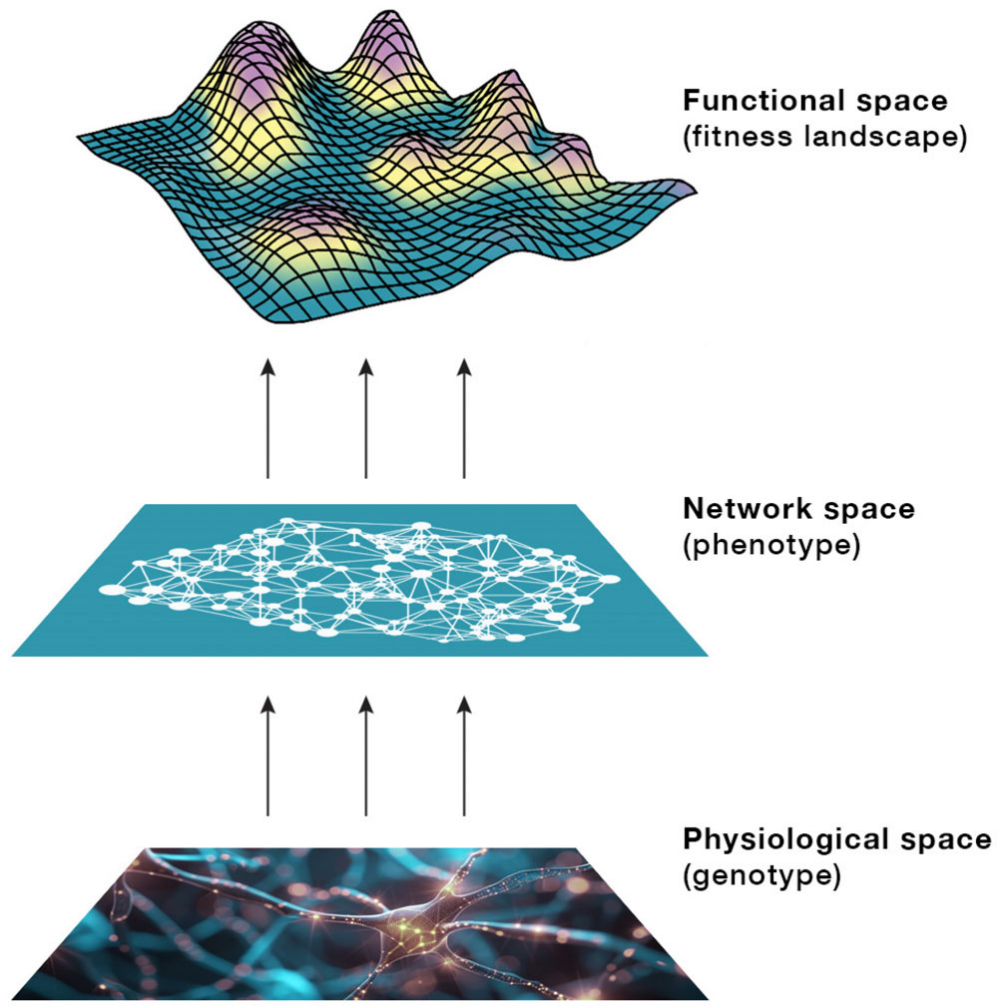
Functional brain activity can be thought of as the fitness landscape corresponding to a given network structure phenotype. In turn, the phenotype space of network structures emerges from the renormalisation of underlying space of physiological activity. Neutral structure can be defined at both genotype and phenotype level. The functional space's morphology depends on the properties of both the genotype‐to‐phenotype and phenotype‐to‐fitness maps.

The accessibility of phenotypes is determined by the interplay between the mechanisms generating variation and the structure of the genotype‐to‐phenotype map (Fig. 3). If functional phenotypes are organised according to accessibility of given network structure ensembles, the resulting functional space may have a non‐trivial structure. Note that, at both the genotype and the phenotype level, the neutral network is not defined in real space but rather in phase space, although each node may itself represent a topological pattern in real space renormalised up to a given scale, and that phenotypic neighbourhoods, which are induced by the genotype‐to‐phenotype map, may differ considerably from neighbourhoods in the real space. Robustness implies that the different genotypes of a given phenotype are close in the genotype space. Moreover, phenotypic transitions can be thought of as competitions between networks (Manrubia *et al*., 2021).

The presence of neutral networks may have important functions, e.g. allowing communication in the presence of noise as in error‐correcting codes (Hopfield, 1974; Sreenivasan & Fiete, 2011), and exploring the configuration space without dysfunctional phenotype changes.

#### (b) Extension of the elastic range: cognitive reserve and symptom onset

Often, a temporal gap separates disease onset from the appearance of observable symptoms, and a further time delay exists between symptom onset and complete functional breakdown. For instance, AD symptoms can be delayed in subjects with a high educational level in spite of neuropathological changes. It has been proposed that this phenomenon may be explained in terms of cognitive reserve (Mortimer, 1988; Le Carret *et al*., 2003; Buckner, 2004; Stern *et al*., 2019; Wilson *et al*., 2019). Following the material metaphor, how cognitive reserve shields the system with respect to symptomatic functional disruption could be understood in terms of extension of the elastic range width. One way to understand whether and how functional and behavioural changes are related to network structure may involve characterising the resilience of brain networks particularly of the hierarchical structure in ageing (Stanford, Mucha & Dayan, 2022), e.g. through *k*‐shell decomposition (Dorogovtsev, Goltsev & Mendes, 2006). [The *k‐core* of a graph G is the maximal subgraph of G having minimum degree at least *k*. The *k‐shell* of a graph G is the set of all nodes belonging to the *k*‐core of G but not to the (*k + 1*)‐core. The network is decomposed into node shells of increasing interconnectivity robustness, until a core set of nodes within a network is reached (Dorogovtsev *et al*., 2006). Attacks are performed on these networks, and the resilience of nodal shell and core are measured by extracting the *k*‐shell of the resulting network (Mohseni‐Kabir *et al*., 2021; Shang, 2019; Wang *et al*., 2022).] Core connectivity and resilience was found to decline with age (Stanford *et al*., 2022). Furthermore, differences in the organisation of functional networks among individuals with varying levels of cognitive reserve have been linked to the dynamical properties of specific brain regions. Notably, regions exhibiting higher connectivity strength tend to show increased entropy and reduced complexity, reflecting more stochastic and less organised neural dynamics within network hubs (Martínez *et al*., 2018b). On the other hand, cognitive reserve has been shown to be associated with increased redundancy in brain networks as quantified by the number of simple (non‐circular) paths between a pair of nodes up to a specified length (Stanford, Mucha & Dayan, 2024). Fundamental questions remain regarding the specificity but also irreducibility of such a result. For instance, what is attributed to *k*‐shell topology may in fact reduce to lower‐level properties such as degree distribution (Baxter *et al*., 2015). Finally, it is also unclear through what mechanism and against what class of perturbations can a particular network structure enforce the resilience of neural systems associated with cognitive reserve.

### (5) The role of network structure in brain reorganisation

As disease pushes the brain away from its neurophysiological structure, various dynamical scenarios are possible. First, the system may be able to reverse disease and self‐heal. This could drive the system to the original attractive state or to some equifunctional albeit different one from the situation prior to disease. Quasi‐neutral regimes may not be neighbours in the genotype and in the phenotype space, i.e. in the physiological space and in the way this renormalises into a particular network structure ensemble. They may for instance result in the recruitment of new degrees of freedom at the neurophysiological level, resulting in topologically different but equifunctional structure. On the opposite side of the spectrum, disease may be irreversible, although not lethal, and become chronic. This does not necessarily entail that it dwells in the dynamical regime directly induced by the disease. For instance, following adverse events such as strokes, the brain may head towards parts of the phase space which, although not equifunctional with respect to those corresponding to healthy functioning, are nonetheless functionally improved with respect to those of the acute phase. In doing so, the neuronal populations explore the neurophysiological configuration space. In such exploration the system can evolve and reorganise at scales often much larger than those of the perturbation, either slowly adapting through evolution or directly through phenotypic plasticity (Botero *et al*., 2015). In this regime, resilience may involve profound changes in some aspects of brain structure and dynamics. The exploration process can be characterised in various ways. For instance, once the brain has experienced the adverse functional effects of disease it can be visualised as undergoing learning to perform a new function in search of a fitness maximum (Stern, Liu & Balasubramanian, 2024). In a networked system, learning is typically associated with changes in the network structure (Stern & Murugan, 2023), and the corresponding neural activity is encoded in low‐dimensional manifolds (Gao & Ganguli, 2015).

Several important questions arise: what is the dynamics of such a wandering process? By what is it driven? Where does it lead to, i.e. what is the nature of the states or regimes it leads to? In all cases, what is the role of network structure? Is recovery associated with the attainment of a particular network structure? Conversely, is a particular network structure associated with the system's ability to evolve towards a state with improved function?

#### (a) The role of network structure in brain evolvability after injury

Insofar as disease can effectively be thought to exert effects similar to those of a mutation, more or less abruptly pushing the system towards non‐neutral (possibly non‐neighbouring) parts of the phase space (Kaneko, 2007; Ciliberti *et al*., 2007; Greenbury *et al*., 2016), one important question relates to the system's ability to evolve and improve functionality in particular through rewiring and changes in dynamics (Altenberg, 1995; Payne & Wagner, 2019). In this sense, the brain should be considered as an evolutionary capacitor buffering genotypic variations (Bergman & Siegal, 2003) and disease may be thought of as a loss not only of fitness and resilience, but also of evolvability. But what role does network structure play in brain evolvability? In particular, does disease affect evolvability by acting on network structure? Does disease reveal physiologically dormant phenotypes?

An essential ingredient for evolution is the heterogeneity in traits affecting function. In genetics, evolvability is quantified in terms of the variety of phenotypes lying within a given mutation distance of a genotype or phenotype (Wagner, 2008b). If disease drags the system to some suboptimal genotype, an important aspect is therefore phenotypic variability, i.e. the extent of phenotypic variation accessible to the genotype. A given genotype can either be robust, when surrounded by neighbours of the same phenotype, or evolvable, when its neighbourhood comprises a variety of other phenotypes. Phenotypic robustness may actually facilitate evolvability (Wagner, 2008b, 2011, 2012; Ahnert, 2017), due to the presence of neutral networks and genotype neighbourhoods, i.e. the set of genotypes accessible from a genotype in a given number of mutations, a measure of genotype's phenotypical variability (Wagner, 2008a). Genotypes within a neutral network can thereby explore different phenotypes. Translated in brain network terms, this means that neurophysiological activity should be able to renormalise into separable network ensembles.

A further fundamental question is whether there are network properties facilitating evolvability. In both real and phase space, an important property facilitating evolvability is represented by modular organisation (Simon, 1962). Interactions between different components provide a phenotypic space where mutations can produce variation without inducing adverse consequences. In phase space, phenotypic robustness and evolvability can be characterised in terms of network properties (Aguirre *et al*., 2018). For instance, the former can be defined through topological properties of the phenotype or of the functional space, the latter as the emergence of connected components of the neurophysiological space. In turn, this definition suggests that robustness of evolvability, may itself be a robust property, which can be quantified as the sensitivity to network perturbations of the topological measure quantifying evolvability (Ibañez‐Marcelo & Alarcon, 2014).

Finally, it is worth noting that if disease is a phenotype with its own structure, it should also be characterised by its own resilience and evolvability. For instance, cancer can be seen as a system robust with respect to its proliferative potential, which is robust against the action of the immune system and therapies (Kitano, 2004a,b), but also highly evolvable (Tian *et al*., 2011), so that effective control strategies must address both robustness and evolvability (Tian *et al*., 2011).

#### (b) Beyond the elastic range: learning, homeostasis, and adaptive resilience

Above, we have considered disease through an approach wherein a structure responds to some particular perturbation in an essentially passive way. In network terms, the proximal effects of such a perturbation are typically translated into local node or link damage, ranging from perturbation to breakdown, which may induce cascades of secondary effects. In this framework, disease onset and functional breakdown are respectively thought of as the system's yield point and optimal tensile strength. However, this approach does not take into account the fundamental tendency of living systems to maintain a given function rather than a given state or regime, e.g. a given network structure (Kitano, 2007). Moreover, we have considered resilience with respect to perturbations from elements acting on the system but which are not modified by it. The picture is bound to be different when brain and disease can effectively be thought of as interacting systems. In the same way that disease may directly affect the underlying network structure associated with healthy brain function and achieved during the course of development and learning prior to disease onset, the dynamics following disease may also affect (and be affected by) the network structure induced by the disease. This is particularly conspicuous in chronic disease.

The ability to absorb shocks and reorganise so as to retain the same function requires plastic mechanisms on a wide range of scales, from the rapid scales of sensory stimuli, to developmental and evolutionary ones, enabling the system to change beyond the elastic range. Brain function is subject to a trade‐off between stability and plasticity (James, 1890). On the one hand, it must produce stable representations to enable memory of previous knowledge. Specific network properties in the relevant space may characterise not only resting brain activity over timescales of minutes to hours, but also processes such as memory storage, where representation stability is required in the face of connectivity fluctuations (Susman *et al*., 2019; Rule *et al*., 2019). On the other hand, neural systems from cell to population levels must be able to match incoming stimuli, to integrate new knowledge and to learn from past experience in order to improve fitness but also to withstand phasic stress of various types (Mattson & Magnus, 2006; Rutkowski & Hegde, 2010; Roth & Balch, 2011) and to adapt to chronic stress (Saxena & Caroni, 2011). Correspondingly, network structure may play seemingly opposite roles under different circumstances.

Learning and adaptation are subserved by various classes of physiological mechanisms acting at various spatial and temporal scales. For instance, the structure can evolve as the result of mechanisms enabling learning of various forms (Clark & Abbott, 2024). However, an autocatalytic mechanism such as Hebbian learning would lead to runaway excitation and complete network synchronisation (Markram *et al*., 1997; Ocker, Litwi‐Kumar & Doiron, 2015; Zenke, Gerstner & Ganguli, 2017). A class of plasticity mechanisms capable of achieving dynamic stability in recurrent networks in the presence of Hebbian learning is represented by homeostatic plasticity, through which neurons control their own excitability, regulating spike rates or stabilising network dynamics at various timescales (Turrigiano *et al*., 1998; Turrigiano, 2011). Importantly, in the neocortex, correlation structure of local networks (Wu *et al*., 2020) and network criticality (Shew *et al*., 2015; Ma *et al*., 2019) are under homeostatic regulation (Wen & Turrigiano, 2024; Zeraati, Priesemann & Levina, 2020; Landmann, Baumgarten & Bornholdt, 2021; Menesse, Marin & Kinouchi, 2022). Plasticity may dynamically target criticality by adjusting excitatory–inhibitory connectivity to address excitation/inhibition imbalances in neuronal populations (Sukenik *et al*., 2021).

Homeostatic plasticity mechanisms are slow negative feedback loops (Ma *et al*., 2009; Zierenberg, Wilting & Priesemann, 2018; Golubitsky & Wang, 2020) implemented by several neurophysiological mechanisms. For instance, synaptic scaling (Markram & Tsodyks, 1996; Turrigiano *et al*., 1998; Fong *et al*., 2015; De Vivo *et al*., 2017) allows conserving the overall neural activity in the presence of exogenous perturbations or damage, by adjusting synaptic strength (Murphy & Corbett, 2009), membrane excitability (Davis, 2006; Pozo & Goda, 2010), or neuron–glial interactions (de Pittà, Brunel & Volterra, 2016).

Through appropriate combinations of plasticity mechanisms, the brain can optimise wiring by creating or pruning connections as well as weight distribution (Estévez‐Priego *et al*., 2020; Teller *et al*., 2020; Schwarze *et al*., 2024), reshaping the network structure at all scales and generating complex dynamical regimes (de Arcangelis, Perrone‐Capano & Herrmann, 2006; Levina, Hermann & Geisel, 2007, 2009; Meisel & Gross, 2009; Rubinov *et al*., 2011). For instance, interactions between Hebbian learning and single‐cell homeostasis allow neurons to exploit redundancy in order reliably to read an evolving population code in the presence of gradual tuning drift (Rule & O'Leary, 2022). Notably, at least in principle, different plasticity rules can give rise to indistinguishable network dynamics (Ramesh *et al*., 2023).

Plasticity‐induced rewiring can conceivably achieve both improved functional states and higher resilience through various mechanisms. For instance, it can effectively enforce allostery, a mechanism whereby an event at one site affects the activity at a distinct site, enabling the regulation of the corresponding function. Furthermore, self‐regulatory mechanisms such as neural scaling triggered by damage could adjust link strength through filtration processes, whereby all links with a weight strictly below a given threshold are removed (Rapisardi *et al*., 2022), mitigating through an active process the damage associated with link removal. Notably, these processes may involve parts of the system not confined to the one directly undergoing stress, and cells may proactively summon stress pathways to cope with higher demands. For instance, when cells initiate processes increasing pressure on homeostatic systems they also activate stress pathways to meet the associated physiological needs (Rutkowski & Hegde, 2010).

#### (c) Significance of network structure in functional reorganisation after brain injury

Plasticity mechanisms can change real space network structure on a wide range of scales, but several fundamental issues related to plasticity following disease are still poorly understood. First, while there is no consensus on the potential and limits of functional reorganisation after injury (Makin & Krakauer, 2023), it is unclear whether the potential for recovery is associated with some specific set of network properties, particularly in real space. Likewise, no consensus exists as to whether functional reorganisation is a maladaptive response to injury (Penner & Aktas, 2017; Rocca & Filippi, 2017). Second, damage is in general thought to exert a disruptive effect on structure, but it could also in principle directly or indirectly promote functionally relevant non‐trivial surrogate structure. For instance, in optimised transport networks damage can induce the formation of loops (Katifori, Szöllösi & Magnasco, 2010; Kaiser, Ronellenfitsch & Witthaut, 2020). On the other hand, the structure associated with functional reorganisation need not be functional, particularly past tipping points. But can disease‐induced functional and dysfunctional structure be discriminated in terms of network structure?

#### (d) Structural consequences of aberrant plasticity

Disease can act in two ways: either by overcoming the system's ability to adapt plastically or by interfering with plastic mechanisms themselves. Plasticity is a necessary condition for brain function and both excessive plasticity or dysfunctional homeostasis render the brain unable to adjust to changing demands and excessively vulnerable to environmental perturbations (Pascual‐Leone *et al*., 2011). Various pathologies, including developmental disorders or neurodegenerative diseases may be related to altered plasticity (Hallett, 2005; Oberman *et al*., 2014). The disorder phenotype is specified by the particular combination of environmental factors, genetic abnormalities and their expression timing, the affected cortical network and synapse type and the direction in which it is affected (Oberman & Pascual‐Leone, 2013). For instance, idiopathic ASD can be thought of as a plasticity disorder characterised by altered local cortical excitation/inhibition balance and brain network functional connectivity, resulting from genetic and environmental factors leading to aberrant excitatory plasticity (Oberman *et al*., 2014). At the cellular level, ASD may stem from increased axonal density and complexity and a reduction in synapse pruning and elimination resulting from gamma‐aminobutyric acid (GABA) synthesis blockade (Wu *et al*., 2012). On the other hand, neurodegenerative diseases may be initiated by chronic, possibly interacting, perturbations acting upon homeostatic mechanisms at various scales. For instance, the early stage of AD has been suggested to arise from an imbalance between firing homeostasis and synaptic plasticity (Styr & Slutsky, 2018). Dysfunction could become symptomatic when disease‐related stress exceeds the system's ability to withstand it. Disease may be aggravated by systemic feedback, e.g. through inflammation and immune responses, which can ultimately lead to neuronal degeneration and death. Disease may also affect the hierarchical unfolding of plasticity‐related neurobiological events during neural development. For instance, various developmental psychopathologies are characterised by protracted plasticity in association cortices (Sydnor *et al*., 2021).

But what is the role of network structure in aberrant plasticity? The main question is whether functional impairment in hyperplastic pathologies can be traced back to changes in the associated network structure. For instance, plasticity and adaptiveness may underlie increased pre‐seizure dynamical resistance and reduced medication effectiveness (Rings *et al*., 2019a; Zaveri *et al*., 2020; Lehnertz *et al*., 2023).

#### (e) Beyond the plastic range: disease as structure failure and its structural harbingers

Brain disease often leads to complete breakdown of function. In terms of the material metaphor, this corresponds to the ultimate tensile stress. An important question is whether this point can be predicted and, if so, whether it is accompanied by specific network properties in real and in phase space.

In strongly disordered non‐hierarchical networks, micro‐crack accumulation preceding failure is characterised by avalanches with self‐affine scaling behaviour. Failure can then be thought of as a critical phenomenon and avalanche size as an order parameter, whose change constitutes a failure precursor. From a dynamical viewpoint, failure is correspondingly described in terms of either damage percolation or crack nucleation‐and‐growth. By contrast, in hierarchical materials, fracture microstructure is generically scale‐invariant with self‐similar patterns at different scales even very far from the critical load (Moretti & Zeiser, 2019; Moretti *et al*., 2018, 2019). Failure is announced by two different properties, i.e. increased eigenvector localisation and decreased topological dimension (Moretti *et al*., 2018). Importantly, such a structure is associated with diminished crack propagation, containing damage spreading, and with changes in intrinsic material properties such as increased toughness (Moretti *et al*., 2018).

The material metaphor could conceivably apply to anatomical networks, but can it also be used to make sense of dynamic network resilience? In particular, are brain transition points associated with network biomarkers in brain dynamics? At long timescales, an important problem is whether the transition to fully fledged neurodegenerative conditions such as AD from its prodromes are associated with and predictable based upon corresponding network features.

At shorter timescales, network‐based early‐warning signs of imminent bifurcations or sudden deterioration have been reported (Chen *et al*., 2012; Liu *et al*., 2015). For instance, although transitions may not be characterised by classical features of criticality (Wilkat *et al*., 2019), seizures have been shown to be preceded by a decrease of neuronal network resilience which determines the ictogenic potential of interictal synaptic perturbations (Chang *et al*., 2018). On the other hand, while temporal changes of single nodes and links properties reported were found to be predictive of seizures (Andrzejak *et al*., 2003), the global properties of dynamical networks have been found to hold only limited predictive power (Kuhnert, Elger & Lehnertz, 2010; Geier *et al*., 2015). At macroscopic scales, seizure precursor generation may result from a rearrangement of the epileptic network's path structure, eventually resulting in the formation of bottlenecks altering activity spreading (Rings *et al*., 2019b). The earliest indications for such changes may occur hours prior to seizure onset and stem from connectivity changes within and between brain regions distant from the seizure onset zone corresponding to changes in the time‐varying centrality of the associated nodes. These regions, which are part of the large‐scale epileptic network, generate and sustain physiological brain dynamics during the inter‐ictal state, control the dynamics within the seizure onset zone (Lehnertz & Dickten, 2015; Johnson *et al*., 2023), and may modulate seizure dynamics (Geier *et al*., 2015). On the other hand, indicators found minutes before a seizure in the spatial proximity to the seizure onset zone appear to be epiphenomena of earlier harbingers of ictogenesis (Rings *et al*., 2019b).

### (6) Preventing and curing disease through network‐based intervention

From a clinical viewpoint, it is important not just to understand how the brain copes with pathological perturbations but also to prevent or reverse disease, through therapeutical interventions including pharmacological medication, surgical procedures, brain stimulation, neurofeedback or behavioural therapy (Flanagan *et al*., 2019; Papo, 2019b). But is it possible to prevent a system from approaching potentially pathological regimes or to push it away from such a regime once already in that state by acting upon brain network structure? Can resilience be increased (or disease resilience decreased) by acting on network structure?

Available strategies are essentially of two types: lesioning or restorative. The former involve surgery designed to shift the system to a functionally equivalent structure, effectively constituting an antifragile strategy, i.e. damaging a targeted structure to improve the performance of a global function (Taleb, 2012). The latter comprise stimulation strategies from chronic deep brain stimulation to phasic stimulation and neurofeedback.

A paradigmatic example of a network‐based intervention strategy is represented by seizure control procedures (Jirsa *et al*., 2017; Olmi *et al*., 2019). At the most basic level, seizure relief can sometimes be achieved by limiting seizure propagation, rather than addressing the seizure itself, the underlying principle being that cutting a dysfunctional network into pieces may restore its functionality (Ren *et al*., 2019). This can be achieved *via* resective and non‐resective surgery, ablation or stimulation strategies (Morrell, Whisler & Bleck, 1989; Papo *et al*., 1990; Dogali *et al*., 1993). On the other hand, while multiple focal ablations within the epileptogenic zone, i.e. the portion of cortical tissue necessary and sufficient for seizure initiation (Luders, Engel & Munari, 1993), may completely abolish epileptiform activity, such a strategy may also rebalance the network and hinder seizure propagation when targeting outside the epileptogenic zone (Olmi *et al*., 2019).

A proper intervention strategy involves defining, in addition to a general goal, a neural target as a feature, including *what* aspect of brain activity to act upon and *where* in the brain, and an appropriate stimulation schedule (Papo, 2019b). What makes network‐based interventions more arduous than standard local interventions? At the most basic level, a purely network‐based approach may involve intervention at multiple sites. More substantially, such an approach adds a complex factor to the control of dynamics, implying the identification and specification of a structure, in real or in phase space, as well as an understanding of the dynamical and ultimately the functional role of such a structure in the system's behaviour. Such understanding may exist, at macroscopic scale precisely when the underlying neural system works in a relatively simple way, e.g. as a feedforward loop such as in basal ganglia loops. The extent to which the relevant network structure must be known for the intervention to be effective, e.g. in seizure control, is a matter of current debate (Zaveri *et al*., 2020). Network theory suggests various methods to protect a network‐of‐networks from cascading failure, e.g. protecting high‐degree nodes against failures and attacks (Schneider *et al*., 2013), forcing dependency relations between nodes of similar degree (Parshani *et al*., 2011) or ensuring interconnections of independent parts through nodes with particular degree (Aguirre *et al*., 2013; Reis *et al*., 2014). However, at a practical level, the main issue is finding a way to act on the network in accordance. For instance, it was proposed that network connectivity in AD could be preserved by altering neuronal excitability (de Haan *et al*., 2017). Moreover, intrinsic properties of neuronal circuits such as plasticity or degeneracy can sometimes complicate therapeutic intervention. For example, changes in several ion channels are sufficient although not necessary conditions for hyperexcitability in primary somatosensory neurons induced by nerve damage in neuropathic pain, so that addressing degeneracy requires an integrative approach to drug discovery (Ratté & Prescott, 2016).

#### (a) Control and targeting of brain networks in disease

It is straightforward to think of medical intervention as a control problem, wherein brain dynamics, pushed away from the neurophysiological regime by disease, must be nudged (in finite time) towards the basin of attraction of a functionally more desirable dynamical regime, to which the system would not (or would too slowly) spontaneously evolve. This can be implemented by finding an appropriate control parameter for the dynamics and a feasible path from the initial condition to the target dynamical regime and altering either the system's dynamical equations (Ott, Grebogi & Yorke, 1990; Boccaletti *et al*., 2000; Lai, 2014) or its initial condition (Cornelius, Kath & Motter, 2013), either stabilising a specific trajectory through the application of small time‐dependent perturbations, or steering the dynamics towards a trajectory compatible with the system's natural dynamics but originating from a different initial condition, a procedure called targeting (Shinbrot *et al*., 1990). Moving a spatially extended networked system into any desired state within a given continuous phase space volume adds further complexity to the control problem associated with the spatial and relational nature of the controlled system (Liu & Barabási, 2016). Both control and targeting are in general conceptually approached through local perturbations of nodes or links.

Control methods could in principle be used for network reprogramming, for example, to shove brain dynamics away from epileptic or epileptogenic regimes (Cornelius *et al*., 2013), or to promote a particular behaviour of the system (Gutiérrez *et al*., 2012). More fundamentally, network control theory could in principle be used to probe cognitive resilience (Medaglia *et al*., 2017; Khona, Chandra & Fiete, 2023). Within this context, it is straightforward to address the following questions: what dynamical states are accessible in a stable way, starting from a given initial condition? What is the minimal number of nodes or links that need to be perturbed to reach a given desired dynamic regime? How costly is achieving states with given performance levels?

Recently various studies have tried assessing brain controllability [see Bassett, Khambhati & Grafton (2017) for a review]. Often, however, these studies are predicated upon rather unrealistic hypotheses on brain activity: brain resting dynamics is described in terms of a set of differential equations linearised around a dominant fixed point and connectivity dynamics is assumed to be linear and time‐invariant (Kim *et al*., 2018). Control of networked non‐linear dynamical systems faces a number of other challenges (Sun and Motter, 2013; Liu & Barabási, 2016; Zañudo, Yang & Albert, 2017; Tu *et al*., 2018). For instance, control trajectories are in general non‐local in the phase space, i.e. moving the system from the initial to the target state may require a very long path, irrespective of the distance between these two points in phase space; there is also a non‐locality trade‐off whereby either the control trajectory is non‐local in the phase space or the control inputs are non‐local in the network (Sun & Motter, 2013). More generally, ascertaining whether the system is controllable, that is, whether it can be driven from any initial condition to any desired state in finite time (Kálmán, 1963) typically requires extensive knowledge of the system's state space, and sometimes of its dynamics (Cornelius *et al*., 2013), information usually unavailable for system‐level brain activity. Furthermore, given the heterogeneity and multiscale character of brain dynamics, it is highly non‐trivial to determine both the overall effects of anatomically local stimulation and the level of coarse‐graining which would guarantee appropriate control targets. An additional issue is given by control costs. In the case of brain‐stimulation techniques, how much power is needed to achieve control and whether this is compatible with safety are fundamental issues. While network control methods aim at controlling dynamics through a minimal number of nodes or links, too exiguous a number may exact an excessive energetic cost (Yan *et al*., 2015). Contrary to reports suggesting resting brain activity controllability through a single node representing a given brain region (Gu *et al*., 2015), a recent study (Tu *et al*., 2018) showed that even though brain networks might be structurally controllable, the energy required to control it may be disproportionately high.

#### (b) Recovering from disease through microscopic interventions

Often, treatment strategies, particularly those using brain‐stimulation techniques, are predicated upon the idea that damage can be reversed by reconstructing the healthy regime's structure, e.g. by retrieving damaged nodes or links or, when this is not possible, by inducing a topologically and functionally equivalent one. However, due to the presence of path‐dependence, restoring topology, although possibly necessary, is in general not sufficient to return to the functional state. To address this issue, one possible strategy may consist in identifying states in which the system can be acted upon through microscopic interventions, for instance, by merely controlling a few nodes (Sanhedrai *et al*., 2022; Sanhedrai & Havlin, 2023). This strategy eliminates potential undesired inactive states, keeping the system in its healthy regime, without moving it as in control strategies. Moreover, while control strategies typically act locally, this strategy can act globally in the system's phase space (Sanhedrai *et al*., 2022).

## VII. CONCLUSIONS

(1) Over the past few years, it has become standard to think of brain anatomy and dynamics as systems whose non‐trivial network structure plays an essential role in healthy brain function. This assumption, predicated upon theoretical studies and experimental results from systems often rather different from neural systems, allows various angles and insights on disease, its consequences and the possible ways it can evolve and be acted upon. Overall, the intrinsic relational structure of brain anatomy and dynamics can be thought to mediate fundamental functional trade‐offs between efficiency, functional reliability, and evolvability, which ultimately determine both vulnerability to and the resilience of disease (Csete & Doyle, 2002, 2004; Kitano, 2007; Doyle & Csete 2011).
(2) If the brain indeed constitutes a genuine networked system, i.e. non‐trivial network structure plays an essential role in healthy brain function, then this structure could fundamentally affect or be affected by disease. Reciprocally, if network structure is essential to disease phenomenology, its characterisation could be an important step in the understanding of the role of network structure in function. Moreover, if network structure is essential to both function and dysfunction, it could in principle be used for clinical purposes (Castellanos *et al*., 2013). For instance, it could define patient theratypes separating therapy responders from non‐responders by encoding the structural mechanisms underlying disease expression and response to therapy (Agache & Akdis, 2019; Mobasheri & Loeser, 2024). Finally, if brain function and dysfunction become observable through network structure they could also be controllable through it. Thus, various fundamental questions concerning the role of complex network structure in disease, including whether there are genuine brain network diseases or whether network neuroscience can be used for disease classification (Douw *et al*., 2019), and more generally, for clinical purposes, hinge on the role of such a structure in brain function in general.
(3) The exact role of higher order network properties in disease aetiology and phenomenology is in general insufficiently understood. This includes the appropriate space and scale in which a network structure may be meaningfully defined, the particular type of network property, as well as the way these may exert a role in brain function and dysfunction.
(4) At a theoretical level, it is still unclear whether there exist genuine network‐related pathologies. Insofar as the role of complex network structure is disease dependent, it may in general be more useful to think of networkness not as an all‐or‐nothing phenomenon, together with a set of necessary and sufficient conditions for its occurrence, but in terms of role of network structure in disease whose significance can only be determined by considering the brain function it supports and the way it does it.
(5) At a practical level, the sensitivity and specificity of current network theory is not (yet) sufficient to make it clinically relevant.
(6) The role of network geometry, topology and combinatorics in brain function and dysfunction is still insufficiently understood, and qualitative improvements require addressing the conceptual and methodological challenges represented by the translation of properties such as efficiency, homeostasis robustness, and evolvability in network terms that are also functionally meaningful when describing the brain (Levit‐Binnun & Golland, 2012).

## References

[brv70086-bib-0001] Abdelnour, F. , Mueller, S. & Raj, A. (2015). Relating cortical atrophy in temporal lobe epilepsy with graph diffusion‐based network models. PLoS Computational Biology 11, e1004564. 26513579 10.1371/journal.pcbi.1004564 PMC4626097

[brv70086-bib-0002] Abdelnour, F. , Voss, H. U. & Raj, A. (2014). Network diffusion accurately models the relationship between structural and functional brain connectivity networks. NeuroImage 90, 335–347. 24384152 10.1016/j.neuroimage.2013.12.039 PMC3951650

[brv70086-bib-0003] Acero‐Pousa, I. , Escrichs, A. , Dagnino, P. C. C. , Sanz Perl, Y. , Kringelbach, M. L. , Uhlhaas, P. J. & Deco, G. (2024). Reconfiguration of functional brain hierarchy in schizophrenia. bioRxiv 2024‐08. 10.1101/2024.08.27.608945. PMC12501247 41053029

[brv70086-bib-0004] Adams, J. H. , Doyle, D. , Ford, I. , Gennarelli, T. A. , Graham, D. I. & McLellan, D. R. (1989). Diffuse axonal injury in head injury: definition, diagnosis, and grading. Histopathology 15, 49–59. 2767623 10.1111/j.1365-2559.1989.tb03040.x

[brv70086-bib-0005] Adams, J. H. , Graham, D. I. , Scott, G. , Parker, L. S. & Doyle, D. (1980). Brain damage in fatal non‐missile head injury. Journal of Clinical Pathology 33, 1132. 7451661 10.1136/jcp.33.12.1132 PMC1146364

[brv70086-bib-0006] Aerts, H. , Fias, W. , Caeyenberghs, K. & Marinazzo, D. (2016). Brain networks under attack: robustness properties and the impact of lesions. Brain 139, 3063–3083. 27497487 10.1093/brain/aww194

[brv70086-bib-0007] Agache, I. & Akdis, C. A. (2019). Precision medicine and phenotypes, endotypes, genotypes, regiotypes, and theratypes of allergic diseases. Journal of Clinical Investigation 129, 1493–1503. 30855278 10.1172/JCI124611 PMC6436902

[brv70086-bib-0008] Aguirre, J. , Catalán, P. , Cuesta, J. A. & Manrubia, S. (2018). On the networked architecture of genotype spaces and its critical effects on molecular evolution. Open Biology 8, 180069. 29973397 10.1098/rsob.180069 PMC6070719

[brv70086-bib-0009] Aguirre, J. , Papo, D. & Buldú, J. M. (2013). Successful strategies for competing networks. Nature Physics 9, 230–234.

[brv70086-bib-0010] Aguirre, J. , Sevilla‐Escoboza, R. , Gutiérrez, R. , Papo, D. & Buldú, J. M. (2014). Synchronization of interconnected networks: the role of connector nodes. Physical Review Letters 112, 248701. 24996113 10.1103/PhysRevLett.112.248701

[brv70086-bib-0011] Ahnert, S. E. (2017). Structural properties of genotype–phenotype maps. Journal of the Royal Society Interface 14, 20170275. 28679667 10.1098/rsif.2017.0275 PMC5550974

[brv70086-bib-0012] Albert, R. & Barabási, A. L. (2002). Statistical mechanics of complex networks. Reviews of Modern Physics 74, 47.

[brv70086-bib-0013] Albert, R. , Jeong, H. & Barabási, A. L. (2000). Error and attack tolerance of complex networks. Nature 406, 378–382. 10935628 10.1038/35019019

[brv70086-bib-0014] Alexander‐Bloch, A. F. , Gogtay, N. , Meunier, D. , Birn, R. , Clasen, L. , Lalonde, F. , Lenroot, R. , Giedd, J. & Bullmore, E. T. (2010). Disrupted modularity and local connectivity of brain functional networks in childhood‐onset schizophrenia. Frontiers in Systems Neuroscience 4, 147. 21031030 10.3389/fnsys.2010.00147 PMC2965020

[brv70086-bib-0015] Alivisatos, A. P. , Chun, M. , Church, G. M. , Deisseroth, K. , Donoghue, J. P. , Greenspan, R. J. , McEuen, P. L. , Roukes, M. L. , Sejnowski, T. J. , Weiss, P. S. & Yuste, R. (2013). The brain activity map. Science 339, 1284–1285. 23470729 10.1126/science.1236939 PMC3722427

[brv70086-bib-0016] Alivisatos, A. P. , Chun, M. , Church, G. M. , Greenspan, R. J. , Roukes, M. L. & Yuste, R. (2012). The brain activity map project and the challenge of functional connectomics. Neuron 74, 970–974. 22726828 10.1016/j.neuron.2012.06.006 PMC3597383

[brv70086-bib-0017] Allen, N. E. , Sudlow, C. , Peakman, T. , Collins, R. & UK Biobank (2014). UK biobank data: come and get it. Science Translational Medicine 6, 224ed4. 10.1126/scitranslmed.3008601 24553384

[brv70086-bib-0018] Allesina, S. & Tang, S. (2012). Stability criteria for complex ecosystems. Nature 483, 205–208. 22343894 10.1038/nature10832

[brv70086-bib-0019] Alstott, J. , Breakspear, M. , Hagmann, P. , Cammoun, L. & Sporns, O. (2009). Modeling the impact of lesions in the human brain. PLoS Computational Biology 5, e1000408. 19521503 10.1371/journal.pcbi.1000408 PMC2688028

[brv70086-bib-0020] Altenberg, L. (1995). The schema theorem and Price's theorem. In Foundations of Genetic Algorithms (Volume 3), pp. 23–49. Morgan Kaufmann, Saint Louis, MO.

[brv70086-bib-0021] Amari, S. I. & Nagaoka, H. (2007). Methods of Information Geometry, Edition (Volume 191). American Mathematical Soc, Providence, RI.

[brv70086-bib-0022] Amunts, K. , Ebell, C. , Muller, J. , Telefont, M. , Knoll, A. & Lippert, T. (2016). The human brain project: creating a European research infrastructure to decode the human brain. Neuron 92, 574–581. 27809997 10.1016/j.neuron.2016.10.046

[brv70086-bib-0023] Andrzejak, R. G. , Mormann, F. , Kreuz, T. , Rieke, C. , Kraskov, A. , Elger, C. E. & Lehnertz, K. (2003). Testing the null hypothesis of the nonexistence of a preseizure state. Physical Review E 67, 010901. 10.1103/PhysRevE.67.010901 12636484

[brv70086-bib-0024] Arenas, A. , Díaz‐Guilera, A. , Kurths, J. , Moreno, Y. & Zhou, C. (2008). Synchronization in complex networks. Physics Reports 469, 93–153.

[brv70086-bib-0025] Ashwin, P. , Fadera, M. & Postlethwaite, C. (2024). Network attractors and nonlinear dynamics of neural computation. Current Opinion in Neurobiology 84, 102818. 38070404 10.1016/j.conb.2023.102818

[brv70086-bib-0026] Avena‐Koenigsberger, A. , Goñi, J. , Solé, R. & Sporns, O. (2015). Network morphospace. Journal of the Royal Society Interface 12, 20140881. 25540237 10.1098/rsif.2014.0881 PMC4305402

[brv70086-bib-0027] Avena‐Koenigsberger, A. , Misic, B. & Sporns, O. (2018). Communication dynamics in complex brain networks. Nature Reviews Neuroscience 19, 17–33. 10.1038/nrn.2017.149 29238085

[brv70086-bib-0028] Babson, E. , Barcelo, H. , de Longueville, M. & Laubenbacher, R. (2006). Homotopy theory of graphs. Journal of Algebraic Combinatorics 24(2006), 31–44.

[brv70086-bib-0029] Bao, X. , Hu, Q. , Ji, P. , Lin, W. , Kurths, J. & Nagler, J. (2022). Impact of basic network motifs on the collective response to perturbations. Nature Communications 13, 5301. 10.1038/s41467-022-32913-w PMC9458749 36075905

[brv70086-bib-0030] Barkai, N. & Leibler, S. (1997). Robustness in simple biochemical networks. Nature 387, 913–917. 9202124 10.1038/43199

[brv70086-bib-0031] Barulli, D. & Stern, Y. (2013). Efficiency, capacity, compensation, maintenance, plasticity: emerging concepts in cognitive reserve. Trends in Cognitive Sciences 17, 502–509. 24018144 10.1016/j.tics.2013.08.012 PMC3840716

[brv70086-bib-0032] Barzel, B. & Barabási, A. L. (2013). Universality in network dynamics. Nature Physics 9, 673–681. 24319492 10.1038/nphys2741 PMC3852675

[brv70086-bib-0033] Barzel, B. , Liu, Y. Y. & Barabási, A. L. (2015). Constructing minimal models for complex system dynamics. Nature Communications 6, 7186. 10.1038/ncomms8186 25990707

[brv70086-bib-0034] Barzon, G. , Artime, O. , Suweis, S. & De Domenico, M. (2024). Unraveling the mesoscale organization induced by network‐driven processes. Proceedings of the National Academy of Sciences of the United States of America 121, e2317608121. 38968099 10.1073/pnas.2317608121 PMC11252804

[brv70086-bib-0035] Başar, E. (1999). Brain Function and Oscillations [v. II]: Integrative Brain Function, Neurophysiology and Cognitive Processes. Springer, Berlin Heidelberg.

[brv70086-bib-0036] Bashan, A. , Bartsch, R. P. , Kantelhardt, J. W. , Havlin, S. & Ivanov, P. C. (2012). Network physiology reveals relations between network topology and physiological function. Nature Communications 3, 702. 10.1038/ncomms1705 PMC3518900 22426223

[brv70086-bib-0037] Bassett, D. S. , Khambhati, A. N. & Grafton, S. T. (2017). Emerging frontiers of neuroengineering: a network science of brain connectivity. Annual Review of Biomedical Engineering 19, 327–352. 10.1146/annurev-bioeng-071516-044511 PMC6005206 28375650

[brv70086-bib-0038] Baxter, G. J. , Dorogovtsev, S. N. , Goltsev, A. V. & Mendes, J. F. F. (2012). Avalanche collapse of interdependent networks. Physical Review Letters 109, 248701. 23368399 10.1103/PhysRevLett.109.248701

[brv70086-bib-0039] Baxter, G. J. , Dorogovtsev, S. N. , Lee, K. E. , Mendes, J. F. F. & Goltsev, A. V. (2015). Critical dynamics of the k‐core pruning process. Physical Review X 5, 031017.

[brv70086-bib-0040] Bergman, A. & Siegal, M. L. (2003). Evolutionary capacitance as a general feature of complex gene networks. Nature 424, 549–552. 12891357 10.1038/nature01765

[brv70086-bib-0041] Bernal, J. D. & Mason, J. (1960). Packing of spheres: co‐ordination of randomly packed spheres. Nature 188, 910–911.

[brv70086-bib-0042] Berner, R. , Polanska, A. , Schöll, E. & Yanchuk, S. (2020). Solitary states in adaptive nonlocal oscillator networks. The European Physical Journal Special Topics 229, 2183–2203.

[brv70086-bib-0043] Bernhardt, B. C. , Bonilha, L. & Gross, D. W. (2015). Network analysis for a network disorder: the emerging role of graph theory in the study of epilepsy. Epilepsy & Behavior 50, 162–170. 26159729 10.1016/j.yebeh.2015.06.005

[brv70086-bib-0044] Bernhardt, B. C. , Chen, Z. , He, Y. , Evans, A. C. & Bernasconi, N. (2011). Graph‐theoretical analysis reveals disrupted small‐world organization of cortical thickness correlation networks in temporal lobe epilepsy. Cerebral Cortex 21, 2147–2157. 21330467 10.1093/cercor/bhq291

[brv70086-bib-0045] Bernhardt, B. C. , Worsley, K. J. , Kim, H. , Evans, A. C. , Bernasconi, A. & Bernasconi, N. (2009). Longitudinal and cross‐sectional analysis of atrophy in pharmacoresistant temporal lobe epilepsy. Neurology 72, 1747–1754. 19246420 10.1212/01.wnl.0000345969.57574.f5 PMC2827310

[brv70086-bib-0046] Bialonski, S. & Lehnertz, K. (2013). Assortative mixing in functional brain networks during epileptic seizures. Chaos 23, 33139. 10.1063/1.4821915 24089975

[brv70086-bib-0047] Bianco, S. , Ignaccolo, M. , Rider, M. S. , Ross, M. J. , Winsor, P. & Grigolini, P. (2007). Brain, music, and non‐Poisson renewal processes. Physical Review E 75, 61911. 10.1103/PhysRevE.75.061911 17677304

[brv70086-bib-0048] Biswal, B. B. , Mennes, M. , Zuo, X. N. , Gohel, S. , Kelly, C. , Smith, S. M. , Beckmann, C. F. , Adelstein, J. S. , Buckner, R. L. , Colcombe, S. & Dogonowski, A. M. (2010). Toward discovery science of human brain function. Proceedings of the National Academy of Sciences of the United States of America 107, 4734–4739. 20176931 10.1073/pnas.0911855107 PMC2842060

[brv70086-bib-0049] Biswas, T. & Fitzgerald, J. E. (2022). Geometric framework to predict structure from function in neural networks. Physical Review Research 4, 23255. 10.1103/physrevresearch.4.023255 PMC10456994 37635906

[brv70086-bib-0050] Block, F. , Dihne, M. & Loos, M. (2005). Inflammation in areas of remote changes following focal brain lesion. Progress in Neurobiology 75, 34–365. 10.1016/j.pneurobio.2005.03.004 15925027

[brv70086-bib-0051] Boccaletti, S. , Grebogi, C. , Lai, Y. C. , Mancini, H. & Maza, D. (2000). The control of chaos: theory and applications. Physics Reports 329, 103–197.

[brv70086-bib-0052] Boccaletti, S. , Latora, V. , Moreno, Y. , Chavez, M. & Hwang, D. U. (2006). Complex networks: structure and dynamics. Physics Reports 424, 175–308.

[brv70086-bib-0053] Boguñá, M. , Bonamassa, I. , De Domenico, M. , Havlin, S. , Krioukov, D. & Serrano, M. Á. (2021). Network geometry. Nature Reviews Physics 3, 114–135.

[brv70086-bib-0054] Bollobás, B. (1986). Combinatorics: Set Systems, Hypergraphs, Families of Vectors, and Combinatorial Probability. Cambridge University Press, Cambridge, UK.

[brv70086-bib-0055] Bonilha, L. , Kobayashi, E. , Rorden, C. , Cendes, F. & Li, L. M. (2003). Medial temporal lobe atrophy in patients with refractory temporal lobe epilepsy. Journal of Neurology, Neurosurgery and Psychiatry 74, 1627–1630. 14638879 10.1136/jnnp.74.12.1627 PMC1757422

[brv70086-bib-0056] Borsboom, D. & Cramer, A. O. (2013). Network analysis: an integrative approach to the structure of psychopathology. Annual Review of Clinical Psychology 9, 91–121. 10.1146/annurev-clinpsy-050212-185608 23537483

[brv70086-bib-0057] Bota, M. , Dong, H. W. & Swanson, L. W. (2003). From gene networks to brain networks. Nature Neuroscience 6, 795–799. 12886225 10.1038/nn1096

[brv70086-bib-0058] Botero, C. A. , Weissing, F. J. , Wright, J. & Rubenstein, D. R. (2015). Evolutionary tipping points in the capacity to adapt to environmental change. Proceedings of the National Academy of Sciences of the United States of America 112, 184–189. 25422451 10.1073/pnas.1408589111 PMC4291647

[brv70086-bib-0059] Bowick, M. J. , Fakhri, N. , Marchetti, M. C. & Ramaswamy, S. (2022). Symmetry, thermodynamics, and topology in active matter. Physical Review X 12, 10501.

[brv70086-bib-0060] Braak, H. & Braak, E. (1991). Neuropathological stageing of Alzheimer‐related changes. Acta Neuropathologica 82, 239–259. 1759558 10.1007/BF00308809

[brv70086-bib-0061] Braak, H. , Del Tredici, K. , Rüb, U. , De Vos, R. A. , Steur, E. N. J. & Braak, E. (2003). Staging of brain pathology related to sporadic Parkinson's disease. Neurobiology of Aging 24, 197–211. 12498954 10.1016/s0197-4580(02)00065-9

[brv70086-bib-0062] Braak, H. , Del Tredici, K. , Schultz, C. & Braak, E. V. A. (2000). Vulnerability of select neuronal types to Alzheimer's disease. Annals of the New York Academy of Sciences 924, 53–61. 11193802 10.1111/j.1749-6632.2000.tb05560.x

[brv70086-bib-0063] Bragin, A. , Wilson, C. L. & Engel, J. (2000). Chronic epileptogenesis requires development of a network of pathologically interconnected neuron clusters: a hypothesis. Epilepsia 41, S144–S152. 10999536 10.1111/j.1528-1157.2000.tb01573.x

[brv70086-bib-0064] Braunstein, A. , Dall'Asta, L. , Semerjian, G. & Zdeborová, L. (2016). Network dismantling. Proceedings of the National Academy of Sciences of the United States of America 113, 12368–12373. 27791075 10.1073/pnas.1605083113 PMC5098660

[brv70086-bib-0065] Brockmann, D. & Helbing, D. (2013). The hidden geometry of complex, network‐driven contagion phenomena. Science 342, 1337–1342. 24337289 10.1126/science.1245200

[brv70086-bib-0066] Bruining, H. , Hardstone, R. , Juarez‐Martinez, E. L. , Sprengers, J. , Avramiea, A. E. , Simpraga, S. , Houtman, S. J. , Poil, S. S. , Dallares, E. , Palva, S. & Oranje, B. (2020). Measurement of excitation‐inhibition ratio in autism spectrum disorder using critical brain dynamics. Scientific Reports 10, 9195. 32513931 10.1038/s41598-020-65500-4 PMC7280527

[brv70086-bib-0067] Brundin, P. , Melki, R. & Kopito, R. (2010). Prion‐like transmission of protein aggregates in neurodegenerative diseases. Nature Reviews Molecular Cell Biology 11, 301–307. 20308987 10.1038/nrm2873 PMC2892479

[brv70086-bib-0068] Buckner, R. L. (2004). Memory and executive function in aging and AD: multiple factors that cause decline and reserve factors that compensate. Neuron 44, 195–208. 15450170 10.1016/j.neuron.2004.09.006

[brv70086-bib-0069] Buckner, R. L. , Snyder, A. Z. , Shannon, B. J. , LaRossa, G. , Sachs, R. , Fotenos, A. F. , Sheline, Y. I. , Klunk, W. E. , Mathis, C. A. , Morris, J. C. & Mintun, M. A. (2005). Molecular, structural, and functional characterization of Alzheimer's disease: evidence for a relationship between default activity, amyloid, and memory. Journal of Neuroscience 25, 7709–7717. 16120771 10.1523/JNEUROSCI.2177-05.2005 PMC6725245

[brv70086-bib-0070] Buldú, J. M. , Bajo, R. , Maestú, F. , Castellanos, N. , Leyva, I. , Gil, P. , Sendiña‐Nadal, I. , Almendral, J. A. , Nevado, A. , del‐Pozo, F. & Boccaletti, S. (2011). Reorganization of functional networks in mild cognitive impairment. PLoS One 6, e19584. 21625430 10.1371/journal.pone.0019584 PMC3100302

[brv70086-bib-0071] Buldú, J. M. , Sendiña‐Nadal, I. , Leyva, I. , Echegoyen, I. , Martínez, J. H. & Papo, D. (2024). Neurodegeneration through the lens of network neuroscience. In An Insight into Neuromodulation: Current Trends and Future Challenges, pp. 241–271. Nova Science Publishers, New York, NY.

[brv70086-bib-0072] Buldú, J. M. , Sevilla‐Escoboza, R. , Aguirre, J. , Papo, D. & Gutiérrez, R. (2016). Interconnecting networks: the role of connector links. In Interconnected Networks, pp. 61–77. Springer International Publishing, Cham.

[brv70086-bib-0073] Buldyrev, S. V. , Parshani, R. , Paul, G. , Stanley, H. E. & Havlin, S. (2010). Catastrophic cascade of failures in interdependent networks. Nature 464, 1025–1028. 20393559 10.1038/nature08932

[brv70086-bib-0074] Bullmore, E. & Sporns, O. (2009). Complex brain networks: graph theoretical analysis of structural functional systems. Nature Reviews Neuroscience 10, 186–198. 19190637 10.1038/nrn2575

[brv70086-bib-0075] Burioni, R. , Casartelli, M. , Di Volo, M. , Livi, R. & Vezzani, A. (2014). Average synaptic activity and neural networks topology: a global inverse problem. Scientific Reports 4, 4336. 24613973 10.1038/srep04336 PMC3949294

[brv70086-bib-0076] Burns, S. P. , Santaniello, S. , Yaffe, R. B. , Jouny, C. C. , Crone, N. E. , Bergey, G. K. , Anderson, W. S. & Sarma, S. V. (2014). Network dynamics of the brain and influence of the epileptic seizure onset zone. Proceedings of the National Academy of Sciences of the United States of America 111, E5321–E5330. 25404339 10.1073/pnas.1401752111 PMC4267355

[brv70086-bib-0077] Burrows, D. R. , Diana, G. , Pimpel, B. , Moeller, F. , Richardson, M. P. , Bassett, D. S. , Meyer, M. P. & Rosch, R. E. (2023). Microscale neuronal activity collectively drives chaotic and inflexible dynamics at the macroscale in seizures. Journal of Neuroscience 43, 259–3283. 10.1523/JNEUROSCI.0171-22.2023 PMC7614507 37019622

[brv70086-bib-0078] Cabral, J. , Castaldo, F. , Vohryzek, J. , Litvak, V. , Bick, C. , Lambiotte, R. , Friston, K. , Kringelbach, M. L. & Deco, G. (2022). Metastable oscillatory modes emerge from synchronization in the brain spacetime connectome. Communications Physics 5, 184. 38288392 10.1038/s42005-022-00950-y PMC7615562

[brv70086-bib-0079] Cabral, J. , Hugues, E. , Sporns, O. & Deco, G. (2011). Role of local network oscillations in resting‐state functional connectivity. NeuroImage 57, 130–139. 21511044 10.1016/j.neuroimage.2011.04.010

[brv70086-bib-0080] Calhoun, V. D. , Miller, R. , Pearlson, G. & Adalı, T. (2014). The chronnectome: time‐varying connectivity networks as the next frontier in fMRI data discovery. Neuron 84, 262–274. 25374354 10.1016/j.neuron.2014.10.015 PMC4372723

[brv70086-bib-0081] Campa, A. , Dauxois, T. & Ruffo, S. (2009). Statistical mechanics and dynamics of solvable models with long‐range interactions. Physics Reports 480, 57–159.

[brv70086-bib-0082] Canguilhem, G. (1966). Le normal et le pathologique. Presses Universitaires de France, Paris.

[brv70086-bib-0083] Caprioglio, E. & Berthouze, L. (2024). Emergence of metastability in frustrated oscillatory networks: the key role of hierarchical modularity. Frontiers in Network Physiology 4, 1436046. 39233777 10.3389/fnetp.2024.1436046 PMC11372895

[brv70086-bib-0084] Carlson, J. M. & Doyle, J. (1999). Highly optimized tolerance: a mechanism for power laws in designed systems. Physical Review E 60, 1412–1427. 10.1103/physreve.60.1412 11969901

[brv70086-bib-0085] Carlson, J. M. & Doyle, J. (2002). Complexity and robustness. Proceedings of the National Academy of Sciences of the United States of America 99(Suppl. 1), 2538–2545. 11875207 10.1073/pnas.012582499 PMC128573

[brv70086-bib-0086] Casanova, M. & Trippe, J. (2009). Radial cytoarchitecture and patterns of cortical connectivity in autism. Philosophical Transactions of the Royal Society B 364, 1433–1436. 10.1098/rstb.2008.0331 PMC2677589 19528027

[brv70086-bib-0087] Casetti, L. , Pettini, M. & Cohen, E. G. D. (2000). Geometric approach to Hamiltonian dynamics and statistical mechanics. Physics Reports 337, 237–341.

[brv70086-bib-0088] Castellanos, F. X. , Di Martino, A. , Craddock, R. C. , Mehta, A. D. & Milham, M. P. (2013). Clinical applications of the functional connectome. NeuroImage 80, 527–540. 23631991 10.1016/j.neuroimage.2013.04.083 PMC3809093

[brv70086-bib-0089] Cavagna, A. , Cristín, J. , Giardina, I. & Veca, M. (2024). From noise on the sites to noise on the links: discretizing the conserved Kardar‐Parisi‐Zhang equation in real space. Physical Review E 109, 64136. 10.1103/PhysRevE.109.064136 39020940

[brv70086-bib-0090] Chang, W. C. , Kudlacek, J. , Hlinka, J. , Chvojka, J. , Hadrava, M. , Kumpost, V. , Powell, A. D. , Janca, R. , Maturana, M. I. , Karoly, P. J. & Freestone, D. R. (2018). Loss of neuronal network resilience precedes seizures and determines the ictogenic nature of interictal synaptic perturbations. Nature Neuroscience 21, 1742–1752. 30482946 10.1038/s41593-018-0278-y PMC7617160

[brv70086-bib-0091] Chantraine, P. (1968). Dictionnarie étymologique de la langue grecque. In Histoire des mots. Librairie Klincksieck, Paris.

[brv70086-bib-0092] Chavez, M. , Valencia, M. , Navarro, V. , Latora, V. & Martinerie, J. (2010). Functional modularity of background activities in normal and epileptic brain networks. Physical Review Letters 104, 118701. 20366507 10.1103/PhysRevLett.104.118701

[brv70086-bib-0093] Chen, L. , Liu, R. , Liu, Z. P. , Li, M. & Aihara, K. (2012). Detecting early‐warning signals for sudden deterioration of complex diseases by dynamical network biomarkers. Scientific Reports 2, 342. 22461973 10.1038/srep00342 PMC3314989

[brv70086-bib-0094] Chiang, S. & Haneef, Z. (2014). Graph theory findings in the pathophysiology of temporal lobe epilepsy. Clinical Neurophysiology 125, 1295–1305. 24831083 10.1016/j.clinph.2014.04.004 PMC4281254

[brv70086-bib-0095] Ciliberti, S. , Martin, O. C. & Wagner, A. (2007). Innovation and robustness in complex regulatory gene networks. Proceedings of the National Academy of Sciences of the United States of America 104, 13591–13596. 17690244 10.1073/pnas.0705396104 PMC1959426

[brv70086-bib-0096] Clark, D. G. & Abbott, L. F. (2024). Theory of coupled neuronal‐synaptic dynamics. Physical Review X 14, 21001.

[brv70086-bib-0097] Clavaguera, F. , Hench, J. , Goedert, M. & Tolnay, M. (2015). Invited review: prion‐like transmission and spreading of tau pathology. Neuropathology and Applied Neurobiology 41, 7–58. 10.1111/nan.12197 25399729

[brv70086-bib-0098] Cohen, R. & Havlin, S. (2010). Complex Networks: Structure, Robustness and Function. Cambridge University Press, Cambridge.

[brv70086-bib-0099] Cohen, R. , Erez, K. , Ben‐Avraham, D. & Havlin, S. (2000). Resilience of the internet to random breakdowns. Physical Review Letters 85, 4626–4628. 11082612 10.1103/PhysRevLett.85.4626

[brv70086-bib-0100] Cornelius, S. P. , Kath, W. L. & Motter, A. E. (2013). Realistic control of network dynamics. Nature Communications 4, 1942. 10.1038/ncomms2939 PMC3955710 23803966

[brv70086-bib-0101] Corominas‐Murtra, B. , Goñi, J. , Solé, R. V. & Rodríguez‐Caso, C. (2013). On the origins of hierarchy in complex networks. Proceedings of the National Academy of Sciences of the United States of America 110, 13316–13321. 23898177 10.1073/pnas.1300832110 PMC3746874

[brv70086-bib-0102] Cristino, A. S. , Williams, S. M. , Hawi, Z. , An, J. Y. , Bellgrove, M. A. , Schwartz, C. E. , Costa, L. D. F. & Claudianos, C. (2014). Neurodevelopmental and neuropsychiatric disorders represent an interconnected molecular system. Molecular Psychiatry 19, 294–301. 23439483 10.1038/mp.2013.16

[brv70086-bib-0103] Csermely, P. (2004). Strong links are important, but weak links stabilize them. Trends in Biochemical Sciences 29, 331–334. 15236738 10.1016/j.tibs.2004.05.004

[brv70086-bib-0104] Csete, M. & Doyle, J. (2002). Reverse engineering of biological complexity. Science 295, 1664–1669. 11872830 10.1126/science.1069981

[brv70086-bib-0105] Csete, M. & Doyle, J. (2004). Bow ties, metabolism and disease. Trends in Biotechnology 22, 446–450. 15331224 10.1016/j.tibtech.2004.07.007

[brv70086-bib-0106] Curto, C. (2017). What can topology tell us about the neural code? Bulletin of the American Mathematical Society 54, 63–78.

[brv70086-bib-0107] Curto, C. , Gross, E. , Jeffries, J. , Morrison, K. , Omar, M. , Rosen, Z. , Shiu, A. & Youngs, N. (2017). What makes a neural code convex? SIAM Journal on Applied Algebra and Geometry 1, 222–238.

[brv70086-bib-0108] Dai, L. , Korolev, K. S. & Gore, J. (2013). Slower recovery in space before collapse of connected populations. Nature 496, 355–358. 23575630 10.1038/nature12071 PMC4303252

[brv70086-bib-0109] Dai, L. , Vorselen, D. , Korolev, K. S. & Gore, J. (2012). Generic indicators for loss of resilience before a tipping point leading to population collapse. Science 336, 1175–1177. 22654061 10.1126/science.1219805

[brv70086-bib-0110] Daido, H. & Nakanishi, K. (2004). Aging transition and universal scaling in oscillator networks. Physical Review Letters 93, 104101. 15447406 10.1103/PhysRevLett.93.104101

[brv70086-bib-0111] Daido, H. & Nakanishi, K. (2007). Aging and clustering in globally coupled oscillators. Physical Review E 75, 056206. 10.1103/PhysRevE.75.056206 17677147

[brv70086-bib-0112] Daniels, B. C. , Chen, Y. J. , Sethna, J. P. , Gutenkunst, R. N. & Myers, C. R. (2008). Sloppiness, robustness, and evolvability in systems biology. Current Opinion in Biotechnology 19, 389–395. 18620054 10.1016/j.copbio.2008.06.008

[brv70086-bib-0113] Danziger, M. M. , Bonamassa, I. , Boccaletti, S. & Havlin, S. (2019). Dynamic interdependence and competition in multilayer networks. Nature Physics 15, 178–185.

[brv70086-bib-0114] David, A. S. (1994). Dysmodularity: a neurocognitive model for schizophrenia. Schizophrenia Bulletin 20, 249–255. 8085128 10.1093/schbul/20.2.249

[brv70086-bib-0115] Davis, G. W. (2006). Homeostatic control of neural activity: from phenomenology to molecular design. Annual Review of Neuroscience 29, 307–323. 10.1146/annurev.neuro.28.061604.135751 16776588

[brv70086-bib-0116] de Amorim Filho, E. C. , Moreira, R. A. & Santos, F. A. N. (2022). The Euler characteristic and topological phase transitions in complex systems. Journal of Physics: Complexity 3, 25003.

[brv70086-bib-0117] De Arcangelis, L. , Perrone‐Capano, C. & Herrmann, H. J. (2006). Self‐organized criticality model for brain plasticity. Physical Review Letters 96, 028107. 16486652 10.1103/PhysRevLett.96.028107

[brv70086-bib-0118] DeDeo, S. & Krakauer, D. C. (2012). Dynamics and processing in finite self‐similar networks. Journal of the Royal Society Interface 9, 2131–2144. 22378750 10.1098/rsif.2011.0840 PMC3405736

[brv70086-bib-0119] De Domenico, M. (2017). Diffusion geometry unravels the emergence of functional clusters in collective phenomena. Physical Review Letters 118, 168301. 28474920 10.1103/PhysRevLett.118.168301

[brv70086-bib-0120] De Domenico, M. & Biamonte, J. (2016). Spectral entropies as informational‐theoretic tools for complex networks comparison. Physical Review X 6, 041062.

[brv70086-bib-0121] de Haan, W. , van der Flier, W. M. , Koene, T. , Smits, L. L. , Scheltens, P. & Stam, C. J. (2012). Disrupted modular brain dynamics reflect cognitive dysfunction in Alzheimer's disease. NeuroImage 59, 3085–3093. 22154957 10.1016/j.neuroimage.2011.11.055

[brv70086-bib-0122] de Haan, W. , van Straaten, E. C. , Gouw, A. A. & Stam, C. J. (2017). Altering neuronal excitability to preserve network connectivity in a computational model of Alzheimer's disease. PLoS Computational Biology 13, e1005707. 28938009 10.1371/journal.pcbi.1005707 PMC5627940

[brv70086-bib-0123] Dehmamy, N. , Milanlouei, S. & Barabási, A. L. (2018). A structural transition in physical networks. Nature 563, 676–680. 30487615 10.1038/s41586-018-0726-6 PMC6637946

[brv70086-bib-0124] DeLisi, L. E. , Sakuma, M. , Tew, W. , Kushner, M. , Hoff, A. L. & Grimson, R. (1997). Schizophrenia as a chronic active brain process: a study of progressive brain structural change subsequent to the onset of schizophrenia. Psychiatry Research: Neuroimaging 74, 129–140. 10.1016/s0925-4927(97)00012-7 9255858

[brv70086-bib-0125] Del Tredici, K. & Braak, H. (2020). To stage, or not to stage. Current Opinion in Neurobiology 61, 10–22. 31862625 10.1016/j.conb.2019.11.008

[brv70086-bib-0126] Demetrius, L. A. (2013). Boltzmann, Darwin and directionality theory. Physics Reports 530, 1–85.

[brv70086-bib-0127] Demongeot, J. , Goles, E. , Morvan, M. , Noual, M. & Sené, S. (2010). Attraction basins as gauges of robustness against boundary conditions in biological complex systems. PLoS One 5, e11793. 20700525 10.1371/journal.pone.0011793 PMC2916819

[brv70086-bib-0128] De Pittà, M. , Brunel, N. & Volterra, A. (2016). Astrocytes: orchestrating synaptic plasticity? Neuroscience 323, 43–61. 25862587 10.1016/j.neuroscience.2015.04.001

[brv70086-bib-0129] Derényi, I. , Farkas, I. , Palla, G. & Vicsek, T. (2004). Topological phase transitions of random networks. Physica A 334, 583–590. 10.1103/PhysRevE.69.046117 15169079

[brv70086-bib-0130] Derényi, I. , Palla, G. & Vicsek, T. (2005). Clique percolation in random networks. Physical Review Letters 94, 160202. 15904198 10.1103/PhysRevLett.94.160202

[brv70086-bib-0131] DeVille, L. & Lerman, E. (2015). Modular dynamical systems on networks. Journal of The European Mathematical Society 17(2977), 3013.

[brv70086-bib-0132] De Vivo, L. , Bellesi, M. , Marshall, W. , Bushong, E. A. , Ellisman, M. H. , Tononi, G. & Cirelli, C. (2017). Ultrastructural evidence for synaptic scaling across the wake/sleep cycle. Science 355, 507–510. 28154076 10.1126/science.aah5982 PMC5313037

[brv70086-bib-0133] Dickinson, A. , Jones, M. & Milne, E. (2016). Measuring neural excitation and inhibition in autism: different approaches, different findings and different interpretations. Brain Research 1648, 277–289. 27421181 10.1016/j.brainres.2016.07.011

[brv70086-bib-0134] Dineen, R. A. , Vilisaar, J. , Hlinka, J. , Bradshaw, C. M. , Morgan, P. S. , Constantinescu, C. S. & Auer, D. P. (2009). Disconnection as a mechanism for cognitive dysfunction in multiple sclerosis. Brain 132, 239–249. 18953055 10.1093/brain/awn275

[brv70086-bib-0135] Do, A. L. & Gross, T. (2012). Self‐Organization in Continuous Adaptive Networks. River Publishers, New York, NY.

[brv70086-bib-0136] Dobson, T. , Malnič, A. & Marušič, D. (2022). Symmetry in Graphs, Edition (Volume 198). Cambridge University Press, Cambridge, UK.

[brv70086-bib-0137] Dogali, M. , Devinsky, O. , Luciano, D. , Perrine, K. & Beric, A. (1993). Multiple subpial cortical transections for the control of intractable epilepsy in exquisite cortex. Acta Neurochirurgica Supplement 58, 198–200. 10.1007/978-3-7091-9297-9_47 8109292

[brv70086-bib-0138] Dorogovtsev, S. N. , Goltsev, A. V. & Mendes, J. F. F. (2006). *k*‐Core organization of complex networks. Physical Review Letters 96, 040601. 16486798 10.1103/PhysRevLett.96.040601

[brv70086-bib-0139] Dorogovtsev, S. N. , Goltsev, A. V. & Mendes, J. F. F. (2008). Critical phenomena in complex networks. Reviews of Modern Physics 80, 1275–1335.

[brv70086-bib-0140] Douw, L. , van Dellen, E. , Gouw, A. A. , Griffa, A. , de Haan, W. , van den Heuvel, M. , Hillebrand, A. , Van Mieghem, P. , Nissen, I. A. , Otte, W. M. & Reijmer, Y. D. (2019). The road ahead in clinical network neuroscience. Network Neuroscience 3, 969–993. 31637334 10.1162/netn_a_00103 PMC6777944

[brv70086-bib-0141] Doyle, J. C. & Csete, M. (2005). Motifs, control, and stability. PLoS Biology 3, e392. 16277557 10.1371/journal.pbio.0030392 PMC1283396

[brv70086-bib-0142] Doyle, J. C. & Csete, M. (2011). Architecture, constraints, and behavior. Proceedings of the National Academy of Sciences of the United States of America 108, 15624–15630. 21788505 10.1073/pnas.1103557108 PMC3176601

[brv70086-bib-0143] Drzezga, A. , Becker, J. A. , Van Dijk, K. R. , Sreenivasan, A. , Talukdar, T. , Sullivan, C. , Schultz, A. P. , Sepulcre, J. , Putcha, D. , Greve, D. & Johnson, K. A. (2011). Neuronal dysfunction and disconnection of cortical hubs in non‐demented subjects with elevated amyloid burden. Brain 134, 1635–1646. 21490054 10.1093/brain/awr066 PMC3102239

[brv70086-bib-0144] Dyhrfjeld‐Johnsen, J. , Santhakumar, V. , Morgan, R. J. , Huerta, R. , Tsimring, L. & Soltesz, I. (2007). Topological determinants of epileptogenesis in large‐scale structural and functional models of the dentate gyrus derived from experimental data. Journal of Neurophysiology 97, 1566–1587. 17093119 10.1152/jn.00950.2006

[brv70086-bib-0145] Edelman, G. M. (1987). Neural Darwinism: The Theory of Neuronal Group Selection. Basic Books, New York. 10.1126/science.240.4860.1802 17842436

[brv70086-bib-0146] Edelman, G. M. & Gally, J. A. (2001). Degeneracy and complexity in biological systems. Proceedings of the National Academy of Sciences of the United States of America 98, 13763–13768. 11698650 10.1073/pnas.231499798 PMC61115

[brv70086-bib-0147] Eichler, S. A. & Meier, J. C. (2008). E‐I balance and human diseases ‐ from molecules to networking. Frontiers in Molecular Neuroscience 1, 2. 18946535 10.3389/neuro.02.002.2008 PMC2526001

[brv70086-bib-0148] Engel, J. Jr. , Thompson, P. M. , Stern, J. M. , Staba, R. J. , Bragin, A. & Mody, I. (2013). Connectomics and epilepsy. Current Opinion in Neurology 26, 186–194. 23406911 10.1097/WCO.0b013e32835ee5b8 PMC4064674

[brv70086-bib-0149] Epskamp, S. , Borsboom, D. & Fried, E. I. (2018). Estimating psychological networks and their accuracy: a tutorial paper. Behavior Research Methods 50, 195–212. 28342071 10.3758/s13428-017-0862-1 PMC5809547

[brv70086-bib-0150] Erickson, M. J. (2014). Introduction to combinatorics. In Discrete Mathematics and Optimization, Second Edition (Volume 78). John Wiley & Sons, Hoboken, NJ.

[brv70086-bib-0151] Eser, R. A. , Ehrenberg, A. J. , Petersen, C. , Dunlop, S. , Mejia, M. B. , Suemoto, C. K. , Walsh, C. M. , Rajana, H. , Oh, J. , Theofilas, P. & Seeley, W. W. (2018). Selective vulnerability of brainstem nuclei in distinct tauopathies: a postmortem study. Journal of Neuropathology & Experimental Neurology 77, 149–161. 29304218 10.1093/jnen/nlx113 PMC6251636

[brv70086-bib-0152] Estévez‐Priego, E. , Teller, S. , Granell, C. , Arenas, A. & Soriano, J. (2020). Functional strengthening through synaptic scaling upon connectivity disruption in neuronal cultures. Network Neuroscience 4, 1160–1180. 33409434 10.1162/netn_a_00156 PMC7781611

[brv70086-bib-0153] Faci‐Lázaro, S. , Lor, T. , Ródenas, G. , Mazo, J. J. , Soriano, J. & Gómez‐Gardeñes, J. (2022). Dynamical robustness of collective neuronal activity upon targeted damage in interdependent networks. The European Physical Journal Special Topics 231, 195–201.

[brv70086-bib-0154] Farooq, H. , Chen, Y. , Georgiou, T. T. , Tannenbaum, A. & Lenglet, C. (2019). Network curvature as a hallmark of brain structural connectivity. Nature Communications 10, 4937. 10.1038/s41467-019-12915-x PMC6821808 31666510

[brv70086-bib-0155] Fisher, L. (2015). More than 70 ways to show resilience. Nature 518, 35. 10.1038/518035a 25652986

[brv70086-bib-0156] Flanagan, R. , Lacasa, L. , Towlson, E. K. , Lee, S. H. & Porter, M. A. (2019). Effect of antipsychotics on community structure in functional brain networks. Journal of Complex Networks 7, 932–960.

[brv70086-bib-0157] Foffani, G. & Obeso, J. A. (2018). A cortical pathogenic theory of Parkinson's disease. Neuron 99, 1116–1128. 30236282 10.1016/j.neuron.2018.07.028

[brv70086-bib-0158] Fong, M.‐f. , Newman, J. P. , Potter, S. M. & Wenner, P. (2015). Upward synaptic scaling is dependent on neurotransmission rather than spiking. Nature Communications 6, 6339. 10.1038/ncomms7339 PMC4355957 25751516

[brv70086-bib-0159] Fornito, A. , Zalesky, A. & Breakspear, M. (2015). The connectomics of brain disorders. Nature Reviews Neuroscience 16, 159–172. 25697159 10.1038/nrn3901

[brv70086-bib-0160] Fornito, A. , Zalesky, A. , Pantelis, C. & Bullmore, E. T. (2012). Schizophrenia, neuroimaging and connectomics. NeuroImage 62, 2296–2314. 22387165 10.1016/j.neuroimage.2011.12.090

[brv70086-bib-0161] Franzosi, R. & Pettini, M. (2004). Theorem on the origin of phase transitions. Physical Review Letters 92, 060601. 14995226 10.1103/PhysRevLett.92.060601

[brv70086-bib-0162] Fries, P. (2005). A mechanism for cognitive dynamics: neuronal communication through neuronal coherence. Trends in Cognitive Sciences 9, 474–480. 16150631 10.1016/j.tics.2005.08.011

[brv70086-bib-0163] Friston, K. , Brown, H. R. , Siemerkus, J. & Stephan, K. E. (2016). The dysconnection hypothesis (2016). Schizophrenia Research 176, 83–94. 27450778 10.1016/j.schres.2016.07.014 PMC5147460

[brv70086-bib-0164] Friston, K. J. (1998). The disconnection hypothesis. Schizophrenia Research 30, 115–125. 9549774 10.1016/s0920-9964(97)00140-0

[brv70086-bib-0165] Friston, K. J. & Frith, C. D. (1995). Schizophrenia: a disconnection syndrome. Clinical Neuroscience 3, 89–97. 7583624

[brv70086-bib-0166] Frith, C. D. (1992). The Cognitive Neuropsychology of Schizophrenia. Psychology Press, London.

[brv70086-bib-0167] Fülöp, T. , Desroches, M. , Cohen, A. A. , Santos, F. A. N. & Rodrigues, S. (2020). Why we should use topological data analysis in ageing: towards defining the “topological shape of ageing”. Mechanisms of Ageing and Development 192, 111390. 33127442 10.1016/j.mad.2020.111390

[brv70086-bib-0168] Gallos, L. K. , Makse, H. A. & Sigman, M. (2012). A small world of weak ties provides optimal global integration of self‐similar modules in functional brain networks. Proceedings of the National Academy of Sciences of the United States of America 109, 2825–2830. 22308319 10.1073/pnas.1106612109 PMC3286928

[brv70086-bib-0169] Gallos, L. K. , Song, C. , Havlin, S. & Makse, H. A. (2007). Scaling theory of transport in complex biological networks. Proceedings of the National Academy of Sciences of the United States of America 104, 7746–7751. 17470793 10.1073/pnas.0700250104 PMC1876518

[brv70086-bib-0170] Ganmor, E. , Segev, R. & Schneidman, E. (2015). A thesaurus for a neural population code. eLife 4, e06134. 26347983 10.7554/eLife.06134 PMC4562117

[brv70086-bib-0171] Gao, J. , Buldyrev, S. V. , Havlin, S. & Stanley, H. E. (2011). Robustness of a network of networks. Physical Review Letters 107, 195701. 22181627 10.1103/PhysRevLett.107.195701

[brv70086-bib-0172] Gao, J. , Buldyrev, S. V. , Stanley, H. E. & Havlin, S. (2012). Networks formed from interdependent networks. Nature Physics 8, 40–48. 10.1103/PhysRevE.85.066134 23005189

[brv70086-bib-0173] Gao, J. , Liu, X. , Li, D. & Havlin, S. (2015). Recent progress on the resilience of complex networks. Energies 8, 12187–12210.

[brv70086-bib-0174] Gao, P. & Ganguli, S. (2015). On simplicity and complexity in the brave new world of large‐scale neuroscience. Current Opinion in Neurobiology 32, 148–155. 25932978 10.1016/j.conb.2015.04.003

[brv70086-bib-0175] Garlaschelli, D. , Ruzzenenti, F. & Basosi, R. (2010). Complex networks and symmetry I: a review. Symmetry 2, 1683–1709.

[brv70086-bib-0176] Geier, C. , Bialonski, S. , Elger, C. E. & Lehnertz, K. (2015). How important is the seizure onset zone for seizure dynamics? Seizure 25, 160–166. 25468511 10.1016/j.seizure.2014.10.013

[brv70086-bib-0177] Gentry, L. R. (1994). Imaging of closed head injury. Radiology 191, 1–17. 8134551 10.1148/radiology.191.1.8134551

[brv70086-bib-0178] Geschwind, D. H. & Levitt, P. (2007). Autism spectrum disorders: developmental disconnection syndromes. Current Opinion in Neurobiology 17, 103–111. 17275283 10.1016/j.conb.2007.01.009

[brv70086-bib-0179] Geschwind, N. (1965). Disconnexion syndromes in animals and man. Brain 88, 585. 5318824 10.1093/brain/88.3.585

[brv70086-bib-0180] Ghavasieh, A. , Bertagnolli, G. & De Domenico, M. (2023). Dismantling the information flow in complex interconnected systems. Physical Review Research 5, 013084.

[brv70086-bib-0181] Ghavasieh, A. & De Domenico, M. (2024). Diversity of information pathways drives sparsity in real‐world networks. Nature Physics 20, 512–519.

[brv70086-bib-0182] Ghrist, R. W. (2014). Elementary Applied Topology, Edition (Volume 1). Createspace, Seattle.

[brv70086-bib-0183] Giusti, C. & Itskov, V. (2014). A no‐go theorem for one‐layer feedforward networks. Neural Computation 26, 2527–2540. 25149704 10.1162/NECO_a_00657

[brv70086-bib-0184] Glass, L. (2015). Dynamical disease: challenges for nonlinear dynamics and medicine. Chaos 25, 097603. 26428556 10.1063/1.4915529

[brv70086-bib-0185] Glass, L. & Mackey, M. C. (1979). Pathological conditions resulting from instabilities in physiological control systems. Annals of the New York Academy of Sciences 316, 214–235. 288317 10.1111/j.1749-6632.1979.tb29471.x

[brv70086-bib-0186] Glass, L. & Mackey, M. C. (1988). From Clocks to Chaos: The Rhythms of Life. Princeton University Press.

[brv70086-bib-0187] Glasser, M. F. , Smith, S. M. , Marcus, D. S. , Andersson, J. L. , Auerbach, E. J. , Behrens, T. E. , Coalson, T. S. , Harms, M. P. , Jenkinson, M. , Moeller, S. & Robinson, E. C. (2016). The human connectome project's neuroimaging approach. Nature Neuroscience 19, 1175–1187. 27571196 10.1038/nn.4361 PMC6172654

[brv70086-bib-0188] Godwin, D. , Barry, R. L. & Marois, R. (2015). Breakdown of the brain's functional network modularity with awareness. Proceedings of the National Academy of Sciences of the United States of America 112, 3799–3804. 25759440 10.1073/pnas.1414466112 PMC4378398

[brv70086-bib-0189] Goh, K.‐I. , Cusick, M. E. , Valle, D. , Childs, B. , Vidal, M. & Barabási, A.‐L. (2007). The human disease network. Proceedings of the National Academy of Sciences of the United States of America 104, 8685–8690. 17502601 10.1073/pnas.0701361104 PMC1885563

[brv70086-bib-0190] Gollo, L. L. , Roberts, J. A. & Cocchi, L. (2017). Mapping how local perturbations influence systems‐level brain dynamics. NeuroImage 160, 97–112. 28126550 10.1016/j.neuroimage.2017.01.057

[brv70086-bib-0191] Golubitsky, M. & Wang, Y. (2020). Infinitesimal homeostasis in three‐node input–output networks. Journal of Mathematical Biology 80, 1163–1185. 31919651 10.1007/s00285-019-01457-x

[brv70086-bib-0192] Gonzalez, J. P. , Guiserix, M. , Sauvage, F. , Guitton, J. S. , Vidal, P. , Bahi‐Jaber, N. , Louzir, H. & Pontier, D. (2010). Pathocenosis: a holistic approach to disease ecology. EcoHealth 7, 237–241. 20593218 10.1007/s10393-010-0326-x PMC3005112

[brv70086-bib-0193] Greenbury, S. F. , Schaper, S. , Ahnert, S. E. & Louis, A. A. (2016). Genetic correlations greatly increase mutational robustness and can both reduce and enhance evolvability. PLoS Computational Biology 12, e1004773. 26937652 10.1371/journal.pcbi.1004773 PMC4777517

[brv70086-bib-0194] Greer, J. E. (2011). The Characterization of the Anterograde and Retrograde Consequences of Traumatic Axonal Injury in a Mouse Model of Diffuse Brain Injury. Virginia Commonwealth University, Richmond, VA.

[brv70086-bib-0195] Grilli, J. , Barabás, G. , Michalska‐Smith, M. J. & Allesina, S. (2017). Higher‐order interactions stabilize dynamics in competitive network models. Nature 548, 210–213. 28746307 10.1038/nature23273

[brv70086-bib-0196] Grmek, M. D. (1969). Préliminaire d'une étude historique des maladies. Annales ESC 24, 1437–1483.

[brv70086-bib-0197] Gross, T. & Blasius, B. (2008). Adaptive coevolutionary networks: a review. Journal of the Royal Society Interface 5, 259–271. 17971320 10.1098/rsif.2007.1229 PMC2405905

[brv70086-bib-0198] Gross, B. , Bonamassa, I. & Havlin, S. (2021). Interdependent transport via percolation backbones in spatial networks. Physica A 567, 125644.

[brv70086-bib-0199] Gross, B. , Bonamassa, I. & Havlin, S. (2023a). Dynamics of cascades in spatial interdependent networks. Chaos 33, 103116. 37831796 10.1063/5.0165796

[brv70086-bib-0200] Gross, B. , Havlin, S. & Barzel, B. (2023 *b*). Dense network motifs enhance dynamical stability. *arXiv:2304.12044*.

[brv70086-bib-0201] Gu, S. , Pasqualetti, F. , Cieslak, M. , Telesford, Q. K. , Yu, A. B. , Kahn, A. E. , Medaglia, J. D. , Vettel, J. M. , Miller, M. B. , Grafton, S. T. & Bassett, D. S. (2015). Controllability of structural brain networks. Nature Communications 6, 8414. 10.1038/ncomms9414 PMC4600713 26423222

[brv70086-bib-0202] Guloksuz, S. , Pries, L. K. & Van Os, J. (2017). Application of network methods for understanding mental disorders: pitfalls and promise. Psychological Medicine 47, 2743–2752. 28578740 10.1017/S0033291717001350

[brv70086-bib-0203] Gunderson, L. H. (2000). Ecological resilience—in theory and application. Annual Review of Ecology, Evolution, and Systematics 31, 425–439.

[brv70086-bib-0204] Gutiérrez, R. , Sendiña‐Nadal, I. , Zanin, M. , Papo, D. & Boccaletti, S. (2012). Targeting the dynamics of complex networks. Scientific Reports 2, 396. 22563525 10.1038/srep00396 PMC3343324

[brv70086-bib-0205] Hahamy, A. , Behrmann, M. & Malach, R. (2015). The idiosyncratic brain: distortion of spontaneous connectivity patterns in autism spectrum disorder. Nature Neuroscience 18, 302–309. 25599222 10.1038/nn.3919

[brv70086-bib-0206] Hallett, M. (2005). Neuroplasticity and rehabilitation. Journal of Rehabilitation Research and Development 42, R17. 16320136

[brv70086-bib-0207] Halu, A. , De Domenico, M. , Arenas, A. & Sharma, A. (2019). The multiplex network of human diseases. npj Systems Biology and Applications 5, 15. 31044086 10.1038/s41540-019-0092-5 PMC6478736

[brv70086-bib-0208] Hansen, J. Y. , Shafiei, G. , Markello, R. D. , Smart, K. , Cox, S. M. , Nørgaard, M. , Beliveau, V. , Wu, Y. , Gallezot, J. D. , Aumont, É. & Servaes, S. (2022). Mapping neurotransmitter systems to the structural and functional organization of the human neocortex. Nature Neuroscience 25, 1569–1581. 36303070 10.1038/s41593-022-01186-3 PMC9630096

[brv70086-bib-0209] Hasan, M. Z. & Kane, C. L. (2010). Colloquium: topological insulators. Reviews of Modern Physics 82, 3045–3067.

[brv70086-bib-0210] Hellyer, P. J. , Leech, R. , Ham, T. E. , Bonnelle, V. & Sharp, D. J. (2013). Individual prediction of white matter injury following traumatic brain injury. Annals of Neurology 73, 489–499. 23426980 10.1002/ana.23824

[brv70086-bib-0211] Henderson, J. A. & Robinson, P. A. (2011). Geometric effects on complex network structure in the cortex. Physical Review Letters 107, 018102. 21797575 10.1103/PhysRevLett.107.018102

[brv70086-bib-0212] Henderson, J. A. & Robinson, P. A. (2013). Using geometry to uncover relationships between isotropy, homogeneity, and modularity in cortical connectivity. Brain Connectivity 3, 423–437. 23802922 10.1089/brain.2013.0151

[brv70086-bib-0213] Henderson, J. A. & Robinson, P. A. (2014). Relations between the geometry of cortical gyrification and white‐matter network architecture. Brain Connectivity 4, 112–130. 24437717 10.1089/brain.2013.0183

[brv70086-bib-0214] Hennig, M. H. (2023). The sloppy relationship between neural circuit structure and function. The Journal of Physiology 601, 3025–3035. 35876720 10.1113/JP282757

[brv70086-bib-0215] Hens, C. , Harush, U. , Haber, S. , Cohen, R. & Barzel, B. (2019). Spatiotemporal signal propagation in complex networks. Nature Physics 15, 403–412.

[brv70086-bib-0216] Hilary, F. G. & Grafman, J. H. (2017). Injured brains and adaptive networks: the benefits and costs of hyperconnectivity. Trends in Cognitive Sciences 21, 385–401. 28372878 10.1016/j.tics.2017.03.003 PMC6664441

[brv70086-bib-0217] Holling, C. S. (1973). Resilience and stability of ecological systems. Annual Review of Ecology, Evolution, and Systematics 4, 1–23.

[brv70086-bib-0218] Holling, C. S. (1996). Engineering resilience versus ecological resilience. Eng. Ecol. Constraints 31, 32.

[brv70086-bib-0219] Holling, C. S. , Gunderson, L. H. & Peterson, G. D. (2002). Panarchy: Understanding Transformations in Human and Natural Systems, pp. 63–102. Island Press.

[brv70086-bib-0220] Hopfield, J. J. (1974). Kinetic proofreading: a new mechanism for reducing errors in biosynthetic processes requiring high specificity. Proceedings of the National Academy of Sciences of the United States of America 71, 4135–4139. 4530290 10.1073/pnas.71.10.4135 PMC434344

[brv70086-bib-0221] Howell, B. R. , Styner, M. A. , Gao, W. , Yap, P. T. , Wang, L. , Baluyot, K. , Yacoub, E. , Chen, G. , Potts, T. , Salzwedel, A. & Li, G. (2019). The UNC/UMN baby connectome project (BCP): an overview of the study design and protocol development. NeuroImage 185, 891–905. 29578031 10.1016/j.neuroimage.2018.03.049 PMC6151176

[brv70086-bib-0222] Hulkower, M. B. , Poliak, D. B. , Rosenbaum, S. B. , Zimmerman, M. E. & Lipton, M. L. (2013). A decade of DTI in traumatic brain injury: 10 years and 100 articles later. American Journal of Neuroradiology 34, 2064–2074. 23306011 10.3174/ajnr.A3395 PMC7964847

[brv70086-bib-0223] Hunt, M. J. , Kopell, N. J. , Traub, R. D. & Whittington, M. A. (2017). Aberrant network activity in schizophrenia. Trends in Neurosciences 40, 371–382. 28515010 10.1016/j.tins.2017.04.003 PMC5523137

[brv70086-bib-0224] Ibañez‐Marcelo, E. & Alarcon, T. (2014). The topology of robustness and evolvability in evolutionary systems with genotype–phenotype map. Journal of Theoretical Biology 356, 144–162. 24793533 10.1016/j.jtbi.2014.04.014

[brv70086-bib-0225] Iraji, A. , Deramus, T. P. , Lewis, N. , Yaesoubi, M. , Stephen, J. M. , Erhardt, E. , Belger, A. , Ford, J. M. , McEwen, S. , Mathalon, D. H. & Mueller, B. A. (2019). The spatial chronnectome reveals a dynamic interplay between functional segregation and integration. Human Brain Mapping 40, 3058–3077. 30884018 10.1002/hbm.24580 PMC6548674

[brv70086-bib-0226] Iturria‐Medina, Y. , Carbonell, F. M. , Sotero, R. C. , Chouinard‐Decorte, F. & Evans, A. C. (2017). Multifactorial causal model of brain (dis)organization and therapeutic intervention: application to Alzheimer's disease. NeuroImage 152, 60–77. 28257929 10.1016/j.neuroimage.2017.02.058

[brv70086-bib-0227] Iturria‐Medina, Y. , Sotero, R. C. , Toussaint, P. J. & Evans, A. C. (2014). Epidemic spreading model to characterize misfolded proteins propagation in aging and associated neurodegenerative disorders. PLoS Computational Biology 10, e1003956. 25412207 10.1371/journal.pcbi.1003956 PMC4238950

[brv70086-bib-0228] James, W. (1890). The Principles of Psychology. Henry Holt and Company, New York, NY, USA.

[brv70086-bib-0229] Jazayeri, M. & Ostojic, S. (2021). Interpreting neural computations by examining intrinsic and embedding dimensionality of neural activity. Current Opinion in Neurobiology 70, 113–120. 34537579 10.1016/j.conb.2021.08.002 PMC8688220

[brv70086-bib-0230] Jensen, K. T. , Kadmon Harpaz, N. , Dhawale, A. K. , Wolff, S. B. & Ölveczky, B. P. (2022). Long‐term stability of single neuron activity in the motor system. Nature Neuroscience 25, 1664–1674. 36357811 10.1038/s41593-022-01194-3 PMC11152193

[brv70086-bib-0231] Jirsa, V. , Sporns, O. , Breakspear, M. , Deco, G. & McIntosh, A. R. (2010). Towards the virtual brain: network modeling of the intact and the damaged brain. Archives Italiennes de Biologie 148, 189–205. 21175008

[brv70086-bib-0232] Jirsa, V. K. , Proix, T. , Perdikis, D. , Woodman, M. M. , Wang, H. , Gonzalez‐Martinez, J. , Bernard, C. , Bénar, C. , Guye, M. , Chauvel, P. & Bartolomei, F. (2017). The virtual epileptic patient: individualized whole‐brain models of epilepsy spread. NeuroImage 145, 377–388. 27477535 10.1016/j.neuroimage.2016.04.049

[brv70086-bib-0233] Johnson, G. W. , Doss, D. J. , Morgan, V. L. , Paulo, D. L. , Cai, L. Y. , Shless, J. S. , Negi, A. S. , Gummadavelli, A. , Kang, H. , Reddy, S. B. & Naftel, R. P. (2023). The interictal suppression hypothesis in focal epilepsy: network‐level supporting evidence. Brain 146, 2828–2845. 36722219 10.1093/brain/awad016 PMC10316780

[brv70086-bib-0234] Johnson, V. E. , Stewart, W. & Smith, D. H. (2013). Axonal pathology in traumatic brain injury. Experimental Neurology 246, 35–43. 22285252 10.1016/j.expneurol.2012.01.013 PMC3979341

[brv70086-bib-0235] Kaiser, F. , Ronellenfitsch, H. & Witthaut, D. (2020). Discontinuous transition to loop formation in optimal supply networks. Nature Communications 11, 5796. 10.1038/s41467-020-19567-2 PMC7670464 33199688

[brv70086-bib-0236] Kaiser, M. (2013). The potential of the human connectome as a biomarker of brain disease. Frontiers in Human Neuroscience 7, 484. 23966935 10.3389/fnhum.2013.00484 PMC3744009

[brv70086-bib-0237] Kálmán, R. E. (1963). Mathematical description of linear dynamical systems. Journal of the Society for Industrial and Applied Mathematics Series A Control 1, 152–192.

[brv70086-bib-0238] Kaneko, K. (2007). Evolution of robustness to noise and mutation in gene expression dynamics. PLoS One 2, e434. 17502916 10.1371/journal.pone.0000434 PMC1855988

[brv70086-bib-0239] Kashtan, N. & Alon, U. (2005). Spontaneous evolution of modularity and network motifs. Proceedings of the National Academy of Sciences of the United States of America 102, 13773–13778. 16174729 10.1073/pnas.0503610102 PMC1236541

[brv70086-bib-0240] Kastner, M. (2008). Phase transitions and configuration space topology. Reviews of Modern Physics 80, 167–187.

[brv70086-bib-0241] Kastner, M. & Schnetz, O. (2008). Phase transitions induced by saddle points of vanishing curvature. Physical Review Letters 100, 160601. 18518179 10.1103/PhysRevLett.100.160601

[brv70086-bib-0242] Katifori, E. , Szöllősi, G. J. & Magnasco, M. O. (2010). Damage and fluctuations induce loops in optimal transport networks. Physical Review Letters 104, 048704. 20366746 10.1103/PhysRevLett.104.048704

[brv70086-bib-0243] Kenett, D. Y. , Perc, M. & Boccaletti, S. (2015). Networks of networks–an introduction. Chaos, Solitons and Fractals 80, 1–6.

[brv70086-bib-0244] Khona, M. , Chandra, S. & Fiete, I. R. (2023). From smooth cortical gradients to discrete modules: spontaneous and topologically robust emergence of modularity in grid cells. bioRxiv. 10.1101/2021.10.28.466284.

[brv70086-bib-0245] Kiani, N. A. , Gomez‐Cabrero, D. & Bianconi, G. (eds) (2021). Networks of Networks in Biology: Concepts, Tools and Applications. Cambridge University Press.

[brv70086-bib-0246] Kim, J. Z. , Soffer, J. M. , Kahn, A. E. , Vettel, J. M. , Pasqualetti, F. & Bassett, D. S. (2018). Role of graph architecture in controlling dynamical networks with applications to neural systems. Nature Physics 14, 91–98. 29422941 10.1038/nphys4268 PMC5798649

[brv70086-bib-0247] Kitano, H. (2004 *a*). Biological robustness. Nature Reviews Genetics 5, 826–837. 10.1038/nrg1471 15520792

[brv70086-bib-0248] Kitano, H. (2004 *b*). Cancer as a robust system: implications for anticancer therapy. Nature Reviews. Cancer 4, 227–235. 14993904 10.1038/nrc1300

[brv70086-bib-0249] Kitano, H. (2007). Towards a theory of biological robustness. Molecular Systems Biology 3, 137. 17882156 10.1038/msb4100179 PMC2013924

[brv70086-bib-0250] Koetter, R. , Effros, M. & Médard, M. (2011). A theory of network equivalence — part I: point‐to‐point channels. IEEE Transactions on Information Theory 57, 972–995.

[brv70086-bib-0251] Koetter, R. & Médard, M. (2003). An algebraic approach to network coding. IEEE/ACM Transactions on Networking 11, 782–795.

[brv70086-bib-0252] Korhonen, O. , Zanin, M. & Papo, D. (2021). Principles and open questions in functional brain network reconstruction. Human Brain Mapping 42, 3680–3711. 34013636 10.1002/hbm.25462 PMC8249902

[brv70086-bib-0253] Kozma, R. & Freeman, W. J. (2016). Cognitive Phase Transitions in the Cerebral Cortex‐Enhancing the Neuron Doctrine by Modeling Neural Fields, Edition (Volume 39). Springer International Publishing, Switzerland.

[brv70086-bib-0254] Krakovská, H. , Kuehn, C. & Longo, I. P. (2024). Resilience of dynamical systems. European Journal of Applied Mathematics 35, 155–200.

[brv70086-bib-0255] Kramer, M. A. & Cash, S. S. (2012). Epilepsy as a disorder of cortical network organization. The Neuroscientist 18, 360–372. 22235060 10.1177/1073858411422754 PMC3736575

[brv70086-bib-0256] Kramer, M. A. , Kolaczyk, E. D. & Kirsch, H. E. (2008). Emergent network topology at seizure onset in humans. Epilepsy Research 79, 173–186. 18359200 10.1016/j.eplepsyres.2008.02.002

[brv70086-bib-0257] Kubota, M. , Miyata, J. , Sasamoto, A. , Sugihara, G. , Yoshida, H. , Kawada, R. , Fujimoto, S. , Tanaka, Y. , Sawamoto, N. , Fukuyama, H. & Takahashi, H. (2013). Thalamocortical disconnection in the orbitofrontal region associated with cortical thinning in schizophrenia. JAMA Psychiatry 70, 12–21. 22945538 10.1001/archgenpsychiatry.2012.1023

[brv70086-bib-0258] Kuceyeski, A. , Kamel, H. , Navi, B. B. , Raj, A. & Iadecola, C. (2014). Predicting future brain tissue loss from white matter connectivity disruption in ischemic stroke. Stroke 45, 717–722. 24523041 10.1161/STROKEAHA.113.003645 PMC3943489

[brv70086-bib-0259] Kuceyeski, A. , Zhang, Y. & Raj, A. (2012). Linking white matter integrity loss to associated cortical regions using structural connectivity information in Alzheimer's disease and fronto‐temporal dementia: the loss in connectivity (LoCo) score. NeuroImage 61, 1311–1323. 22484307 10.1016/j.neuroimage.2012.03.039 PMC3376902

[brv70086-bib-0260] Kuhnert, M. T. , Elger, C. E. & Lehnertz, K. (2010). Long‐term variability of global statistical properties of epileptic brain networks. Chaos 20, 043126. 21198096 10.1063/1.3504998

[brv70086-bib-0261] Lahav, N. , Ksherim, B. , Ben‐Simon, E. , Maron‐Katz, A. , Cohen, R. & Havlin, S. (2016). K‐shell decomposition reveals hierarchical cortical organization of the human brain. New Journal of Physics 18, 083013.

[brv70086-bib-0262] Lai, Y. C. (2014). Controlling complex, non‐ linear dynamical networks. National Science Review 1, 339–341.

[brv70086-bib-0263] Lalande, A. (1927). Vocabulaire technique et critique de la philosophie, revu par MM. les membres et correspondants de la Société française de philosophie et publié, avec leurs corrections et observations par André Lalande, membre de l'Institut, professeur à la Sorbonne, secrétaire général de la Société (2 volumes, 1927). Presses Universitaires de France.

[brv70086-bib-0264] Lambiotte, R. , Rosvall, M. & Scholtes, I. (2019). From networks to optimal higher‐order models of complex systems. Nature Physics 15, 313–320. 30956684 10.1038/s41567-019-0459-y PMC6445364

[brv70086-bib-0265] Landmann, S. , Baumgarten, L. & Bornholdt, S. (2021). Self‐organized criticality in neural networks from activity‐based rewiring. Physical Review E 103, 032304. 33862737 10.1103/PhysRevE.103.032304

[brv70086-bib-0266] Larson‐Prior, L. J. , Oostenveld, R. , Della Penna, S. , Michalareas, G. , Prior, F. , Babajani‐Feremi, A. , Schoffelen, J. M. , Marzetti, L. , De Pasquale, F. , Di Pompeo, F. , Stout, J. & WU‐Minn HCP Consortium (2013). Adding dynamics to the human connectome project with MEG. NeuroImage 80, 190–201. 23702419 10.1016/j.neuroimage.2013.05.056 PMC3784249

[brv70086-bib-0267] Latora, V. & Marchiori, M. (2001). Efficient behavior of small‐world networks. Physical Review Letters 87, 198701. 11690461 10.1103/PhysRevLett.87.198701

[brv70086-bib-0268] Le Carret, N. , Lafont, S. , Letenneur, L. , Dartigues, J. F. , Mayo, W. & Fabrigoule, C. (2003). The effect of education on cognitive performances and its implication for the constitution of the cognitive reserve. Developmental Neuropsychology 23, 317–337. 12740188 10.1207/S15326942DN2303_1

[brv70086-bib-0269] Lehnertz, K. , Bialonski, S. , Horstmann, M. T. , Krug, D. , Rothkegel, A. , Staniek, M. & Wagner, T. (2009). Synchronization phenomena in human epileptic brain networks. Journal of Neuroscience Methods 183, 42–48. 19481573 10.1016/j.jneumeth.2009.05.015

[brv70086-bib-0270] Lehnertz, K. , Bröhl, T. & von Wrede, R. (2023). Epileptic‐network‐based prediction and control of seizures in humans. Neurobiology of Disease 181, 106098. 36997129 10.1016/j.nbd.2023.106098

[brv70086-bib-0271] Lehnertz, K. & Dickten, H. (2015). Assessing directionality and strength of coupling through symbolic analysis: an application to epilepsy patients. Philosophical Transactions of the Royal Society A 373, 20140094. 10.1098/rsta.2014.0094 PMC4281866 25548267

[brv70086-bib-0272] Lehnertz, K. , Geier, C. , Rings, T. & Stahn, K. (2017). Capturing time‐varying brain dynamics. EPJ Nonlinear Biomedical Physics 5, 2.

[brv70086-bib-0273] Lesne, A. (2008). Robustness: confronting lessons from physics and biology. Biological Reviews 83, 509–532. 18823391 10.1111/j.1469-185X.2008.00052.x

[brv70086-bib-0274] Levina, A. , Herrmann, J. M. & Geisel, T. (2007). Dynamical synapses causing self‐organized criticality in neural networks. Nature Physics 3, 857–860.

[brv70086-bib-0275] Levina, A. , Herrmann, J. M. & Geisel, T. (2009). Phase transitions towards criticality in a neural system with adaptive interactions. Physical Review Letters 102, 118110. 19392248 10.1103/PhysRevLett.102.118110

[brv70086-bib-0276] Levit‐Binnun, N. & Golland, Y. (2012). Finding behavioral and network indicators of brain vulnerability. Frontiers in Human Neuroscience 6, 10. 22347174 10.3389/fnhum.2012.00010 PMC3273890

[brv70086-bib-0277] Liang, J. , Yang, Z. & Zhou, C. (2024). Excitation–inhibition balance, neural criticality, and activities in neuronal circuits. The Neuroscientist 31, 10738584231221766. 10.1177/10738584231221766 38291889

[brv70086-bib-0278] Liddle, P. F. & Morris, D. L. (1991). Schizophrenic syndromes and frontal lobe performance. British Journal of Psychiatry 158, 340–345. 10.1192/bjp.158.3.340 2036532

[brv70086-bib-0279] Lienkaemper, C. , Shiu, A. & Woodstock, Z. (2017). Obstructions to convexity in neural codes. Advances in Applied Mathematics 85, 31–59.

[brv70086-bib-0280] Liu, R. , Chen, P. , Aihara, K. & Chen, L. (2015). Identifying early‐warning signals of critical transitions with strong noise by dynamical network markers. Scientific Reports 5, 17501. 26647650 10.1038/srep17501 PMC4673532

[brv70086-bib-0281] Liu, X. , Li, D. , Ma, M. , Szymanski, B. K. , Stanley, H. E. & Gao, J. (2022). Network resilience. Physics Reports 971, 1–108.

[brv70086-bib-0282] Liu, Y. , Dehmamy, N. & Barabási, A. L. (2021). Isotopy and energy of physical networks. Nature Physics 17, 216–222.

[brv70086-bib-0283] Liu, Y. Y. & Barabási, A. L. (2016). Control principles of complex systems. Reviews of Modern Physics 88, 035006.

[brv70086-bib-0284] Lo, C.‐Y. , Wang, P.‐N. , Chou, K.‐H. , Wang, J. , He, Y. & Lin, C.‐P. (2010). Diffusion tensor tractography reveals abnormal topological organization in structural cortical networks in Alzheimer's disease. Journal of Neuroscience 30, 16876–16885. 21159959 10.1523/JNEUROSCI.4136-10.2010 PMC6634928

[brv70086-bib-0285] Luders, H. O. , Engel, J. & Munari, C. (1993). General principles. In Surgical Treatment of the Epilepsies, Second Edition (ed. J. J. Engel ), pp. 137–153. Raven Press, New York.

[brv70086-bib-0286] Ma, W. , Trusina, A. , El‐Samad, H. , Lim, W. A. & Tang, C. (2009). Defining network topologies that can achieve biochemical adaptation. Cell 138, 760–773. 19703401 10.1016/j.cell.2009.06.013 PMC3068210

[brv70086-bib-0287] Ma, Z. , Turrigiano, G. G. , Wessel, R. & Hengen, K. B. (2019). Cortical circuit dynamics are homeostatically tuned to criticality *in vivo* . Neuron 104, 655–664.e4. 31601510 10.1016/j.neuron.2019.08.031 PMC6934140

[brv70086-bib-0288] Machta, B. B. , Chachra, R. , Transtrum, M. K. & Sethna, J. P. (2013). Parameter space compression underlies emergent theories and predictive models. Science 342, 604–607. 24179222 10.1126/science.1238723

[brv70086-bib-0289] Mackey, M. C. & Glass, L. (1977). Oscillation and chaos in physiological control systems. Science 197, 287–289. 267326 10.1126/science.267326

[brv70086-bib-0290] Majhi, S. , Rakshit, B. , Sharma, A. , Kurths, J. & Ghosh, D. (2024). Dynamical robustness of network of oscillators. Physics Reports 1082, 1–46.

[brv70086-bib-0291] Makin, T. R. & Krakauer, J. W. (2023). Against cortical reorganisation. eLife 12, e84716. 37986628 10.7554/eLife.84716 PMC10662956

[brv70086-bib-0292] Mandal, A. S. , Wiener, C. , Assem, M. , Romero‐Garcia, R. , Coelho, P. , McDonald, A. , Woodberry, E. , Morris, R. C. , Price, S. J. , Duncan, J. & Santarius, T. (2024). Tumour‐infiltrated cortex participates in large‐scale cognitive circuits. Cortex 173, 1–15. 38354669 10.1016/j.cortex.2024.01.004 PMC10988771

[brv70086-bib-0293] Manrubia, S. , Cuesta, J. A. , Aguirre, J. , Ahnert, S. E. , Altenberg, L. , Cano, A. V. , Catalán, P. , Diaz‐Uriarte, R. , Elena, S. F. , García‐Martín, J. A. & Hogeweg, P. (2021). From genotypes to organisms: state‐of‐the‐art and perspectives of a cornerstone in evolutionary dynamics. Physics of Life Reviews 38, 55–106. 34088608 10.1016/j.plrev.2021.03.004

[brv70086-bib-0294] Marder, E. & Goaillard, J. M. (2006). Variability, compensation and homeostasis in neuron and network function. Nature Reviews Neuroscience 7, 563–574. 16791145 10.1038/nrn1949

[brv70086-bib-0295] Markram, H. , Lübke, J. , Frotscher, M. & Sakmann, B. (1997). Regulation of synaptic efficacy by coincidence of postsynaptic APs and EPSPs. Science 275, 213–215. 8985014 10.1126/science.275.5297.213

[brv70086-bib-0296] Markram, H. & Tsodyks, M. (1996). Redistribution of synaptic efficacy between neocortical pyramidal neurons. Nature 382, 807–810. 8752273 10.1038/382807a0

[brv70086-bib-0297] Markram, K. & Markram, H. (2010). The intense world theory – a unifying theory of the neurobiology of autism. Frontiers in Human Neuroscience 4, 224. 21191475 10.3389/fnhum.2010.00224 PMC3010743

[brv70086-bib-0298] Martinello, M. , Hidalgo, J. , Maritan, A. , Di Santo, S. , Plenz, D. & Muñoz, M. A. (2017). Neutral theory and scale‐free neural dynamics. Physical Review X 7, 041071.

[brv70086-bib-0299] Martínez, J. H. , Buldú, J. M. , Papo, D. , Fallani, F. D. V. & Chavez, M. (2018a). Role of inter‐hemispheric connections in functional brain networks. Scientific Reports 8, 10246. 29980771 10.1038/s41598-018-28467-x PMC6035280

[brv70086-bib-0300] Martínez, J. H. , López, M. E. , Ariza, P. , Chavez, M. , Pineda‐Pardo, J. A. , López‐Sanz, D. , Gil, P. , Maestú, F. & Buldú, J. M. (2018b). Functional brain networks reveal the existence of cognitive reserve and the interplay between network topology and dynamics. Scientific Reports 8, 10525. 30002460 10.1038/s41598-018-28747-6 PMC6043549

[brv70086-bib-0301] Maslennikov, O. V. & Nekorkin, V. I. (2017). Adaptive dynamical networks. Physics‐Uspekhi 60, 694–704.

[brv70086-bib-0302] Massimini, M. , Corbetta, M. , Sanchez‐Vives, M. V. , Andrillon, T. , Deco, G. , Rosanova, M. & Sarasso, S. (2024). Sleep‐like cortical dynamics during wakefulness and their network effects following brain injury. Nature Communications 15, 7207. 10.1038/s41467-024-51586-1 PMC11341729 39174560

[brv70086-bib-0303] Mastrogiuseppe, F. & Ostojic, S. (2018). Linking connectivity, dynamics, and computations in low‐rank recurrent neural networks. Neuron 99, 609–623. 30057201 10.1016/j.neuron.2018.07.003

[brv70086-bib-0304] Matsumoto, Y. (2002). An Introduction to Morse Theory, Edition (Volume 208). American Mathematical Soc.

[brv70086-bib-0305] Mattar, M. G. , Cole, M. W. , Thompson‐Schill, S. L. & Bassett, D. S. (2015). A functional cartography of cognitive systems. PLoS Computational Biology 11, e1004533. 26629847 10.1371/journal.pcbi.1004533 PMC4668064

[brv70086-bib-0306] Matthäus, F. (2006). Diffusion versus network models as descriptions for the spread of prion diseases in the brain. Journal of Theoretical Biology 240, 104–113. 16219329 10.1016/j.jtbi.2005.08.030

[brv70086-bib-0307] Mattson, M. P. & Magnus, T. (2006). Ageing and neuronal vulnerability. Nature Reviews Neuroscience 7, 278–294. 16552414 10.1038/nrn1886 PMC3710114

[brv70086-bib-0308] McGhie, A. & Chapman, J. (1961). Disorders of attention and perception in early schizophrenia. British Journal of Medical Psychology 34, 103–116. 13773940 10.1111/j.2044-8341.1961.tb00936.x

[brv70086-bib-0309] McIntosh, A. R. & Jirsa, V. K. (2019). The hidden repertoire of brain dynamics and dysfunction. Network Neuroscience 3, 994–1008. 31637335 10.1162/netn_a_00107 PMC6777946

[brv70086-bib-0310] Medaglia, J. D. , Pasqualetti, F. , Hamilton, R. H. , Thompson‐Schill, S. L. & Bassett, D. S. (2017). Brain and cognitive reserve: translation via network control theory. Neuroscience & Biobehavioral Reviews 75, 53–64. 28104411 10.1016/j.neubiorev.2017.01.016 PMC5359115

[brv70086-bib-0311] Mehta, A. , Prabhakar, M. , Kumar, P. , Deshmukh, R. & Sharma, P. L. (2013). Excitotoxicity: bridge to various triggers in neurodegenerative disorders. European Journal of Pharmacology 698, 6–18. 23123057 10.1016/j.ejphar.2012.10.032

[brv70086-bib-0312] Meisel, C. & Gross, T. (2009). Adaptive self‐organization in a realistic neural network model. Physical Review E 80, 061917. 10.1103/PhysRevE.80.061917 20365200

[brv70086-bib-0313] Menck, P. J. , Heitzig, J. , Marwan, N. & Kurths, J. (2013). How basin stability complements the linear‐stability paradigm. Nature Physics 9, 89–92.

[brv70086-bib-0314] Menesse, E. , Marin, B. & Kinouchi, O. (2022). Homeostatic criticality in neuronal networks. Chaos, Solitons & Fractals 156, 111877.

[brv70086-bib-0315] Mermin, N. D. (1979). The topological theory of defects in ordered media. Reviews of Modern Physics 51, 591–648.

[brv70086-bib-0316] Meunier, D. , Lambiotte, R. & Bullmore, E. T. (2010). Modular and hierarchically modular organization of brain networks. Frontiers in Neuroscience 4, 200. 21151783 10.3389/fnins.2010.00200 PMC3000003

[brv70086-bib-0317] Millán, A. P. , Torres, J. J. & Bianconi, G. (2018). Complex network geometry and frustrated synchronization. Scientific Reports 8, 9910. 29967410 10.1038/s41598-018-28236-w PMC6028575

[brv70086-bib-0318] Millán, A. P. , van Straaten, E. C. , Stam, C. J. , Nissen, I. A. , Idema, S. , Baayen, J. C. , Van Mieghem, P. & Hillebrand, A. (2022). Epidemic models characterize seizure propagation and the effects of epilepsy surgery in individualized brain networks based on MEG and invasive EEG recordings. Scientific Reports 12, 4086. 35260657 10.1038/s41598-022-07730-2 PMC8904850

[brv70086-bib-0319] Miller, K. L. , Alfaro‐Almagro, F. , Bangerter, N. K. , Thomas, D. L. , Yacoub, E. , Xu, J. , Bartsch, A. J. , Jbabdi, S. , Sotiropoulos, S. N. , Andersson, J. L. & Griffanti, L. (2016). Multimodal population brain imaging in the UK biobank prospective epidemiological study. Nature Neuroscience 19, 1523–1536. 27643430 10.1038/nn.4393 PMC5086094

[brv70086-bib-0320] Milo, R. , Shen‐Orr, S. , Itzkovitz, S. , Kashtan, N. , Chklovskii, D. & Alon, U. (2002). Network motifs: simple building blocks of complex networks. Science 298, 824–827. 12399590 10.1126/science.298.5594.824

[brv70086-bib-0321] Mišić, B. , Betzel, R. F. , Nematzadeh, A. , Goñi, J. , Griffa, A. , Hagmann, P. , Flammini, A. , Ahn, Y. Y. & Sporns, O. (2015). Cooperative and competitive spreading dynamics on the human connectome. Neuron 86, 1518–1529. 26087168 10.1016/j.neuron.2015.05.035

[brv70086-bib-0322] Mobasheri, A. & Loeser, R. (2024). Clinical phenotypes, molecular endotypes and theratypes in OA therapeutic development. Nature Reviews Rheumatology 20, 525–526. 10.1038/s41584-024-01126-4 38760581

[brv70086-bib-0323] Mochizuki, A. , Fiedler, B. , Kurosawa, G. & Saito, D. (2013). Dynamics and control at feedback vertex sets. II: a faithful monitor to determine the diversity of molecular activities in regulatory networks. Journal of Theoretical Biology 335, 130–146. 23774067 10.1016/j.jtbi.2013.06.009

[brv70086-bib-0324] Mohseni‐Kabir, A. , Pant, M. , Towsley, D. , Guha, S. & Swami, A. (2021). Percolation thresholds for robust network connectivity. Journal of Statistical Mechanics: Theory and Experiment 2021, 013212.

[brv70086-bib-0325] Moretti, P. , Dietemann, B. , Esfandiary, N. & Zaiser, M. (2018). Avalanche precursors of failure in hierarchical fuse networks. Scientific Reports 8, 12090. 30108308 10.1038/s41598-018-30539-x PMC6092438

[brv70086-bib-0326] Moretti, P. & Muñoz, M. A. (2013). Griffiths phases and the stretching of criticality in brain networks. Nature Communications 4, 2521. 10.1038/ncomms3521 24088740

[brv70086-bib-0327] Moretti, P. , Renner, J. , Safari, A. & Zaiser, M. (2019). Graph theoretical approaches for the characterization of damage in hierarchical materials. The European Physical Journal B 92, 97.

[brv70086-bib-0328] Moretti, P. & Zaiser, M. (2019). Network analysis predicts failure of materials and structures. Proceedings of the National Academy of Sciences of the United States of America 116, 16666–16668. 31375627 10.1073/pnas.1911715116 PMC6708317

[brv70086-bib-0329] Morrell, F. , Whisler, W. W. & Bleck, T. P. (1989). Multiple subpial transection: a new approach to the surgical treatment of focal epilepsy. Journal of Neurosurgery 70, 231–239. 2492335 10.3171/jns.1989.70.2.0231

[brv70086-bib-0330] Morrison, K. & Curto, C. (2019). Predicting neural network dynamics via graphical analysis. In Algebraic and Combinatorial Computational Biology, pp. 241–277. Academic Press.

[brv70086-bib-0331] Mortimer, J. A. (1988). Do psychosocial risk factors contribute to Alzheimer's disease. In Etiology of Dementia of Alzheimer's Type (eds A. S. Henderson and J. H. Henderson ), pp. 39–52. Wiley, New York.

[brv70086-bib-0332] Mueller, S. G. , Laxer, K. D. , Barakos, J. , Cheong, I. , Garcia, P. & Weiner, M. W. (2009). Widespread neocortical abnormalities in temporal lobe epilepsy with and without mesial sclerosis. NeuroImage 46, 353–359. 19249372 10.1016/j.neuroimage.2009.02.020 PMC2799165

[brv70086-bib-0333] Murphy, T. H. & Corbett, D. (2009). Plasticity during stroke recovery: from synapse to behaviour. Nature Reviews Neuroscience 10, 861–872. 19888284 10.1038/nrn2735

[brv70086-bib-0334] Murugan, A. & Vaikuntanathan, S. (2017). Topologically protected modes in non‐equilibrium stochastic systems. Nature Communications 8, 13881. 10.1038/ncomms13881 PMC5234070 28071644

[brv70086-bib-0335] Nelson, S. B. & Valakh, V. (2015). Excitatory/inhibitory balance and circuit homeostasis in autism spectrum disorders. Neuron 87, 684–698. 26291155 10.1016/j.neuron.2015.07.033 PMC4567857

[brv70086-bib-0336] Nicolle, C. (1933). Leçons du Collège de France: destin des maladies infectieuses. Felix Alcan, Paris.

[brv70086-bib-0337] Nijhout, H. F. , Best, J. A. & Reed, M. C. (2019). Systems biology of robustness and homeostatic mechanisms. Wiley Interdisciplinary Reviews: Systems Biology and Medicine 11, e1440. 30371009 10.1002/wsbm.1440

[brv70086-bib-0338] Nishimori, H. & Ortiz, G. (2010). Elements of Phase Transitions and Critical Phenomena. Oxford University Press, Oxford.

[brv70086-bib-0339] Nowotny, T. & Rabinovich, M. I. (2007). Dynamical origin of independent spiking and bursting activity in neural microcircuits. Physical Review Letters 98, 128106. 17501162 10.1103/PhysRevLett.98.128106

[brv70086-bib-0340] Oberman, L. & Pascual‐Leone, A. (2013). Changes in plasticity across the lifespan: cause of disease and target for intervention. Prog. Brain Research 207, 91–120. 24309252 10.1016/B978-0-444-63327-9.00016-3 PMC4392917

[brv70086-bib-0341] Oberman, L. M. , Rotenberg, A. & Pascual‐Leone, A. (2014). Aberrant brain plasticity in autism spectrum disorders. In Cognitive Plasticity in Neurologic Disorders (eds J. I. Tracy , B. M. Hampstead and K. Sathian ), pp. 176–196.

[brv70086-bib-0342] Ocker, G. K. , Josić, K. , Shea‐Brown, E. & Buice, M. A. (2017). Linking structure and activity in nonlinear spiking networks. PLoS Computational Biology 13, e1005583. 28644840 10.1371/journal.pcbi.1005583 PMC5507396

[brv70086-bib-0343] Ocker, G. K. , Litwin‐Kumar, A. & Doiron, B. (2015). Self‐organization of microcircuits in networks of spiking neurons with plastic synapses. PLoS Computational Biology 11, e1004458. 26291697 10.1371/journal.pcbi.1004458 PMC4546203

[brv70086-bib-0344] Ódor, G. (2008). Universality in Nonequilibrium Lattice Systems: Theoretical Foundations. World Scientific.

[brv70086-bib-0345] Okano, H. , Miyawaki, A. & Kasai, K. (2015). Brain/MINDS: brain‐mapping project in Japan. Philosophical Transactions of the Royal Society B 370, 20140310. 10.1098/rstb.2014.0310 PMC4387516 25823872

[brv70086-bib-0346] Olmi, S. , Petkoski, S. , Guye, M. , Bartolomei, F. & Jirsa, V. (2019). Controlling seizure propagation in large‐scale brain networks. PLoS Computational Biology 15, e1006805. 30802239 10.1371/journal.pcbi.1006805 PMC6405161

[brv70086-bib-0347] Ostojic, S. & Fusi, S. (2024). Computational role of structure in neural activity and connectivity. Trends in Cognitive Sciences 28, 677–690. 38553340 10.1016/j.tics.2024.03.003 PMC12177215

[brv70086-bib-0348] Ott, E. , Grebogi, C. & Yorke, J. A. (1990). Controlling chaos. Physical Review Letters 64, 1196–1199. 10041332 10.1103/PhysRevLett.64.1196

[brv70086-bib-0349] Palla, G. , Derényi, I. , Farkas, I. & Vicsek, T. (2004). Statistical mechanics of topological phase transitions in networks. Physical Review E 69, 046117. 10.1103/PhysRevE.69.046117 15169079

[brv70086-bib-0350] Panas, D. , Amin, H. , Maccione, A. , Muthmann, O. , van Rossum, M. , Berdondini, L. & Hennig, M. H. (2015). Sloppiness in spontaneously active neuronal networks. Journal of Neuroscience 35, 8480–8492. 26041916 10.1523/JNEUROSCI.4421-14.2015 PMC4452554

[brv70086-bib-0351] Pandya, S. , Kuceyeski, A. & Raj, A. (2017). The brain's structural connectome mediates the relationship between regional neuroimaging biomarkers in Alzheimer's disease. Journal of Alzheimer's Disease 55, 1639–1657. 10.3233/JAD-160090 27911289

[brv70086-bib-0352] Pandya, V. A. & Patani, R. (2021). Region‐specific vulnerability in neurodegeneration: lessons from normal ageing. Ageing Research Reviews 67, 101311. 33639280 10.1016/j.arr.2021.101311 PMC8024744

[brv70086-bib-0353] Papo, D. (2013a). Brain temperature: what it means and what it can do for (cognitive) neuroscientists. arXiv, 1310.2906. 10.48550/arXiv.1310.2906

[brv70086-bib-0354] Papo, D. (2013b). Time scales in cognitive neuroscience. Frontiers in Physiology 4, 86. 23626578 10.3389/fphys.2013.00086 PMC3630296

[brv70086-bib-0355] Papo, D. (2015). How can we study reasoning in the brain? Frontiers in Human Neuroscience 9, 222. 25964755 10.3389/fnhum.2015.00222 PMC4408754

[brv70086-bib-0356] Papo, D. (2019a). Gauging functional brain activity: from distinguishability to accessibility. Frontiers in Physiology 10, 509. 31139089 10.3389/fphys.2019.00509 PMC6517676

[brv70086-bib-0357] Papo, D. (2019b). Neurofeedback: principles, appraisal and outstanding issues. European Journal of Neuroscience 49, 1454–1469. 30570194 10.1111/ejn.14312

[brv70086-bib-0358] Papo, D. & Buldú, J. M. (2024). Does the brain behave like a (complex) network? I. Dynamics. Physics of Life Reviews 48, 47–98. 38145591 10.1016/j.plrev.2023.12.006

[brv70086-bib-0359] Papo, D. , Goñi, J. & Buldú, J. M. (2017). Editorial: on the relation of dynamics and structure in brain networks. Chaos 27, 047201. 28456177 10.1063/1.4981391

[brv70086-bib-0360] Papo, D. , Zanin, M. , Pineda, J. A. , Boccaletti, S. & Buldú, J. M. (2014). Brain networks: great expectations, hard times, and the big leap forward. Philosophical Transactions of the Royal Society B: Biological Sciences 369, 20130525. 10.1098/rstb.2013.0525 PMC4150300 25180303

[brv70086-bib-0361] Papo, I. , Quattrini, A. , Provinciali, L. , Rychlicki, F. , Del Pesce, M. , Paggi, A. , Ortenzi, F. , Recchioni, M. A. & Censori, B. (1990). Callosotomy for the treatment of drug resistant generalized seizures. In Neurosurgical Aspects of Epilepsy: Proceedings of the Fourth Advanced Seminar in Neurosurgical Research of the European Association of Neurosurgical Societies Bresseo di Teolo, Padova, May 17–18, 1989, pp. 134–135. Springer Vienna. 10.1007/978-3-7091-9104-0_27 2129084

[brv70086-bib-0362] Park, E. , Velumian, A. A. & Fehlings, M. G. (2004). The role of excitotoxicity in secondary mechanisms of spinal cord injury: a review with an emphasis on the implications for white matter degeneration. Journal of Neurotrauma 21, 754–774. 15253803 10.1089/0897715041269641

[brv70086-bib-0363] Parshani, R. , Buldyrev, S. V. & Havlin, S. (2010). Interdependent networks: reducing the coupling strength leads to a change from a first to second order percolation transition. Physical Review Letters 105, 048701. 20867893 10.1103/PhysRevLett.105.048701

[brv70086-bib-0364] Parshani, R. , Rozenblat, C. , Ietri, D. , Ducruet, C. & Havlin, S. (2011). Inter‐similarity between coupled networks. EPL (Europhysics Letters) 92, 68002.

[brv70086-bib-0365] Pascual‐Leone, A. , Freitas, C. , Oberman, L. , Horvath, J. C. , Halko, M. , Eldaief, M. , Bashir, S. , Vernet, M. , Shafi, M. , Westover, B. & Vahabzadeh‐Hagh, A. M. (2011). Characterizing brain cortical plasticity and network dynamics across the age‐span in health and disease with TMS‐EEG and TMS‐fMRI. Brain Topography 24, 302–315. 21842407 10.1007/s10548-011-0196-8 PMC3374641

[brv70086-bib-0366] Patania, A. , Selvaggi, P. , Veronese, M. , Dipasquale, O. , Expert, P. & Petri, G. (2019). Topological gene expression networks recapitulate brain anatomy and function. Network Neuroscience 3, 744–762. 31410377 10.1162/netn_a_00094 PMC6663211

[brv70086-bib-0367] Pathak, A. , Menon, S. N. & Sinha, S. (2024). A hierarchy index for networks in the brain reveals a complex entangled organizational structure. Proceedings of the National Academy of Sciences of the United States of America 121, e2314291121. 38923990 10.1073/pnas.2314291121 PMC11228506

[brv70086-bib-0368] Payne, J. L. & Wagner, A. (2019). The causes of evolvability and their evolution. Nature Reviews Genetics 20, 24–38. 10.1038/s41576-018-0069-z 30385867

[brv70086-bib-0369] Pazó, D. & Montbrió, E. (2006). Universal behavior in populations composed of excitable and self‐oscillatory elements. Physical Review E 73, 55202. 10.1103/PhysRevE.73.055202 16802985

[brv70086-bib-0370] Pecora, L. M. , Sorrentino, F. , Hagerstrom, A. M. , Murphy, T. E. & Roy, R. (2014). Cluster synchronization and isolated desynchronization in complex networks with symmetries. Nature Communications 5, 4079. 10.1038/ncomms5079 24923317

[brv70086-bib-0371] Penner, I. K. & Aktas, O. (2017). Functional reorganization is a maladaptive response to injury ‐ NO. Multiple Sclerosis Journal 23, 193–194. 27932693 10.1177/1352458516679895

[brv70086-bib-0372] Peraza, L. R. , Díaz‐Parra, A. , Kennion, O. , Moratal, D. , Taylor, J. P. , Kaiser, M. , Bauer, R. & Alzheimer's Disease Neuroimaging Initiative (2019). Structural connectivity centrality changes mark the path toward Alzheimer's disease. Alzheimer's & Dementia: Diagnosis, Assessment & Disease Monitoring 11, 98–107. 10.1016/j.dadm.2018.12.004 PMC6350419 30723773

[brv70086-bib-0373] Pereira, J. B. , Strandberg, T. O. , Palmqvist, S. , Volpe, G. , van Westen, D. , Westman, E. , Hansson, O. & Alzheimer's Disease Neuroimaging Initiative (2018). Amyloid network topology characterizes the progression of Alzheimer's disease during the predementia stages. Cerebral Cortex 28, 340–349. 29136123 10.1093/cercor/bhx294 PMC6454565

[brv70086-bib-0374] Pernice, V. , Deger, M. , Cardanobile, S. & Rotter, S. (2013). The relevance of network micro‐structure for neural dynamics. Frontiers in Computational Neuroscience 7, 72. 23761758 10.3389/fncom.2013.00072 PMC3671286

[brv70086-bib-0375] Perra, N. , Baronchelli, A. , Mocanu, D. , Gonçalves, B. , Pastor‐Satorras, R. & Vespignani, A. (2012). Random walks and search in time‐varying networks. Physical Review Letters 109, 238701. 23368274 10.1103/PhysRevLett.109.238701

[brv70086-bib-0376] Peters, J. F. (2016). Computational proximity. In Intelligent Systems Reference Library (Volume 102). Springer, Cham.

[brv70086-bib-0377] Peters, J. F. (2018). Proximal fiber bundles on nerve complexes. In Mathematical Analysis and Applications: Selected Topics (eds M. Ruzhansky , H. Dutta and R. P. Agarwal ), pp. 517–536.

[brv70086-bib-0378] Petri, G. , Musslick, S. , Dey, B. , Özcimder, K. , Turner, D. , Ahmed, N. K. , Willke, T. L. & Cohen, J. D. (2021). Topological limits to the parallel processing capability of network architectures. Nature Physics 17, 646–651.

[brv70086-bib-0379] Pettini, M. (2007). Geometry and Topology in Hamiltonian Dynamics and Statistical Mechanics. Springer Science & Business Media.

[brv70086-bib-0380] Plis, S. M. , Sui, J. , Lane, T. , Roy, S. , Clark, V. P. , Potluru, V. K. , Huster, R. J. , Michael, A. , Sponheim, S. R. , Weisend, M. P. & Calhoun, V. D. (2014). High‐order interactions observed in multi‐task intrinsic networks are dominant indicators of aberrant brain function in schizophrenia. NeuroImage 102, 35–48. 23876245 10.1016/j.neuroimage.2013.07.041 PMC3896503

[brv70086-bib-0381] Poo, M. M. , Du, J. L. , Ip, N. Y. , Xiong, Z. Q. , Xu, B. & Tan, T. (2016). China brain project: basic neuroscience, brain diseases, and brain‐inspired computing. Neuron 92, 591–596. 27809999 10.1016/j.neuron.2016.10.050

[brv70086-bib-0382] Porter, M. A. & Gleeson, J. P. (2016). Dynamical systems on networks. Frontiers in Applied Mathematics and Statistics 4, 1–80.

[brv70086-bib-0383] Pósfai, M. , Szegedy, B. , Bačić, I. , Blagojević, L. , Abért, M. , Kertész, J. , Lovász, L. & Barabási, A. L. (2024). Impact of physicality on network structure. Nature Physics 20, 142–149.

[brv70086-bib-0384] Powell, F. , Tosun, D. , Sadeghi, R. , Weiner, M. , Raj, A. & Alzheimer's Disease Neuroimaging Initiative (2018). Preserved structural network organization mediates pathology spread in Alzheimer's disease spectrum despite loss of white matter tract integrity. Journal of Alzheimer's Disease 65, 747–764. 10.3233/JAD-170798 PMC6152926 29578480

[brv70086-bib-0385] Pozo, K. & Goda, Y. (2010). Unraveling mechanisms of homeostatic synaptic plasticity. Neuron 66, 337–351. 20471348 10.1016/j.neuron.2010.04.028 PMC3021747

[brv70086-bib-0386] Price, C. J. & Friston, K. J. (2002). Degeneracy and cognitive anatomy. Trends in Cognitive Sciences 6, 416–421. 12413574 10.1016/s1364-6613(02)01976-9

[brv70086-bib-0387] Rabinovich, M. , Huerta, R. & Laurent, G. (2008). Transient dynamics for neural processing. Science 321, 48–50. 18599763 10.1126/science.1155564

[brv70086-bib-0388] Radicchi, F. & Arenas, A. (2013). Abrupt transition in the structural formation of interconnected networks. Nature Physics 9, 717–720.

[brv70086-bib-0389] Raj, A. , Kuceyeski, A. & Weiner, M. (2012). A network diffusion model of disease progression in dementia. Neuron 73, 1204–1215. 22445347 10.1016/j.neuron.2011.12.040 PMC3623298

[brv70086-bib-0390] Raj, A. & Powell, F. (2018). Models of network spread and network degeneration in brain disorders. Biological Psychiatry: Cognitive Neuroscience and Neuroimaging 3, 788–797. 30170711 10.1016/j.bpsc.2018.07.012 PMC6219468

[brv70086-bib-0391] Ramesh, P. , Confavreux, B. , Gonçalves, P. J. , Vogels, T. P. & Macke, J. H. (2023). Indistinguishable network dynamics can emerge from unalike plasticity rules. bioRxiv 2023‐11.

[brv70086-bib-0392] Rapisardi, G. , Kryven, I. & Arenas, A. (2022). Percolation in networks with local homeostatic plasticity. Nature Communications 13, 122. 10.1038/s41467-021-27736-0 PMC8748765 35013243

[brv70086-bib-0393] Ratté, S. & Prescott, S. A. (2016). Afferent hyperexcitability in neuropathic pain and the inconvenient truth about its degeneracy. Current Opinion in Neurobiology 36, 31–37. 26363576 10.1016/j.conb.2015.08.007

[brv70086-bib-0394] Recanatesi, S. , Pereira‐Obilinovic, U. , Murakami, M. , Mainen, Z. & Mazzucato, L. (2022). Metastable attractors explain the variable timing of stable behavioral action sequences. Neuron 110, 139–153. 34717794 10.1016/j.neuron.2021.10.011 PMC9194601

[brv70086-bib-0395] Reis, S. D. , Hu, Y. , Babino, A. , Andrade, J. S. Jr. , Canals, S. , Sigman, M. & Makse, H. A. (2014). Avoiding catastrophic failure in correlated networks of networks. Nature Physics 10, 762–767.

[brv70086-bib-0396] Ren, X. L. , Gleinig, N. , Helbing, D. & Antulov‐Fantulin, N. (2019). Generalized network dismantling. Proceedings of the National Academy of Sciences of the United States of America 116, 6554–6559. 30877241 10.1073/pnas.1806108116 PMC6452684

[brv70086-bib-0397] Riederer, F. , Lanzenberger, R. , Kaya, M. , Prayer, D. , Serles, W. & Baumgartner, C. (2008). Network atrophy in temporal lobe epilepsy: a voxel‐based morphometry study. Neurology 71, 419–425. 18678824 10.1212/01.wnl.0000324264.96100.e0

[brv70086-bib-0398] Rikkert, M. G. O. , Dakos, V. , Buchman, T. G. , De Boer, R. , Glass, L. , Cramer, A. O. , Levin, S. , Van Nes, E. , Sugihara, G. , Ferrari, M. D. & Tolner, E. A. (2016). Slowing down of recovery as generic risk marker for acute severity transitions in chronic diseases. Critical Care Medicine 44, 601–606. 26765499 10.1097/CCM.0000000000001564

[brv70086-bib-0399] Rings, T. , Mazarei, M. , Akhshi, A. , Geier, C. , Tabar, M. R. R. & Lehnertz, K. (2019a). Traceability and dynamical resistance of precursor of extreme events. Scientific Reports 9, 1744. 30741977 10.1038/s41598-018-38372-y PMC6370838

[brv70086-bib-0400] Rings, T. , von Wrede, R. & Lehnertz, K. (2019 *b*). Precursors of seizures due to specific spatial‐temporal modifications of evolving large‐scale epileptic brain networks. Scientific Reports 9, 10623. 31337840 10.1038/s41598-019-47092-w PMC6650408

[brv70086-bib-0401] Roberts, J. A. , Gollo, L. L. , Abeysuriya, R. G. , Roberts, G. , Mitchell, P. B. , Woolrich, M. W. & Breakspear, M. (2019). Metastable brain waves. Nature Communications 10, 1056. 10.1038/s41467-019-08999-0 PMC6401142 30837462

[brv70086-bib-0402] Roberts, J. A. , Perry, A. , Lord, A. R. , Roberts, G. , Mitchell, P. B. , Smith, R. E. , Calamante, F. & Breakspear, M. (2016). The contribution of geometry to the human connectome. NeuroImage 124, 379–393. 26364864 10.1016/j.neuroimage.2015.09.009

[brv70086-bib-0403] Rocca, M. A. & Filippi, M. (2017). Functional reorganization is a maladaptive response to injury ‐ YES. Multiple Sclerosis Journal 23, 191–193. 27932694 10.1177/1352458516667242

[brv70086-bib-0404] Rocca, M. A. , Valsasina, P. , Meani, A. , Falini, A. , Comi, G. & Filippi, M. (2016). Impaired functional integration in multiple sclerosis: a graph theory study. Brain Structure and Function 221, 115–131. 25257603 10.1007/s00429-014-0896-4

[brv70086-bib-0405] Rodgers, N. , Tiňo, P. & Johnson, S. (2023). Strong connectivity in real directed networks. Proceedings of the National Academy of Sciences of the United States of America 120, e2215752120. 36927153 10.1073/pnas.2215752120 PMC10041124

[brv70086-bib-0406] Rossi, L. , Torsello, A. & Hancock, E. R. (2015). Measuring graph similarity through continuous‐time quantum walks and the quantum Jensen‐Shannon divergence. Physical Review E 91, 022815. 10.1103/PhysRevE.91.022815 25768560

[brv70086-bib-0407] Rossi, L. F. , Wykes, R. C. , Kullmann, D. M. & Carandini, M. (2017). Focal cortical seizures start as standing waves and propagate respecting homotopic connectivity. Nature Communications 8, 217. 10.1038/s41467-017-00159-6 PMC5550430 28794407

[brv70086-bib-0408] Roth, D. M. & Balch, W. E. (2011). Modeling general proteostasis: proteome balance in health and disease. Current Opinion in Chemical Biology 23, 126–134. 10.1016/j.ceb.2010.11.001 PMC3077458 21131189

[brv70086-bib-0409] Rozenfeld, H. D. , Song, C. & Makse, H. A. (2010). Small‐world to fractal transition in complex networks: a renormalization group approach. Physical Review Letters 104, 025701. 20366610 10.1103/PhysRevLett.104.025701

[brv70086-bib-0410] Rubinov, M. & Bullmore, E. (2013). Fledgling pathoconnectomics of psychiatric disorders. Trends in Cognitive Sciences 17, 641–647. 24238779 10.1016/j.tics.2013.10.007

[brv70086-bib-0411] Rubinov, M. , Sporns, O. , Thivierge, J. P. & Breakspear, M. (2011). Neurobiologically realistic determinants of self‐organized criticality in networks of spiking neurons. PLoS Computational Biology 7, e1002038. 21673863 10.1371/journal.pcbi.1002038 PMC3107249

[brv70086-bib-0412] Rubenstein, J. L. R. & Merzenich, M. M. (2003). Model of autism: increased ratio of excitation/inhibition in key neural systems. Genes, Brain and Behavior 2, 255–267. 14606691 10.1034/j.1601-183x.2003.00037.x PMC6748642

[brv70086-bib-0413] Rule, M. E. & O'Leary, T. (2022). Self‐healing codes: how stable neural populations can track continually reconfiguring neural representations. Proceedings of the National Academy of Sciences of the United States of America 119, e2106692119. 35145024 10.1073/pnas.2106692119 PMC8851551

[brv70086-bib-0414] Rule, M. E. , O'Leary, T. & Harvey, C. D. (2019). Causes and consequences of representational drift. Current Opinion in Neurobiology 58, 141–147. 31569062 10.1016/j.conb.2019.08.005 PMC7385530

[brv70086-bib-0415] Rutkowski, D. T. & Hegde, R. S. (2010). Regulation of basal cellular physiology by the homeostatic unfolded protein response. Journal of Cell Biology 189, 783–794. 20513765 10.1083/jcb.201003138 PMC2878945

[brv70086-bib-0416] Sandhu, R. , Georgiou, T. , Reznik, E. , Zhu, L. , Kolesov, I. , Senbabaoglu, Y. & Tannenbaum, A. (2015). Graph curvature for differentiating cancer networks. Scientific Reports 5, 12323. 26169480 10.1038/srep12323 PMC4500997

[brv70086-bib-0417] Sanhedrai, H. , Gao, J. , Bashan, A. , Schwartz, M. , Havlin, S. & Barzel, B. (2022). Reviving a failed network through microscopic interventions. Nature Physics 18, 338–349.

[brv70086-bib-0418] Sanhedrai, H. & Havlin, S. (2023). Sustaining a network by controlling a fraction of nodes. Communications Physics 6, 22.

[brv70086-bib-0419] Santos, E. , Schöll, M. , Sánchez‐Porras, R. , Dahlem, M. A. , Silos, H. , Unterberg, A. , Dickhaus, H. & Sakowitz, O. W. (2014). Radial, spiral and reverberating waves of spreading depolarization occur in the gyrencephalic brain. NeuroImage 99, 244–255. 24852458 10.1016/j.neuroimage.2014.05.021

[brv70086-bib-0420] Santos, F. A. N. , Raposo, E. P. , Coutinho‐Filho, M. D. , Copelli, M. , Stam, C. J. & Douw, L. (2019). Topological phase transitions in functional brain networks. Physical Review E 100, 032414. 31640025 10.1103/PhysRevE.100.032414

[brv70086-bib-0421] Sanz Perl, Y. , Fittipaldi, S. , Campo, C. G. , Moguilner, S. , Cruzat, J. , Fraile‐Vazquez, M. E. , Herzog, R. , Kringelbach, M. L. , Deco, G. , Prado, P. & Ibanez, A. (2023). Model‐based whole‐brain perturbational landscape of neurodegenerative diseases. eLife 12, e83970. 36995213 10.7554/eLife.83970 PMC10063230

[brv70086-bib-0422] Saxena, S. & Caroni, P. (2011). Selective neuronal vulnerability in neurodegenerative diseases: from stressor thresholds to degeneration. Neuron 71, 35–48. 21745636 10.1016/j.neuron.2011.06.031

[brv70086-bib-0423] Scharfman, H. E. , Sollas, A. L. , Berger, R. E. & Goodman, J. H. (2003). Electrophysiological evidence of monosynaptic excitatory transmission between granule cells after seizure‐induced mossy fiber sprouting. Journal of Neurophysiology 90, 2536–2547. 14534276 10.1152/jn.00251.2003

[brv70086-bib-0424] Scheffer, M. , Bockting, C. L. , Borsboom, D. , Cools, R. , Delecroix, C. , Hartmann, J. A. , Kendler, K. S. , van de Leemput, I. , van der Maas, H. L. , van Nes, E. & Mattson, M. (2024). A dynamical systems view of psychiatric disorder ‐ practical implications: a review. JAMA Psychiatry 81, 624–630. 38568618 10.1001/jamapsychiatry.2024.0228

[brv70086-bib-0425] Schindler, K. , Bialonski, S. , Horstmann, M.‐T. , Elger, C. E. & Lehnertz, K. (2008). Evolving functional network properties and synchronizability during human epileptic seizures. Chaos 18, 033119. 19045457 10.1063/1.2966112

[brv70086-bib-0426] Schmidt, A. , Smieskova, R. , Aston, J. , Simon, A. , Allen, P. , Fusar‐Poli, P. , McGuire, P. K. , Riecher‐Rössler, A. , Stephan, K. E. & Borgwardt, S. (2013). Brain connectivity abnormalities predating the onset of psychosis: correlation with the effect of medication. JAMA Psychiatry 70, 903–912. 23824230 10.1001/jamapsychiatry.2013.117

[brv70086-bib-0427] Schneider, C. M. , Yazdani, N. , Araújo, N. A. , Havlin, S. & Herrmann, H. J. (2013). Towards designing robust coupled networks. Scientific Reports 3, 1969. 23752705 10.1038/srep01969 PMC3678138

[brv70086-bib-0428] Schnitzler, A. & Gross, J. (2005). Normal and pathological oscillatory communication in the brain. Nature Reviews Neuroscience 6, 285–296. 15803160 10.1038/nrn1650

[brv70086-bib-0429] Schuster, P. & Fontana, W. (1999). Chance and necessity in evolution: lessons from RNA. Physica D 133, 427–452.

[brv70086-bib-0430] Schwarze, A. C. , Jiang, J. , Wray, J. & Porter, M. A. (2024). Structural robustness and vulnerability of networks. arXiv:2409.07498.

[brv70086-bib-0431] Sedeño, L. , Piguet, O. , Abrevaya, S. , Desmaras, H. , García‐Cordero, I. , Baez, S. , Alethia de la Fuente, L. , Reyes, P. , Tu, S. , Moguilner, S. & Lori, N. (2017). Tackling variability: a multicenter study to provide a gold‐standard network approach for frontotemporal dementia. Human Brain Mapping 38, 3804–3822. 28474365 10.1002/hbm.23627 PMC6867023

[brv70086-bib-0432] Seeley, W. W. , Crawford, R. K. , Zhou, J. , Miller, B. L. & Greicius, M. D. (2009). Neurodegenerative diseases target large‐scale human brain networks. Neuron 62, 42–52. 19376066 10.1016/j.neuron.2009.03.024 PMC2691647

[brv70086-bib-0433] Shandilya, S. G. & Timme, M. (2011). Inferring network topology from complex dynamics. New Journal of Physics 13, 013004.

[brv70086-bib-0434] Shang, Y. (2019). Attack robustness and stability of generalized *k*‐cores. New Journal of Physics 21, 093013.

[brv70086-bib-0435] Shankar, S. , Souslov, A. , Bowick, M. J. , Marchetti, M. C. & Vitelli, V. (2022). Topological active matter. Nature Reviews Physics 4, 380–398.

[brv70086-bib-0436] Shew, W. , Clawson, W. , Pobst, J. , Karimipanah, Y. , Wright, N. C. & Wessel, R. (2015). Adaptation to sensory input tunes visual cortex to criticality. Nature Physics 11, 659–663.

[brv70086-bib-0437] Shi, Y. L. , Zeraati, R. , Levina, A. & Engel, T. A. (2023). Spatial and temporal correlations in neural networks with structured connectivity. Physical Review Research 5, 013005. 38938692 10.1103/physrevresearch.5.013005 PMC11210526

[brv70086-bib-0438] Shinbrot, T. , Ott, E. , Grebogi, C. & Yorke, J. A. (1990). Using chaos to direct trajectories to targets. Physical Review Letters 65, 3215–3218. 10042812 10.1103/PhysRevLett.65.3215

[brv70086-bib-0439] Shinomoto, S. & Kuramoto, Y. (1986). Phase transitions in active rotator systems. Progress of Theoretical Physics 75, 1105–1110.

[brv70086-bib-0440] Sidaros, A. , Engberg, A. W. , Sidaros, K. , Liptrot, M. G. , Herning, M. , Petersen, P. , Paulson, O. B. , Jernigan, T. L. & Rostrup, E. (2008). Diffusion tensor imaging during recovery from severe traumatic brain injury and relation to clinical outcome: a longitudinal study. Brain 131, 559–572. 18083753 10.1093/brain/awm294

[brv70086-bib-0441] Sigerist, H.‐E. (1932). *Introduction à la médecine*. (French translation) Paris, Payot.

[brv70086-bib-0442] Simhal, A. K. , Carpenter, K. L. , Nadeem, S. , Kurtzberg, J. , Song, A. , Tannenbaum, A. , Sapiro, G. & Dawson, G. (2020). Measuring robustness of brain networks in autism spectrum disorder with Ricci curvature. Scientific Reports 10, 10819. 32616759 10.1038/s41598-020-67474-9 PMC7331646

[brv70086-bib-0443] Simon, H. A. (1962). The architecture of complexity. Proceedings of the American Philosophical Society, Edition (Volume 106), pp. 467–482.

[brv70086-bib-0444] Song, C. , Wang, P. & Makse, H. (2008). A phase diagram for jammed matter. Nature 453, 629–632. 18509438 10.1038/nature06981

[brv70086-bib-0445] Sonnenschein, B. , Zaks, M. A. , Neiman, A. B. & Schimansky‐Geier, L. (2013). Excitable elements controlled by noise and network structure. European Physical Journal Special Topics 222, 2517–2529.

[brv70086-bib-0446] Spencer, S. S. (2002). Neural networks in human epilepsy: evidence of and implications for treatment. Epilepsia 43, 219–227. 11906505 10.1046/j.1528-1157.2002.26901.x

[brv70086-bib-0447] Sreenivasan, S. & Fiete, I. (2011). Grid cells generate an analog error‐correcting code for singularly precise neural computation. Nature Neuroscience 14, 1330–1337. 21909090 10.1038/nn.2901

[brv70086-bib-0448] Sreenivasan, V. , Menon, S. N. & Sinha, S. (2017). Emergence of coupling‐induced oscillations and broken symmetries in heterogeneously driven nonlinear reaction networks. Scientific Reports 7, 1594. 28487568 10.1038/s41598-017-01670-y PMC5431650

[brv70086-bib-0449] Stadler, B. M. R. , Stadler, P. F. , Wagner, G. P. & Fontana, W. (2001). The topology of the possible: formal spaces underlying patterns of evolutionary change. Journal of Theoretical Biology 213, 241–274. 11894994 10.1006/jtbi.2001.2423

[brv70086-bib-0450] Stadler, P. F. & Stadler, B. M. (2006). Genotype‐phenotype maps. Biological Theory 1, 268–279.

[brv70086-bib-0451] Stam, C. J. (2014). Modern network science of neurological disorders. Nature Reviews Neuroscience 15, 683–695. 25186238 10.1038/nrn3801

[brv70086-bib-0452] Stam, C. J. , Tewarie, P. , Van Dellen, E. , Van Straaten, E. C. W. , Hillebrand, A. & Van Mieghem, P. (2014). The trees and the forest: characterization of complex brain networks with minimum spanning trees. International Journal of Psychophysiology 92, 129–138. 24726900 10.1016/j.ijpsycho.2014.04.001

[brv70086-bib-0453] Stanford, W. , Mucha, P. J. & Dayan, E. (2024). Age‐related differences in network controllability are mitigated by redundancy in large‐scale brain networks. Communications Biology 7, 701. 38849512 10.1038/s42003-024-06392-2 PMC11161655

[brv70086-bib-0454] Stanford, W. C. , Mucha, P. J. & Dayan, E. (2022). A robust core architecture of functional brain networks supports topological resilience and cognitive performance in middle‐and old‐aged adults. Proceedings of the National Academy of Sciences of the United States of America 119, e2203682119. 36282912 10.1073/pnas.2203682119 PMC9636938

[brv70086-bib-0455] Stephan, K. E. , Friston, K. J. & Frith, C. D. (2009). Dysconnection in schizophrenia: from abnormal synaptic plasticity to failures of self‐monitoring. Schizophrenia Bulletin 35, 509–527. 19155345 10.1093/schbul/sbn176 PMC2669579

[brv70086-bib-0456] Sterling, P. (2020). What Is Health? Allostasis and the Evolution of Human Design. MIT Press. 10.1002/ajhb.23698 34779544

[brv70086-bib-0457] Stern, M. , Liu, A. J. & Balasubramanian, V. (2024). Physical effects of learning. Physical Review E 109, 24311. 10.1103/PhysRevE.109.024311 38491658

[brv70086-bib-0458] Stern, M. & Murugan, A. (2023). Learning without neurons in physical systems. Annual Review of Condensed Matter Physics 14, 417–441.

[brv70086-bib-0459] Stern, Y. , Barnes, C. A. , Grady, C. , Jones, R. N. & Raz, N. (2019). Brain reserve, cognitive reserve, compensation, and maintenance: operationalization, validity, and mechanisms of cognitive resilience. Neurobiology of Aging 83, 124–129. 31732015 10.1016/j.neurobiolaging.2019.03.022 PMC6859943

[brv70086-bib-0460] Stumpf, M. P. & Krakauer, D. C. (2000). Mapping the parameters of prion‐induced neuropathology. Proceedings of the National Academy of Sciences of the United States of America 97, 10573–10577. 10962032 10.1073/pnas.180317097 PMC27066

[brv70086-bib-0461] Styr, B. & Slutsky, I. (2018). Imbalance between firing homeostasis and synaptic plasticity drives early‐phase Alzheimer's disease. Nature Neuroscience 21, 463–473. 29403035 10.1038/s41593-018-0080-x PMC6533171

[brv70086-bib-0462] Sukenik, N. , Vinogradov, O. , Weinreb, E. , Segal, M. , Levina, A. & Moses, E. (2021). Neuronal circuits overcome imbalance in excitation and inhibition by adjusting connection numbers. Proceedings of the National Academy of Sciences of the United States of America 118, e2018459118. 33723048 10.1073/pnas.2018459118 PMC8000583

[brv70086-bib-0463] Sun, H. , Panda, R. K. , Verdel, R. , Rodriguez, A. , Dalmonte, M. & Bianconi, G. (2024). Network science: Ising states of matter. Physical Review E 109, 054305. 38907445 10.1103/PhysRevE.109.054305

[brv70086-bib-0464] Sun, J. & Motter, A. E. (2013). Controllability transition and nonlocality in network control. Physical Review Letters 110, 208701. 25167459 10.1103/PhysRevLett.110.208701

[brv70086-bib-0465] Susman, L. , Brenner, N. & Barak, O. (2019). Stable memory with unstable synapses. Nature Communications 10, 4441. 10.1038/s41467-019-12306-2 PMC6768856 31570719

[brv70086-bib-0466] Sutula, T. P. , Hagen, J. & Pitkänen, A. (2003). Do epileptic seizures damage the brain? Current Opinion in Neurology 16, 189–195. 12644748 10.1097/01.wco.0000063770.15877.bc

[brv70086-bib-0467] Sydnor, V. J. , Larsen, B. , Bassett, D. S. , Alexander‐Bloch, A. , Fair, D. A. , Liston, C. , Mackey, A. P. , Milham, M. P. , Pines, A. , Roalf, D. R. & Seidlitz, J. (2021). Neurodevelopment of the association cortices: patterns, mechanisms, and implications for psychopathology. Neuron 109, 2820–2846. 34270921 10.1016/j.neuron.2021.06.016 PMC8448958

[brv70086-bib-0468] Tadić, B. , Rodgers, G. J. & Thurner, S. (2007). Transport on complex networks: flow, jamming and optimization. International Journal of Bifurcation and Chaos 17, 2363–2385.

[brv70086-bib-0469] Taleb, N. N. (2012). Antifragile. Random House, New York.

[brv70086-bib-0470] Tanaka, G. , Morino, K. & Aihara, K. (2012). Dynamical robustness in complex networks: the crucial role of low‐degree nodes. Scientific Reports 2, 232. 22355746 10.1038/srep00232 PMC3265565

[brv70086-bib-0471] Tanaka, G. , Morino, K. & Aihara, K. (2015). Dynamical robustness of complex biological networks. In Mathematical Approaches to Biological Systems: Networks, Oscillations, and Collective Motions, pp. 29–53.

[brv70086-bib-0472] Tanaka, G. , Morino, K. , Daido, H. & Aihara, K. (2014). Dynamical robustness of coupled heterogeneous oscillators. Physical Review E 89, 52906. 10.1103/PhysRevE.89.052906 25353860

[brv70086-bib-0473] Teller, S. , Estévez‐Priego, E. , Granell, C. , Tornero, D. , Andilla, J. , Olarte, O. E. , Loza‐Alvarez, P. , Arenas, A. & Soriano, J. (2020). Spontaneous functional recovery after focal damage in neuronal cultures. eNeuro 7, ENEURO.0254–19.2019. 10.1523/ENEURO.0254-19.2019 PMC6984807 31818830

[brv70086-bib-0474] Tellez‐Zenteno, J. F. , Ronquillo, L. H. , Moien‐Afshari, F. & Wiebe, S. (2010). Surgical outcomes in lesional and non‐lesional epilepsy: a systematic review and meta‐analysis. Epilepsy Research 89, 310–318. 20227852 10.1016/j.eplepsyres.2010.02.007

[brv70086-bib-0475] Terry, J. R. , Benjamin, O. & Richardson, M. P. (2012). Seizure generation: the role of nodes and networks. Epilepsia 53, e166–e169. 22709380 10.1111/j.1528-1167.2012.03560.x

[brv70086-bib-0476] Thakur, B. , Sharma, D. & Sen, A. (2014). Time‐delay effects on the aging transition in a population of coupled oscillators. Physical Review E 90, 042904. 10.1103/PhysRevE.90.042904 25375564

[brv70086-bib-0477] Thomas, R. (1981). On the relation between the logical structure of systems and their ability to generate multiple steady states and sustained oscillations. In Series in Synergetics (Volume 9), pp. 180–193. Springer.

[brv70086-bib-0478] Thompson, T. B. , Chaggar, P. , Kuhl, E. & Goriely, A. (2020). Protein–protein interactions in neurodegenerative diseases: a conspiracy theory. PLoS Computational Biology 16, e1008267. 33048932 10.1371/journal.pcbi.1008267 PMC7584458

[brv70086-bib-0479] Thouless, D. J. , Kohmoto, M. , Nightingale, M. P. & den Nijs, M. (1982). Quantized Hall conductance in a two‐dimensional periodic potential. Physical Review Letters 49, 405–408.

[brv70086-bib-0480] Tian, T. , Olson, S. , Whitacre, J. M. & Harding, A. (2011). The origins of cancer robustness and evolvability. Integrative Biology 3, 17–30. 20944865 10.1039/c0ib00046a

[brv70086-bib-0481] Tijms, B. M. , Wink, A. M. , De Haan, W. , van der Flier, W. M. , Stam, C. J. , Scheltens, P. & Barkhof, F. (2013). Alzheimer's disease: connecting findings from graph theoretical studies of brain networks. Neurobiology of Aging 34, 2023–2036. 23541878 10.1016/j.neurobiolaging.2013.02.020

[brv70086-bib-0482] Timme, M. (2006). Does dynamics reflect topology in directed networks? Europhysics Letters 76, 367–373.

[brv70086-bib-0483] Timme, M. (2007). Revealing network connectivity from response dynamics. Physical Review Letters 98, 224101. 17677845 10.1103/PhysRevLett.98.224101

[brv70086-bib-0484] Tononi, G. & Edelman, G. M. (2000). Schizophrenia and the mechanisms of conscious integration. Brain Research Reviews 31, 391–400. 10719167 10.1016/s0165-0173(99)00056-9

[brv70086-bib-0485] Tononi, G. , Sporns, O. & Edelman, G. M. (1994). A measure for brain complexity: relating functional segregation and integration in the nervous system. Proceedings of the National Academy of Sciences of the United States of America 91, 5033–5037. 8197179 10.1073/pnas.91.11.5033 PMC43925

[brv70086-bib-0486] Tononi, G. , Sporns, O. & Edelman, G. M. (1999). Measures of degeneracy and redundancy in biological networks. Proceedings of the National Academy of Sciences of the United States of America 96, 3257–3262. 10077671 10.1073/pnas.96.6.3257 PMC15929

[brv70086-bib-0487] Torres, B. Y. , Oliveira, J. H. M. , Thomas Tate, A. , Rath, P. , Cumnock, K. & Schneider, D. S. (2016). Tracking resilience to infections by mapping disease space. PLoS Biology 14, e1002436. 27088359 10.1371/journal.pbio.1002436 PMC4835107

[brv70086-bib-0488] Tosi, G. , Nigro, S. , Urso, D. , Spinosa, V. , Gnoni, V. , Filardi, M. , Giaquinto, F. , Rizzi, E. , Iaia, M. , Macchitella, L. & Chiarello, Y. (2024). The network structure of cognitive impairment: from subjective cognitive decline to Alzheimer's disease. The Journal of Neuroscience 44, e1344232023. 38830757 10.1523/JNEUROSCI.1344-23.2023 PMC11223460

[brv70086-bib-0489] Transtrum, M. K. , Machta, B. B. , Brown, K. S. , Daniels, B. C. , Myers, C. R. & Sethna, J. P. (2015). Perspective: sloppiness and emergent theories in physics, biology, and beyond. Journal of Chemical Physics 143, 010901. 26156455 10.1063/1.4923066

[brv70086-bib-0490] Tsuda, I. (2001). Toward an interpretation of dynamic neural activity in terms of chaotic dynamical systems. Behavioral and Brain Sciences 24, 793–810. 12239890 10.1017/s0140525x01000097

[brv70086-bib-0491] Tu, C. , Rocha, R. P. , Corbetta, M. , Zampieri, S. , Zorzi, M. & Suweis, S. (2018). Warnings and caveats in brain controllability. NeuroImage 176, 83–91. 29654874 10.1016/j.neuroimage.2018.04.010 PMC6607911

[brv70086-bib-0492] Turrigiano, G. (2011). Too many cooks? Intrinsic and synaptic homeostatic mechanisms in cortical circuit refinement. Annual Review of Neuroscience 34, 89–103. 10.1146/annurev-neuro-060909-153238 21438687

[brv70086-bib-0493] Turrigiano, G. G. , Leslie, K. R. , Desai, N. S. , Rutherford, L. C. & Nelson, S. B. (1998). Activity‐dependent scaling of quantal amplitude in neocortical neurons. Nature 391, 892–896. 9495341 10.1038/36103

[brv70086-bib-0494] Tyloo, M. , Coletta, T. & Jacquod, P. (2018). Robustness of synchrony in complex networks and generalized Kirchhoff indices. Physical Review Letters 120, 084101. 29542999 10.1103/PhysRevLett.120.084101

[brv70086-bib-0495] Urruty, N. , Tailliez‐Lefebvre, D. & Huyghe, C. (2016). Stability, robustness, vulnerability and resilience of agricultural systems. A review. Agronomy for Sustainable Development 36, 1–15.

[brv70086-bib-0496] van den Heuvel, M. P. , Mandl, R. C. , Stam, C. J. , Kahn, R. S. & Pol, H. E. H. (2010). Aberrant frontal and temporal complex network structure in schizophrenia: a graph theoretical analysis. The Journal of Neuroscience 30, 15915–15926. 21106830 10.1523/JNEUROSCI.2874-10.2010 PMC6633761

[brv70086-bib-0497] van den Heuvel, M. P. , Sporns, O. , Collin, G. , Scheewe, T. , Mandl, R. C. , Cahn, W. , Goñi, J. , Pol, H. E. H. & Kahn, R. S. (2013). Abnormal rich club organization and functional brain dynamics in schizophrenia. JAMA Psychiatry 70, 783–792. 23739835 10.1001/jamapsychiatry.2013.1328

[brv70086-bib-0498] van Diessen, E. , Diederen, S. J. , Braun, K. P. , Jansen, F. E. & Stam, C. J. (2013). Functional and structural brain networks in epilepsy: what have we learned? Epilepsia 54, 1855–1865. 24032627 10.1111/epi.12350

[brv70086-bib-0499] Van Essen, D. C. , Smith, S. M. , Barch, D. M. , Behrens, T. E. , Yacoub, E. , Ugurbil, K. & Wu‐Minn HCP Consortium (2013). The WU‐Minn human connectome project: an overview. NeuroImage 80, 62–79. 23684880 10.1016/j.neuroimage.2013.05.041 PMC3724347

[brv70086-bib-0500] Van Essen, D. C. , Ugurbil, K. , Auerbach, E. , Barch, D. , Behrens, T. E. , Bucholz, R. , Chang, A. , Chen, L. , Corbetta, M. , Curtiss, S. W. , Della Penna, S. , Feinberg, D. , Glasser, M. F. , Harel, N. , Heath, A. C. , *et al*. (2012). The human connectome project: a data acquisition perspective. NeuroImage 62, 2222–2231. 22366334 10.1016/j.neuroimage.2012.02.018 PMC3606888

[brv70086-bib-0501] van Haren, N. E. M. , Pol, H. E. H. , Schnack, H. G. , Cahn, W. , Brans, R. , Carati, I. , Rais, M. & Kahn, R. S. (2008). Progressive brain volume loss in schizophrenia over the course of the illness: evidence of maturational abnormalities in early adulthood. Biological Psychiatry 63, 106–113. 17599810 10.1016/j.biopsych.2007.01.004

[brv70086-bib-0502] van Meegen, A. , Kühn, T. & Helias, M. (2021). Large‐deviation approach to random recurrent neuronal networks: parameter inference and fluctuation‐induced transitions. Physical Review Letters 127, 158302. 34678014 10.1103/PhysRevLett.127.158302

[brv70086-bib-0503] van Nimwegen, E. , Crutchfield, J. P. & Huynen, M. (1999). Neutral evolution of mutational robustness. Proceedings of the National Academy of Sciences of the United States of America 96, 9716–9720. 10449760 10.1073/pnas.96.17.9716 PMC22276

[brv70086-bib-0504] van Vreeswijk, C. & Sompolinsky, H. (1996). Chaos in neuronal networks with balanced excitatory and inhibitory activity. Science 274, 1724–1726. 8939866 10.1126/science.274.5293.1724

[brv70086-bib-0505] Vasa, R. A. , Mostofsky, S. H. & Ewen, J. B. (2016). The disrupted connectivity hypothesis of autism spectrum disorders: time for the next phase in research. Biological Psychiatry: Cognitive Neuroscience and Neuroimaging 1, 245–252. 28083565 10.1016/j.bpsc.2016.02.003 PMC5222574

[brv70086-bib-0506] Vattikuti, S. & Chow, C. C. (2010). A computational model for cerebral cortical dysfunction in autism spectrum disorders. Biological Psychiatry 67, 672–678. 19880095 10.1016/j.biopsych.2009.09.008 PMC3104404

[brv70086-bib-0507] Verstraete, E. , Veldink, J. H. , Mandl, R. C. , van den Berg, L. H. & Heuvel, M. P. (2011). Impaired structural motor connectome in amyotrophic lateral sclerosis. PLoS One 6, e24239. 21912680 10.1371/journal.pone.0024239 PMC3166305

[brv70086-bib-0508] Villegas, P. , Gabrielli, A. , Santucci, F. , Caldarelli, G. & Gili, T. (2022). Laplacian paths in complex networks: information core emerges from entropic transitions. Physical Review Research 4, 033196.

[brv70086-bib-0509] Villegas, P. , Moretti, P. & Muñoz, M. A. (2014). Frustrated hierarchical synchronization and emergent complexity in the human connectome network. Scientific Reports 4, 5990. 25103684 10.1038/srep05990 PMC4126002

[brv70086-bib-0510] Viscomi, M. T. & Molinari, M. (2014). Remote neurodegeneration: multiple actors for one play. Molecular Neurobiology 50, 368–389. 24442481 10.1007/s12035-013-8629-x

[brv70086-bib-0511] Wagner, A. (2008 *a*). Neutralism and selectionism: a network‐based reconciliation. Nature Reviews Genetics 9, 965–974. 10.1038/nrg2473 18957969

[brv70086-bib-0512] Wagner, A. (2008 *b*). Robustness and evolvability: a paradox resolved. Proceedings of the Royal Society B: Biological Sciences 275, 91–100. 10.1098/rspb.2007.1137 PMC2562401 17971325

[brv70086-bib-0513] Wagner, A. (2011). Genotype networks shed light on evolutionary constraints. Trends in Ecology & Evolution 26, 577–583. 21840080 10.1016/j.tree.2011.07.001

[brv70086-bib-0514] Wagner, A. (2012). The role of robustness in phenotypic adaptation and innovation. Proceedings of the Royal Society B: Biological Sciences 279, 1249–1258. 10.1098/rspb.2011.2293 PMC3282381 22217723

[brv70086-bib-0515] Walker, B. , Holling, C. S. , Carpenter, S. R. & Kinzig, A. (2004). Resilience, adaptability and transformability in social–ecological systems. Ecology and Society 9, 5.

[brv70086-bib-0516] Wang, L. , Yu, C. , Chen, H. , Qin, W. , He, Y. , Fan, F. , Zhang, Y. , Wang, M. , Li, K. , Zang, Y. & Woodward, T. S. (2010). Dynamic functional reorganization of the motor execution network after stroke. Brain 133, 1224–1238. 20354002 10.1093/brain/awq043

[brv70086-bib-0517] Wang, W. , Li, W. , Lin, T. , Wu, T. , Pan, L. & Liu, Y. (2022). Generalized k‐core percolation on higher‐order dependent networks. Applied Mathematics and Computation 420, 126793.

[brv70086-bib-0518] Wang, X. J. & Kennedy, H. (2016). Brain structure and dynamics across scales: in search of rules. Current Opinion in Neurobiology 37, 92–98. 26868043 10.1016/j.conb.2015.12.010 PMC5029120

[brv70086-bib-0519] Warren, D. E. , Power, J. D. , Bruss, J. , Denburg, N. L. , Waldron, E. J. , Sun, H. , Petersen, S. E. & Tranel, D. (2014). Network measures predict neuropsychological outcome after brain injury. Proceedings of the National Academy of Sciences of the United States of America 111, 14247–14252. 25225403 10.1073/pnas.1322173111 PMC4191760

[brv70086-bib-0520] Warren, J. D. , Rohrer, J. D. , Schott, J. M. , Fox, N. C. , Hardy, J. & Rossor, M. N. (2013). Molecular nexopathies: a new paradigm of neurodegenerative disease. Trends in Neurosciences 36, 561–569. 23876425 10.1016/j.tins.2013.06.007 PMC3794159

[brv70086-bib-0521] Weinberger, D. R. (1993). A connectionist approach to the prefrontal cortex. The Journal of Neuropsychiatry and Clinical Neurosciences 5, 241–253. 8369632 10.1176/jnp.5.3.241

[brv70086-bib-0522] Wen, W. & Turrigiano, G. G. (2024). Keeping your brain in balance: homeostatic regulation of network function. Annual Review of Neuroscience 47, 41–61. 10.1146/annurev-neuro-092523-110001 38382543

[brv70086-bib-0523] Wen, X. G. (1995). Topological orders and edge excitations in fractional quantum hall states. Advances in Physics 44, 405–473.

[brv70086-bib-0524] West, B. J. (2010). Fractal physiology and the fractional calculus: a perspective. Frontiers in Physiology 1, 12. 21423355 10.3389/fphys.2010.00012 PMC3059975

[brv70086-bib-0525] Wilkat, T. , Rings, T. & Lehnertz, K. (2019). No evidence for critical slowing down prior to human epileptic seizures. Chaos 29, 091104. 31575122 10.1063/1.5122759

[brv70086-bib-0526] Wilson, R. S. , Yu, L. , Lamar, M. , Schneider, J. A. , Boyle, P. A. & Bennett, D. A. (2019). Education and cognitive reserve in old age. Neurology 92, e1041–e1050. 30728309 10.1212/WNL.0000000000007036 PMC6442015

[brv70086-bib-0527] Witthaut, D. , Rohden, M. , Zhang, X. , Hallerberg, S. & Timme, M. (2016). Critical links and nonlocal rerouting in complex supply networks. Physical Review Letters 116, 138701. 27082006 10.1103/PhysRevLett.116.138701

[brv70086-bib-0528] Wu, X. , Fu, Y. , Knott, G. , Lu, J. , Di Cristo, G. & Huang, Z. J. (2012). GABA signaling promotes synapse elimination and axon pruning in developing cortical inhibitory interneurons. Journal of Neuroscience 32, 331–343. 22219294 10.1523/JNEUROSCI.3189-11.2012 PMC3742883

[brv70086-bib-0529] Wu, Y. K. , Hengen, K. B. , Turrigiano, G. G. & Gjorgjieva, J. (2020). Homeostatic mechanisms regulate distinct aspects of cortical circuit dynamics. Proceedings of the National Academy of Sciences of the United States of America 117, 24514–24525. 32917810 10.1073/pnas.1918368117 PMC7533694

[brv70086-bib-0530] Xue, L. , Gao, S. , Gallos, L. K. , Levy, O. , Gross, B. , Di, Z. & Havlin, S. (2024). Nucleation phenomena and extreme vulnerability of spatial k‐core systems. Nature Communications 15, 5850. 10.1038/s41467-024-50273-5 PMC11239893 38992015

[brv70086-bib-0531] Yan, G. , Tsekenis, G. , Barzel, B. , Slotine, J. J. , Liu, Y. Y. & Barabási, A. L. (2015). Spectrum of controlling and observing complex networks. Nature Physics 11, 779–786.

[brv70086-bib-0532] Yizhar, O. , Fenno, L. E. , Prigge, M. , Schneider, F. , Davidson, T. J. , O'Shea, D. J. , Sohal, V. S. , Goshen, I. , Finkelstein, J. , Paz, J. T. & Stehfest, K. (2011). Neocortical excitation/inhibition balance in information processing and social dysfunction. Nature 477, 171–178. 21796121 10.1038/nature10360 PMC4155501

[brv70086-bib-0533] Zamora‐López, G. , Zhou, C. & Kurths, J. (2010). Cortical hubs form a module for multisensory integration on top of the hierarchy of cortical networks. Frontiers in Neuroinformatics 4, 613. 10.3389/neuro.11.001.2010 PMC2859882 20428515

[brv70086-bib-0534] Zanin, M. , Alcazar, J. M. , Carbajosa, J. V. , Paez, M. G. , Papo, D. , Sousa, P. , Menasalvas, E. & Boccaletti, S. (2014). Parenclitic networks: uncovering new functions in biological data. Scientific Reports 4, 5112. 24870931 10.1038/srep05112 PMC4037713

[brv70086-bib-0535] Zanin, M. , Menasalvas, E. , Sun, X. & Wandelt, S. (2018). From the difference of structures to the structure of the difference. Complexity 2018, 4326097.

[brv70086-bib-0536] Zañudo, J. G. T. & Albert, R. (2013). An effective network reduction approach to find the dynamical repertoire of discrete dynamic networks. Chaos 23, 025111. 23822509 10.1063/1.4809777

[brv70086-bib-0537] Zañudo, J. G. T. , Yang, G. & Albert, R. (2017). Structure‐based control of complex networks with nonlinear dynamics. Proceedings of the National Academy of Sciences of the United States of America 114, 7234–7239. 28655847 10.1073/pnas.1617387114 PMC5514702

[brv70086-bib-0538] Zaveri, H. P. , Schelter, B. , Schevon, C. A. , Jiruska, P. , Jefferys, J. G. , Worrell, G. , Schulze‐Bonhage, A. , Joshi, R. B. , Jirsa, V. , Goodfellow, M. & Meisel, C. (2020). Controversies on the network theory of epilepsy: debates held during the ICTALS 2019 conference. Seizure 78, 78–85. 32272333 10.1016/j.seizure.2020.03.010 PMC7952007

[brv70086-bib-0539] Zenke, F. , Gerstner, W. & Ganguli, S. (2017). The temporal paradox of Hebbian learning and homeostatic plasticity. Current Opinion in Neurobiology 43, 166–176. 28431369 10.1016/j.conb.2017.03.015

[brv70086-bib-0540] Zeraati, R. , Priesemann, V. & Levina, A. (2020). Self‐organization toward criticality by synaptic plasticity. Frontiers in Physics 9, 619661.

[brv70086-bib-0541] Zhang, D. & Raichle, M. E. (2010). Disease and the brain's dark energy. Nature Reviews. Neurology 6, 15–28. 20057496 10.1038/nrneurol.2009.198

[brv70086-bib-0542] Zhang, Y. , Garas, A. & Scholtes, I. (2021). Higher‐order models capture changes in controllability of temporal networks. Journal of Physics: Complexity 2, 015007.

[brv70086-bib-0543] Zhou, X. , Menche, J. , Barabási, A.‐L. & Sharma, A. (2014). Human symptoms‐disease network. Nature Communications 5, 4212. 10.1038/ncomms5212 24967666

[brv70086-bib-0544] Zierenberg, J. , Wilting, J. & Priesemann, V. (2018). Homeostatic plasticity and external input shape neural network dynamics. Physical Review X 8, 031018.

[brv70086-bib-0545] Zonca, L. , Escrichs, A. , Patow, G. A. , Manasova, D. , Sanz Perl, Y. , Annen, J. , Gosseries, O. , Deco, G. & Sitt, J. (2024). Modeling disorders of consciousness at the patient level reveals the network's influence on the diagnosis vs the local node parameters role in prognosis. bioRxiv 2024‐09.

[brv70086-bib-0546] Zylberberg, J. , Pouget, A. , Latham, P. E. & Shea‐Brown, E. (2017). Robust information propagation through noisy neural circuits. PLoS Computational Biology 13, e1005497. 28419098 10.1371/journal.pcbi.1005497 PMC5413111

